# Marine invertebrates associated with rhodoliths/maërl beds from northeast Brazil (State of Paraíba)

**DOI:** 10.3897/BDJ.9.e62736

**Published:** 2021-07-21

**Authors:** Dimítri de Araújo Costa, Marina Dolbeth, Jessica Prata, Francisco de Assis da Silva, Geuba Maria Bernardo da Silva, Paulo Ragner Silva de Freitas, Martin Lindsey Christoffersen, Silvio Felipe Barbosa de Lima, Karina Massei, Reinaldo Farias Paiva de Lucena

**Affiliations:** 1 CIIMAR - Interdisciplinary Centre of Marine and Environmental Research, Matosinhos, Portugal CIIMAR - Interdisciplinary Centre of Marine and Environmental Research Matosinhos Portugal; 2 UFPB - Federal University of Paraíba, DSE - Department of Systematics and Ecology, João Pessoa, Brazil UFPB - Federal University of Paraíba, DSE - Department of Systematics and Ecology João Pessoa Brazil; 3 Sea Servin, Aquário Paraíba, João Pessoa, Brazil Sea Servin, Aquário Paraíba João Pessoa Brazil; 4 InPact - Interinstitutional Relations of the Research and Action Institute, João Pessoa, Brazil InPact - Interinstitutional Relations of the Research and Action Institute João Pessoa Brazil; 5 UFPB - Federal University of Paraíba, DCB - Department of Biological Sciences, Areia, Brazil UFPB - Federal University of Paraíba, DCB - Department of Biological Sciences Areia Brazil; 6 IFPI - Federal Institute of Education, Science and Technology of Piauí, Uruçuí, Brazil IFPI - Federal Institute of Education, Science and Technology of Piauí Uruçuí Brazil; 7 UFCG - Federal University of Campina Grande, CFP - Centro de Formação de Professores, UACEN - Unidade Acadêmica de Ciências Exatas e da Natureza, Cajazeiras, Brazil UFCG - Federal University of Campina Grande, CFP - Centro de Formação de Professores, UACEN - Unidade Acadêmica de Ciências Exatas e da Natureza Cajazeiras Brazil

**Keywords:** biodiversity, new records, distribution, calcareous red algae, tropical beaches, South Atlantic coast

## Abstract

**Background:**

This study investigates the marine macroinvertebrate fauna of rhodolith beds (non-geniculated red corallinaceaous algae) in northeast Brazilian. A total of 57 species were identified, belonging to six phyla (Platyhelminthes, Annelida, Sipuncula, Mollusca, Arthropoda and Echinodermata), of which 50 are considered here as new records for the Paraíba State. Annelids (Class Polychaeta) were the most representative taxa in Miramar and Seixas Beaches, while molluscs were dominant in Maceió Beach.

**New information:**

This is the first study that includes an identification key, diagnostic features and distribution patterns worldwide and local (including new records) of the marine invertebrate fauna associated with rhodolith beds in northeast Brazil (State of Paraíba). Sampling events were performed in 2018, at low tide in the intertidal to shallow subtidal zones (1.5 and 4.0 m depth), in Miramar, Seixas and Maceió Beaches. A total of 17 species were found for the first time on Seixas Beach, as well as all identified species for Miramar and Maceió. This study tries to contribute to the knowledge of marine invertebrates in northeast Brazilian shallow habitats, providing a baseline for future environmental studies.

## Introduction

Marine invertebrates are a group of animals characterised by the absence of a backbone, living in the oceanic zone around the world, from the intertidal region to great depths. The fauna is represented by many taxa, for example, poriferans, cnidarians, annelids, crustaceans, molluscs and echinoderms, with a great variety of morphological characteristics, types of behaviour, feeding habits, participating in all levels of food webs, being essential for the maintenance of homeorhesis and equilibrium in the oceans.

The red non-articulated calcareous algae, known as rhodoliths or maërl (European name), are reported in all oceans, from the intertidal zone to depths of 270 m and these habitat-like structures are considered as a hotspot of biodiversity, harbouring many groups of invertebrates, mainly juveniles (Scherner et al. 2010), for example, polychaetes, crustaceans, molluscs, sipunculids and echinoderms (Prata et al. 2017). The Brazilian coast, in particular the northeastern region, may represent the zone with the highest abundance of rhodoliths worldwide, due to the large deposits of calcium carbonate (Amado-Filho et al. 2012).

The present study aimed to describe, by diagnostic characteristics, the marine invertebrates associated with rhodoliths of three beaches from the State of Paraíba, northeast Brazil, with the inclusion of global and local distribution.

## Materials and methods

### Study area

The Brazilian coast zone extends for 8,500 km and has a width of 12 nautical miles outwards from the coast (MMA (Ministério do Meio Ambiente da República Federativa do Brasil) 2020). The northeast zone has around to 3,400 km, in which Paraíba is one of the States of this zone, having a coastline that extends for 140 km (Lima and Heckendorff 1985), from the Estuary of Guajú River (in the north) up to the Estuary of Goiana River (in the south) (Neves et al. 2006).

**Figure 1.**
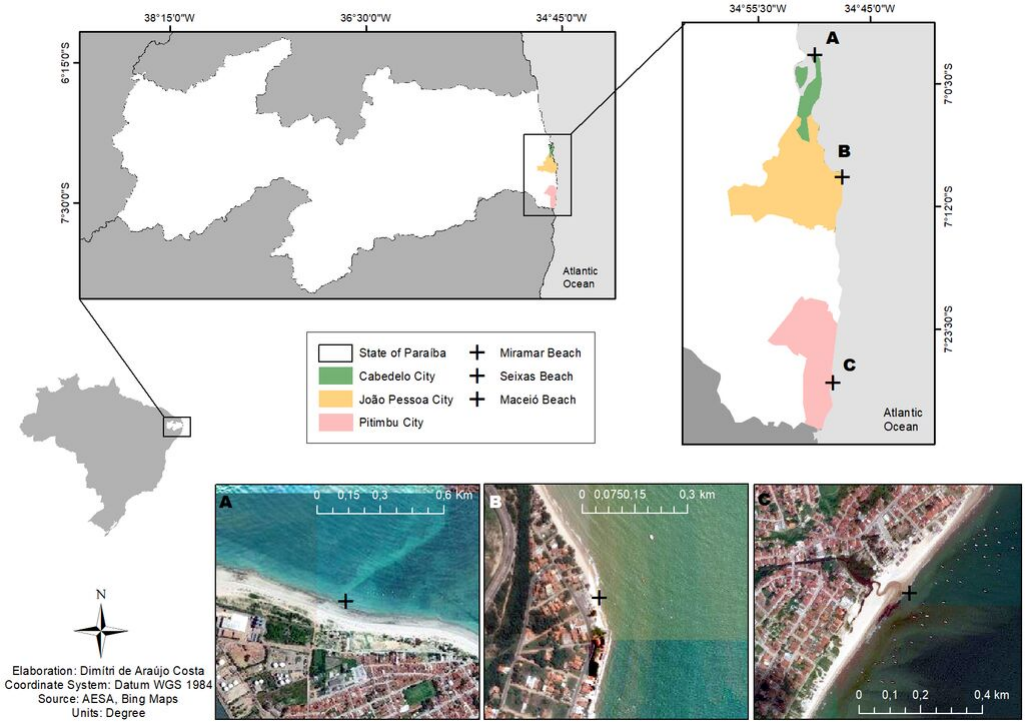
Study area, including Municipalities and beaches in the State of Paraíba coast, northeast Brazil. **A.** Miramar Beach (1.5 m: 6°57'51"S/34°50'01"W; 4.0 m: 6°57'50"S/34°50'01"W); **B.** Seixas Beach (1.5 m: 7°09'18"S/34°47'35"W; 4.0 m: 7°09'18"S; 34°47'33"W); **C.** Maceió Beach (1.5 m: 7°28'34"S/34°48'27"W; 4.0 m: 7°28'34"S/34°48'26"W).

The sampling campaigns were carried out in 2018, at the coastal area of the Paraíba State, at Miramar (Cabedelo Municipality), at Seixas (João Pessoa) and at Maceió (Pitimbu) Beaches (Fig. 1), on the subtidal zone, considering two depths in each site, 1.5 and 4.0 m.

### Methodological approach

The habitats of the sampling area are constituted of rhodoliths, i.e. red calcareous non-articulated algae from the subclass Corallinophycidae. The sampling procedure design for the rhodoliths followed the quadrat methodology, adapted from Underwood and Chapman (2013). At each Beach, a 100 m^2^ quadrat was selected at a shallower zone depth (1.5 m) and another at a deeper depth (4.0 m), with 20 metres distance apart, from which five replicates were chosen with 225 cm^2^ area each. The replicates were chosen at each of the quadrat edges and in the centre point (for analysis, each sampling area was considered as one sample, being two samples per beach and six in total). Through scuba diving, an aluminium quadrant was placed on the bottom sediment of each sampling area and all the rhodoliths inside the replicate’s squares were collected manually with gloves and stored inside plastic bags (no sediments), which were immediately closed. These samples were accommodated in thermal boxes with ice and taken to the laboratory for processing.

The biological material was sorted at the “Laboratório de Invertebrados Paulo Young” (LIPY [Paulo Young Invertebrate Laboratory]) from the “Universidade Federal da Paraíba (UFPB, Campus I, Brazil [Federal University of Paraíba])”. The collected invertebrates were removed, fixed and stored in 70% alcohol, being identified to the specific taxonomic level. The species were photographed with a Canon 6d digital camera–length 100 mm macro L or a Leica MZ12.5 stereomicroscope. Nevertheless, the photos were taken for general overview of the organisms identified, not with the intention of documenting detailed taxonomic characters for specific identification.

The identified taxonomic groups were organised according to the World Register of Marine Species (WoRMS-Editorial-Board 2020), with additional information for polychaetes (Rouse and Fauchald 1997). Their distribution in Paraiba is based on the distribution records from published sources, including the present study.

All specimens were deposited in the “Coleção Zoológica Aquário Paraíba” – CZAP (‘Zoological Collection Aquário Paraíba’), João Pessoa Municipality, northeast Brazil. The collection of invertebrates was authorised by the “Sistema de Autorização e Informação em Biodiversidade” (SISBIO), “Instituto Chico Mendes de Conservação da Biodiversidade” (ICMBio), request nº 63971, report nº 25753, Ministry of Environment, from the Federative Republic of Brazil (Suppl. material 1).

## Data resources

The data underpinning the analysis reported in this paper are deposited in the Dryad Data Repository at https://doi.org/10.5061/dryad.fbg79cnv8.

## Checklists

### Diagnosis and distribution of identified marine invertebrates associated with rhodoliths from northeast Brazil

#### Platyhelminthes

Minot, 1876

F4DC2ED5-A593-56B6-BE76-921E8E76723F

#### Polycladida

Lang, 1884

C2E639B3-A118-5C83-887F-09B0B169893E

#### Prosthiostomidae

Lang, 1884

82E6E8BF-383F-5118-8413-7C9228855A50

#### Enchiridium evelinae

Marcus, 1949

829A8678-EEE8-5426-8D80-04F18FC60ED4

https://www.marinespecies.org/aphia.php?p=taxdetails&id=483828

##### Materials

- **Type status:**Other material. **Occurrence:** catalogNumber: CZAP–102; recordedBy: G. da Silva, D. Costa; individualCount: 2; **Location:** locality: Seixas Beach; verbatimDepth: 1.5 m

##### Distribution

Brazil (Paraíba, Rio Grande do Norte, Alagoas, Rio de Janeiro and São Paulo States) (Bahia et al. 2015, Tyler et al. 2020; and this study).

**Distribution in Paraíba**: Seixas Beach (**New record**). This species represents a new record from the State of Paraíba.

##### Notes

Found on the rhodoliths surface.

##### Diagnosis

(Bahia et al. 2014): Flatworm of free-living, long and narrow body; dorsal region cream with brown dots, more densely disposed at the median line; margin with orange dots (Fig. 2a); pharynx reaches 1/3 of the body length; seminal and prostatic vesicles highly muscularized; penis papilla and male atrium long.

#### Annelida

Lamarck, 1809

6216D471-FBCC-5096-BAB2-EC1C281E3BB2

#### Polychaeta

Grube, 1850

85D9EE62-B9B0-51C0-B76B-DF69793B1DB4

#### Errantia

Audouin & Milne Edwards, 1832

4952602B-378E-5DF9-8501-F6FDB103F695

#### Amphinomida

Fauchald, 1977

865C1420-ADCA-519E-966C-355EF8D9BE30

#### Amphinomidae

Lamarck, 1818

10626D89-3D75-51EE-874E-FBBCB5C8331B

#### Eurythoe complanata

(Pallas, 1766)

4F66D127-32B0-5AF9-88D7-91B67268BE60

https://www.marinespecies.org/aphia.php?p=taxdetails&id=129829

##### Materials

- **Type status:**Other material. **Occurrence:** catalogNumber: CZAP–169; recordedBy: G. da Silva, D. Costa; individualCount: 4; **Location:** locality: Miramar Beach; verbatimDepth: 4.0 m

##### Distribution

Caribbean Sea to Brazil (Ceará, Rio Grande do Norte, Paraíba, Pernambuco, Alagoas, Bahia, Espírito Santo, Rio de Janeiro and São Paulo States); Iberian Peninsula to Red Sea; Azores Archipelago; East Africa; Pacific Ocean (Oceania to South America and Hawaii) (Amaral et al. 2013, Costa et al. 2017, Read and Fauchald 2020a).

**Distribution in Paraíba**: Barra de Camaratuba Beach, Mamanguape River, Cabo Branco Beach (DeAssis et al. 2012), Seixas Beach (Costa et al. 2017) and Miramar Beach (**New record**).

##### Notes

Found inside the rhodoliths.

##### Diagnosis

(Arias et al. 2013, Barroso and Paiva 2007): This species carries a prostomium with four eyes (trapezoidally arranged), three smooth antennae, two cirriform palps and a fleshy dorsal protuberance known as caruncle, extending to the third chaetiger. Branchiae ramified from chaetiger 2. Each parapodium with two slender/digitiform cirri (dorsal and ventral, similar in size, Fig. 2b); notopodia (dorsal) with the following kinds of chaetae: furcate, smooth, serrated and a slender blade with a small spur; neuropodia (ventral) with furcate chaetae.

#### Eunicida

Fauchald, 1977

E213D28D-3826-5C82-BF40-C81A39BC108F

#### Eunicidae

Berthold, 1827

5C9AA8F5-CCFC-5637-A3E4-1B7FACDBB08E

#### Eunice biannulata

Moore, 1904

9F505C86-7848-54FA-9C08-2A33E7DA44CF

https://www.marinespecies.org/aphia.php?p=taxdetails&id=327643

##### Materials

- **Type status:**Other material. **Occurrence:** catalogNumber: (CZAP–246, CZAP–232), (CZAP–092, CZAP–183); recordedBy: G. da Silva, D. Costa; individualCount: (2, 4), (21, 1); **Location:** locality: Miramar and Seixas Beaches; verbatimDepth: (1.5 m, 4.0 m), (1.5 m, 4.0 m)

##### Distribution

Pacific coast from United States of America, Brazil (Rio Grande do Norte, Paraíba, Alagoas, Sergipe, Bahia and Paraná States) (Amaral et al. 2013, Costa et al. 2017, Nonato and Luna 1970, Read and Fauchald 2020b).

**Distribution in Paraíba**: Miramar Beach (**New record**), Seixas Beach (Costa et al. 2017; and this study).

##### Notes

Found inside the rhodoliths.

##### Diagnosis

(Fauchald 1992, Paxton 2009): Prostomium with a median sulcus deep, with four eyes (arranged in a curved line) and five segmented antennae (Fig. 2c). Jaws eulabidognath-type (asymmetrical, posterior parts dentate to forceps-like, short carriers). Formula (maxillae): 1+1, 5 to 6+5 to 6, 6+0, 6+10 and 1+1. Peristomium with two segmented cirri. Branchiae pectinate (1 to 8 filaments) from chaetiger 3. Parapodia with notopodial medially inflated cirri, with articulations (anterior and median ones) or without articulations (posterior ones), larger than ventral ones; neuropodia carry anterior inflated basally cirri and posterior ones digitiform, pre- and post-chaetal lobes, limbate, pectinate and falcigers chaetae, yellow aciculae paired and yellow subacicular bidentate hooks.

#### Eunice wasinensis

Fauchald, 1992

35EE3407-0589-5448-853A-C8088E6C6B43

https://www.marinespecies.org/aphia.php?p=taxdetails&id=327813

##### Materials

- **Type status:**Other material. **Occurrence:** catalogNumber: (CZAP–228, CZAP–240), (CZAP–098, CZAP–057); recordedBy: G. da Silva, D. Costa; individualCount: (1, 3), (2, 5); **Location:** locality: Miramar and Seixas Beaches; verbatimDepth: (1.5 m, 4.0 m), (1.5 m, 4.0 m)

##### Distribution

East Africa, northeast Brazilian coasts (Paraíba and Bahia States) (Amaral et al. 2013, Costa et al. 2017, Read and Fauchald 2020c).

**Distribution in Paraíba**: Miramar Beach (**New record**), Seixas Beach (Costa et al. 2017).

##### Notes

Found inside the rhodoliths.

##### Diagnosis

(Fauchald 1992, Paxton 2009): Prostomium with a median sulcus deep, with four eyes (arranged in a curved line) and five smooth antennae (Fig. 2d). Jaws eulabidognath-type (asymmetrical, posterior parts dentate to forceps-like, short carriers). Formula (maxillae): 1+1, 5+5, 4+0, 3+8 and 1+1. Peristomium with two smooth cirri. Branchiae absent. Notopodia with filiform cirri, larger than ventral ones. Neuropodia with rounded acicular lobes (in anterior segments), conical (median ones) and pointed; thick and tapering ventral cirri (anterior segments), inflated (median ones) and short tubercular (posterior ones); pre and post-chaetal lobes; pectinates and falcigers chaetae, besides dark aciculae single and dark subacicular bidentate hooks.

#### Lysidice ninetta

Audouin & Milne Edwards, 1833

E48C8A11-6458-5AB6-B86E-941ED2319C0C

https://www.marinespecies.org/aphia.php?p=taxdetails&id=130071

##### Materials

- **Type status:**Other material. **Occurrence:** catalogNumber: CZAP–099, CZAP–105; recordedBy: G. da Silva, D. Costa; individualCount: 5, 7; **Location:** locality: Seixas Beach; verbatimDepth: 1.5 m, 4.0 m

##### Distribution

Gulf of Mexico to Caribbean Sea, North Atlantic Ocean (Ireland to Mediterranean Sea), Brazilian coast (Ceará, Rio Grande do Norte, Paraíba, Pernambuco, Alagoas, Sergipe, Bahia (including Abrolhos Archipelago), Rio de Janeiro and São Paulo States), Red Sea and east Australia to New Zealand (Amaral et al. 2013, Costa et al. 2017, Read and Fauchald 2020d).

**Distribution in Paraíba**: Seixas Beach (Costa et al. 2017; and this study).

##### Notes

Found inside the rhodoliths.

##### Diagnosis

(Nonato and Luna 1970, Paxton 2009, Salazar-Vallejo and Carrera-Parra 1997, Uebelacker and Johnson 1984, Zanol et al. 2014): Prostomium rounded without sulcus, with two eyes and three smooth antennae (Fig. 2e). Lack of palps. Jaws eulabidognath-type (asymmetrical, posterior parts dentate to forceps-like, short carriers); mandible curved-like. Formula (maxillae): 1+1, 4+4, 5 to 6+0, 3 to 4+5 to 6 and 1+1. Peristomial cirri and branchiae absent. Notopodia carry digitiform cirri. Neuropodia carry conical cirri smaller than dorsal ones; with limbate, pectinates and falcigers chaetae; dark aciculae single and dark subacicular bidentate hooks.

#### Lysidice unicornis

(Grube, 1840)

D445DB46-7B6A-5D14-9C11-5ED43437095C

https://www.marinespecies.org/aphia.php?p=taxdetails&id=742232

##### Materials

- **Type status:**Other material. **Occurrence:** catalogNumber: (CZAP–219), (CZAP–077); recordedBy: G. da Silva, D. Costa; individualCount: (3), (4); **Location:** locality: Miramar and Seixas Beaches; verbatimDepth: (4.0 m), (4.0 m)

##### Distribution

Atlantic Ocean (North to South), Brazilian coast (Paraíba, São Paulo and Paraná States), Mediterranean Sea, Red Sea, Madagascar and New Zealand (Amaral et al. 2013, Costa et al. 2017, Read and Fauchald 2020e).

**Distribution in Paraíba**: Seixas Beach (Costa et al. 2017), Cabo Branco Beach (DeAssis et al. 2012) and Miramar Beach (**New record**).

##### Notes

Found inside the rhodoliths.

##### Diagnosis

(Paxton 2009, Salazar-Vallejo and Carrera-Parra 1997, Zanol et al. 2014): Prostomium rounded without sulcus, with two eyes and only one smooth antenna (Fig. 2f). Lack of palps. Jaws eulabidognath-type (asymmetrical, posterior parts dentate to forceps-like, short carriers); mandible curved-like. Formula (maxillae): 1+1, 4+4, 4+0, 2+5 and 1+1. Peristomial cirri and branchiae absent. Notopodia with digitiform cirri, larger than ventral cirri. Neuropodia with globular cirri; with limbate, pectinates and falcigers chaetae; yellow aciculae single and yellow subacicular bidentate hooks.

#### Marphysa angelensis

Fauchald, 1970

B605950B-1D0C-5B6C-BA28-6F112DE17601

http://www.marinespecies.org/aphia.php?p=taxdetails&id=329228

##### Materials

- **Type status:**Other material. **Occurrence:** catalogNumber: CZAP–153; recordedBy: G. da Silva, D. Costa; individualCount: 1; **Location:** locality: Seixas Beach; verbatimDepth: 1.5 m

##### Distribution

Gulf of California, Gulf of Mexico and Brazilian coast (Paraíba and São Paulo States) (Amaral et al. 2013, Costa et al. 2017, Read and Fauchald 2020f).

**Distribution in Paraíba**: Seixas Beach (Costa et al. 2017; and this study).

##### Notes

Found inside the rhodoliths.

##### Diagnosis

(Fauchald 1970, Paxton 2009, Salazar-Vallejo and Carrera-Parra 1997): Prostomium with a short anterior incision, with two eyes and five smooth antennae. Jaws eulabidognath-type (asymmetrical, posterior parts dentate to forceps-like, short carriers). Formula (maxillae): 1+1, 3+5, 6+0, 3+8 and 1+1. Peristomial cirri absent. Ramified branchiae (1 to 3 filaments) from chaetiger 9 (Fig. 3a). Anterior notopodial cirri enlarged and posterior ones digitiform, both types longer than ventral ones. Neuropodia with anterior cirri globular, posterior ones conical; limbate, pectinates, falcigers and spinigers chaetae; 1-3 dark aciculae by chaetiger and dark subacicular bidentate hooks.

#### Marphysa regalis

Verrill, 1900

70CB7A14-6A4C-5600-920F-F40A90A8AD75

http://www.marinespecies.org/aphia.php?p=taxdetails&id=329261

##### Materials

- **Type status:**Other material. **Occurrence:** catalogNumber: (CZAP–233, CZAP–245), (CZAP–134, CZAP–188); recordedBy: G. da Silva, D. Costa; individualCount: (3, 7), (1, 1); **Location:** locality: Miramar and Seixas beaches; verbatimDepth: (1.5 m, 4.0 m), (1.5 m, 4.0 m)

##### Distribution

Gulf of Mexico to Caribbean Sea, Bermuda Islands and Brazilian coast (Paraíba, Pernambuco, Alagoas, Bahia, Espírito Santo and Rio de Janeiro States) (Amaral et al. 2013, Costa et al. 2017, Read and Fauchald 2020g).

**Distribution in Paraíba**: Seixas Beach (Costa et al. 2017), pier of the Cabedelo Municipality (DeAssis et al. 2012) and Miramar Beach (**New record**).

##### Notes

Found inside the rhodoliths.

##### Diagnosis

(Molina-Acevedo and Carrera-Parra 2017, Paxton 2009, Salazar-Vallejo and Carrera-Parra 1997): Prostomium divided into two lobes, with two eyes and five smooth antennae (with brown perpendicular bands). Jaws eulabidognath-type (asymmetrical, posterior parts dentate to forceps-like, short carriers). Formula (maxillae): 1+1, 3 to 4+4, 5+0, 5+6 and 1+1. Peristomial cirri absent (Fig. 3b). Ramified branchiae (1 to 4 filaments) from chaetiger 19. Notopodial cirri (longer than neuropodial ones) enlarged in anterior chaetigers, digitiform in posterior chaetigers. Neuropodia carry anterior acicular lobe rounded, posterior ones triangular; anterior globular basally cirri larger than posterior ones; pre- and post-chaetal lobes; limbate, pectinates and falcigers chaetae; 1-3 dark aciculae by chaetiger and dark subacicular unidentate hooks.

#### Marphysa stylobranchiata

Moore, 1909

4928E40C-F8FF-595A-9446-CF027D80FD32

http://www.marinespecies.org/aphia.php?p=taxdetails&id=329267

##### Materials

- **Type status:**Other material. **Occurrence:** catalogNumber: (CZAP–171), (CZAP–069), (CZAP–254, CZAP–275); recordedBy: G. da Silva, D. Costa; individualCount: (2), (3), (1, 1); **Location:** locality: Miramar, Seixas and Maceió Beaches; verbatimDepth: (4.0 m), (1.5 m), (1.5 m, 4.0 m)

##### Distribution

Pacific coast (Monterey Bay) and Brazilian coast (Paraíba, Alagoas, Bahia and Rio de Janeiro States) (Amaral et al. 2013, Costa et al. 2017, Read and Fauchald 2020h).

**Distribution in Paraíba**: Seixas Beach (Costa et al. 2017), Miramar and Maceió Beaches (**New records**).

##### Notes

Found inside the rhodoliths.

##### Diagnosis

(Knox and Green 1972, Nonato and Luna 1970, Paxton 2009): Prostomium with a short anterior incision, with two eyes and five smooth antennae. Jaws eulabidognath-type (asymmetrical, posterior parts dentate to forceps-like, short carriers). Formula (maxillae): 1+1, 4+4 to 5, 4+0, 3 to 4+6 and 1+1. Peristomial cirri absent (Fig. 3c). Branchiae with only one filament from chaetiger 20. Anterior dorsal cirri longer than posterior ones. Neuropodia with cirri smaller than dorsal ones; falcigers chaetae; 1-5 dark aciculae and dark subacicular unidentate hooks.

#### Palola brasiliensis

Zanol, Paiva & Attolini, 2000

C8EA9D02-AE7C-50E2-8860-EEB60D9D0836

http://www.marinespecies.org/aphia.php?p=taxdetails&id=336006

##### Materials

- **Type status:**Other material. **Occurrence:** catalogNumber: CZAP–081; recordedBy: G. da Silva, D. Costa; individualCount: 5; **Location:** locality: Seixas Beach; verbatimDepth: 4.0 m

##### Distribution

Brazilian coast (Paraíba, Bahia, Espírito Santo and Rio de Janeiro States) (Amaral et al. 2013, Costa et al. 2017, Read and Fauchald 2020i).

**Distribution in Paraíba**: Seixas Beach (DeAssis et al. 2012, Costa et al. 2017; and this study).

##### Notes

Found inside the rhodoliths.

##### Diagnosis

(Paxton 2009, Zanol et al. 2000): Prostomium with a short anterior incision, two eyes and five smooth antennae (Fig. 3d). Jaws eulabidognath-type (asymmetrical, posterior parts dentate to forceps-like, short carriers). Formula (maxillae): 1+1, 4+3, 2+0, 2+2 and 1+1. Peristomium with two smooth cirri. Branchiae with single filaments starting on chaetiger 58. Notopodial cirri digitate, anterior longer than other ones. Neuropodia carry anterior cirri digitiform, median ones inflated and posterior ones short and tapering; with limbate and falcigers and dark acicula single. Subacicular hooks absent.

#### Lumbrineridae

Schmarda, 1861

BDE8A933-90CE-5C7E-A1D7-46776BCF3F8A

#### Lysarete brasiliensis

Kinberg, 1865

B414C73C-8FC7-541C-87FC-8D329AB48468

http://www.marinespecies.org/aphia.php?p=taxdetails&id=328978

##### Materials

- **Type status:**Other material. **Occurrence:** catalogNumber: CZAP–052; recordedBy: G. da Silva, D. Costa; individualCount: 2; **Location:** locality: Seixas Beach; verbatimDepth: 1.5 m

##### Distribution

Atlantic Ocean (North to South), Gulf of Mexico, Brazilian coast (Paraíba, São Paulo, Rio de Janeiro, Paraná and Rio Grande do Sul States) and Mexican Pacific coast (Amaral et al. 2013, Costa et al. 2017, Read and Fauchald 2020j).

**Distribution in Paraíba**: Seixas Beach (Costa et al. 2017; and this study).

##### Notes

Found inside the rhodoliths.

**Remarks**: The species was recorded for Seixas Beach as *Lysareteraquelae* Carrera-Parra, 2001 (Costa et al. 2017), but now it is revised and replaced to *Lysaretebrasiliensis*.

##### Diagnosis

(Camargo and Lana 1995, Clemo and Dorgan 2017): Prostomium with four eyes, three antennae and two lips palps (Fig. 3e). Jaws prionognath-type with maxillary parts like scissors with blades (“carriers”). Formula (maxillae): 2+2, 4+4, 4+4, 4+4 and 1+1. Notopodia carry anterior cirri rounded smaller than posterior shaped-foliate ones; and dark acicula single. Neuropodia carry pre- and post-chaetal lobes; limbate chaetae and five aciculae.

#### Oenonidae

Kinberg, 1865

0FEDC24C-1443-5F69-97BA-F723F9262D59

#### Arabella iricolor

(Montagu, 1804)

F7FB990E-5BB2-5F11-BF69-8722D70E7332

http://www.marinespecies.org/aphia.php?p=taxdetails&id=129854

##### Materials

- **Type status:**Other material. **Occurrence:** catalogNumber: CZAP–063; recordedBy: G. da Silva, D. Costa; individualCount: 1; **Location:** locality: Seixas Beach; verbatimDepth: 1.5 m

##### Distribution

Cosmopolitan (Read and Fauchald 2020k).

**Distribution in Paraíba**: Seixas Beach (Costa et al. 2017; and this study).

##### Notes

Found inside the rhodoliths.

**Remarks**: Due to its wide global distribution, this species needs a systematic review.

##### Diagnosis

(Day 1967a, Paxton 2009, Uebelacker and Johnson 1984): Prostomium with four eyes, without appendages (Fig. 3f). Jaws prionognath-type with maxillary parts like scissors with blades (“carriers”). Formula (maxillae): 1+9, 8+14, 7+5, 1+4 and 1+1. Anterior notopodial cirri longer than posterior ones. Neuropodia carry pre- and post-chaetal lobes; serrated winged capillaries and yellow acicula.

#### Onuphidae

Kinberg, 1865

89511AC1-3BD4-5187-88F3-6FDCCA884CEA

#### Kinbergonuphis nonatoi

Lana, 1991

C710F32A-1CB3-5B7C-80B5-F3ED47B1C72C

http://www.marinespecies.org/aphia.php?p=taxdetails&id=328551

##### Materials

- **Type status:**Other material. **Occurrence:** catalogNumber: CZAP–140; recordedBy: G. da Silva, D. Costa; individualCount: 1; **Location:** locality: Seixas Beach; verbatimDepth: 4.0 m

##### Distribution

Brazilian coast (Paraíba, Rio de Janeiro, São Paulo, Paraná and Santa Catarina States) (Amaral et al. 2013, Lana 1991, Read and Fauchald 2020l; and this study).

**Distribution in Paraíba**: Seixas Beach (**New record**). This species represents a new record for the northeast Brazilian coast.

##### Notes

Found inside the rhodoliths.

##### Diagnosis

(Lana 1991): Prostomium with five segmented basal antennae and four eyespots. Jaws eulabidognath-type (asymmetrical, posterior parts dentate to forceps-like, short carriers). Formula (maxillae): 1+1, 9+9, 8+0, 7+12 and 1+1. Peristomium with two cirri (Fig. 4a). Branchiae pectinate (single filaments) from chaetiger 7-8 (other ones with up to 5 strands). Anterior dorsal cirri longer than posterior ones. Neuropodia carry pre- and post-chaetal lobes; limbate and pectinate chaetae, pseudocompound tridentate hooks (1-5 chaetigers), bidentate subacicular hooks (median and posterior chaetigers) and three aciculae.

#### Phyllodocida

Dales, 1962

151A7C78-2923-5528-802A-2FCB37F2D2D6

#### Hesionidae

Grube, 1850

01BA7068-B5E6-59A0-BA6F-BD5A1F29C679

#### Hesione splendida

Lamarck, 1818

B50213DE-D7ED-5BF6-8BCE-6ABE4016FFC8

http://www.marinespecies.org/aphia.php?p=taxdetails&id=130158

##### Materials

- **Type status:**Other material. **Occurrence:** catalogNumber: CZAP–052; recordedBy: G. da Silva, D. Costa; individualCount: 1; **Location:** locality: Seixas Beach; verbatimDepth: 1.5 m

##### Distribution

Red Sea, Mediterranean Sea, Greece, Italy, Atlantic coast of France to Senegal, Cape Verde Archipelago, Brazilian coast (Ceará, Rio Grande do Norte, Paraíba, Pernambuco and Alagoas States), Caribbean Sea, Jamaica, Puerto Rico, Gulf of Mexico, United States of America (Florida), Pacific Ocean, Japan, tropical Indo-West Pacific, Samoa, Tonga and Sri Lanka (Amaral et al. 2013, DeAssis et al. 2012, Costa and Christoffersen 2016, Costa et al. 2008, Costa et al. 2017, Read and Fauchald 2020m).

**Distribution in Paraíba**: Seixas Beach (Costa et al. 2008, Costa et al. 2017, Costa and Christoffersen 2016; and this study).

##### Notes

Found on the rhodoliths surface.

**Remarks**: This species already has been recorded for the northeast Brazilian (Amaral et al. 2013, Costa et al. 2017) and confirmed with morphological description in previous studies (e.g. Costa et al. 2008, Costa and Christoffersen 2016). Notwithstanding, a molecular identification is required and a systematic analysis comparing the genetic distances amongst the species of this genus. For this moment, we continue to consider it as *H.splendida*.

##### Diagnosis

(Costa et al. 2008, Costa and Christoffersen 2016, Imajima 2003, Salazar-Vallejo 2018): Prostomium with four eyes, two papilla-like antennae, facial tubercle, nuchal organs and an incision in posterior end. Anterior proboscis ring smooth, carrying two black points in medium-lateral region and a tubercle in the median-posterior region. First segments with eight pairs of modified cirri (“tentacular cirri”) (Fig. 4b). Parapodia sesquiramous-type (notopodial region reduced to cirri and no chaetae); neuropodia with falcigers and dark aciculae. Neurochaetal blades with two teeth-like processes anteriorly, up to 9 times longer than wide.

#### Oxydromus pugettensis

(Johnson, 1901)

44FA740D-0AFE-5296-8062-B1E30D9BEC52

http://www.marinespecies.org/aphia.php?p=taxdetails&id=710706

##### Materials

- **Type status:**Other material. **Occurrence:** catalogNumber: CZAP–072; recordedBy: G. da Silva, D. Costa; individualCount: 1; **Location:** locality: Seixas Beach; verbatimDepth: 1.5 m

##### Distribution

Northeast Ocean Pacific, United States of America (Washington), Brazilian coast (Paraíba, Rio de Janeiro and São Paulo States) (Amaral et al. 2013, DeAssis et al. 2012, Costa et al. 2017, Read and Fauchald 2020n, Villalobos-Guerrero and Harris 2012).

**Distribution in Paraíba**: Cabedelo and Conde Municipalities (DeAssis et al. 2012), Seixas Beach (Costa et al. 2017; and this study).

##### Notes

Found inside the rhodoliths.

##### Diagnosis

(Uchida 2004): Prostomium with four eyes, three antennae, facial tubercle, nuchal organs, two palps and an incision in posterior end. Anterior proboscis ring ciliated. First segments with six pairs of modified cirri (“tentacular cirri”) (Fig. 4c). Parapodia biramous-type (notopodial and neuropodial lobes with cirri and chaetae), with capillaries, falcigers chaetae and aciculae transparent.

#### Nereididae

Blainville, 1818

F3D95198-192E-5D11-BE9B-DCC7078B7FC4

#### Ceratonereis singularis

Treadwell, 1929

0B0C9428-9502-5553-B656-BDB086655360

http://www.marinespecies.org/aphia.php?p=taxdetails&id=327401

##### Materials

- **Type status:**Other material. **Occurrence:** catalogNumber: CZAP–045; recordedBy: G. da Silva, D. Costa; individualCount: 1; **Location:** locality: Miramar Beach; verbatimDepth: 1.5 m

##### Distribution

Northeast Pacific Ocean (Baja California), Caribbean Sea and Brazilian coast (Maranhão, Paraíba and Alagoas States) (Amaral et al. 2013, Costa et al. 2017, Read and Fauchald 2020o).

**Distribution in Paraíba**: Penha Beach (DeAssis et al. 2012), Seixas Beach (Costa et al. 2017) and Miramar Beach (**New record**).

##### Notes

Found inside the rhodoliths.

##### Diagnosis

(Perkins 1980, Santos and Lana 2003): Prostomium deeply cleft in the anterior region, with four eyes, two antennae (as long as prostomial width) and two palps (size similar to antennae) (Fig. 4d). Proboscis with conical paragnaths (areas I and V, VII and VIII none, area II with 9-15 group long oval ones, area III with 6-10 group triangular ones, area IV with 10-16 group oval ones and area VI with cushion-like lobe) and jaws with 5-6 teeth. Four pairs of modified anterior cirri (“tentacular cirri”). Parapodia with notopodial and neuropodial lobes (with pre- and post-chaetal lobes) of same size, falcigers and spinigers chaetae and dark aciculae.

#### Nereis riisei

Grube, 1857

AD6E575A-1CAA-5519-AC50-633516593233

http://www.marinespecies.org/aphia.php?p=taxdetails&id=329735

##### Materials

- **Type status:**Other material. **Occurrence:** catalogNumber: CZAP–158; recordedBy: G. da Silva, D. Costa; individualCount: 1; **Location:** locality: Seixas Beach; verbatimDepth: 1.5 m

##### Distribution

Gulf of Mexico, Caribbean Sea and Brazilian coast (Pará, Maranhão, Piauí, Ceará, Rio Grande do Norte, Paraíba, Pernambuco, Alagoas, Sergipe, Bahia, Espírito Santo, Rio de Janeiro, São Paulo, Paraná and Santa Catarina States) (Costa et al. 2017, Read and Fauchald 2020p).

**Distribution in Paraíba**: Lucena Beach (DeAssis et al. 2012), Seixas Beach (Costa et al. 2017) and Maceió Beach (**New record**).

##### Notes

Found inside the rhodoliths.

##### Diagnosis

(Amaral et al. 2005, Santos and Lana 2003, Uebelacker and Johnson 1984): Prostomium with four eyes, two antennae, and two palps (Fig. 4e). Proboscis with conical paragnaths (area I with one paragnath, area II 10, area III with 18-20, area IV with 26-30, area V none, area VI with six, area VII and VIII with five) and serrated jaws. Four pairs of modified anterior cirri (“tentacular cirri”). Parapodia with notopodial and neuropodial lobes (with pre- and post-chaetal lobes) of same size, falcigers and spinigers chaetae and dark aciculae.

#### Pseudonereis gallapagensis

Kinberg, 1865

CF0CC20A-08BE-5471-A1C6-23BAC7157A84

##### Materials

- **Type status:**Other material. **Occurrence:** catalogNumber: CZAP–237; recordedBy: G. da Silva, D. Costa; individualCount: 1; **Location:** locality: Miramar Beach; verbatimDepth: 4.0 m

##### Distribution

Pacific Ocean: Galápagos Islands, Hawaii, Baja California to Chile; Brazilian coast (Rio Grande do Norte, Paraíba, Pernambuco, São Paulo and Paraná States); Cape of Good Hope, Madagascar and Red Sea (Costa et al. 2017, Read and Fauchald 2020q).

**Distribution in Paraíba**: Seixas Beach (Costa et al. 2017) and Miramar Beach (**New record**).

##### Notes

Found inside the rhodoliths.

##### Diagnosis

(Dueñas-Ramírez and Quiros-Rodriguez 2012): Prostomium with four eyes, two antennae and two palps (Fig. 4f). Proboscis with paragnaths (area I with two conical ones, area II with three rows of pectinate bars, area III with four pectinate bars, area IV with five pectinate bars, area V with one conical paragnath, area VI with transverse ones and areas VII and VIII with a single row conical ones) and serrated jaws. Four pairs of modified anterior cirri (“tentacular cirri”). Parapodia with notopodial and neuropodial lobes (with pre- and post-chaetal lobes) of same size, falcigers and spinigers chaetae and dark aciculae.

#### Phyllodocidae

Örsted, 1843

986FF988-850E-50FD-844F-FFE3553FF989

#### Phyllodoce schmardaei

Day, 1963

80F08863-772F-556E-AFA1-38AC14531957

https://www.marinespecies.org/aphia.php?p=taxdetails&id=330630

##### Materials

- **Type status:**Other material. **Occurrence:** catalogNumber: CZAP–128; recordedBy: G. da Silva, D. Costa; individualCount: 1; **Location:** locality: Seixas Beach; verbatimDepth: 4.0 m

##### Distribution

South Africa, English Channel, Mediterranean Sea and Brazilian coast (State of Paraíba) (Costa et al. 2017, Read and Fauchald 2020r).

**Distribution in Paraíba**: Seixas Beach (Costa et al. 2017; and this study).

##### Notes

Found inside the rhodoliths.

##### Diagnosis

(Day 1967a): Body green. Prostomium with two eyes, four antennae, nuchal organs and a small posterior-median papilla. Proboscis divided at two parts, proximal one with soft papillae, distal one papillated with six divisions. Four pairs of modified anterior cirri (“tentacular cirri”) (Fig. 5a). Parapodia carry dorsal enlarged foliaceous cirri and neuropodial lobe with spinigers chaetae.

#### Lepidonotus squamatus

(Linnaeus, 1758)

F0AC2AC8-B8B7-5B35-8485-B84437294648

https://www.marinespecies.org/aphia.php?p=taxdetails&id=130801

##### Materials

- **Type status:**Other material. **Occurrence:** catalogNumber: CZAP–040; recordedBy: G. da Silva, D. Costa; individualCount: 4; **Location:** locality: Seixas Beach; verbatimDepth: 1.5 m

##### Distribution

North Atlantic Ocean to Greenland, Mediterranean Sea (Read and Fauchald 2020s) and Brazilian coast (State of Paraíba) (Costa et al. 2017).

**Distribution in Paraíba**: Seixas Beach (Costa et al. 2017; and this study).

##### Notes

Found inside the rhodoliths.

##### Diagnosis

(Imajima and Hartman 1964): Prostomium with four eyes, three antennae and two palps. Dorsum covered by 12 pairs of elytra from anterior to posterior end, with papillae on the surface (first pair elytra with long marginal papillae, other ones reniform) and marginally fringed (Fig. 5b). Notopodial region carries cirri and capillaries chaetae; neuropodial lobes enlarged with falcate chaetae.

#### Syllis guidae

Nogueira & Yunda-Guarin, 2008

7105A85B-FCD7-56E3-A012-69310A1540BD

https://www.marinespecies.org/aphia.php?p=taxdetails&id=760677

##### Materials

- **Type status:**Other material. **Occurrence:** catalogNumber: (CZAP–164, CZAP–276), (CZAP–060); recordedBy: G. da Silva, D. Costa; individualCount: (1, 1), (5); **Location:** locality: Miramar and Seixas bBeaches; verbatimDepth: (1.5 m, 4.0 m), (1.5 m)

##### Distribution

Northeast Brazil (Ceará and Paraíba States) (Amaral et al. 2013, Costa et al. 2017, Read and Fauchald 2020t).

**Distribution in Paraíba**: Seixas Beach (Costa et al. 2017; and this study) and Miramar Beach (**New record**).

##### Notes

Found inside the rhodoliths.

##### Diagnosis

(Nogueira and Yunda-Guarin 2008): Prostomium with four eyes, three antennae, two palps and nuchal organs. All antennae and cirri moniliform-like (similar to pearl necklace). Two pairs of modified anterior cirri (“tentacular cirri”) (Fig. 5c). Digestive tract: pharynx with tooth; a distinct and prominent muscular region of the anterior part (“proventricle”). Parapodia carry falcigers and simple chaetae, one or two aciculae (slender, subdistally enlarged, with short, angled end) per parapodium.

#### Sedentaria

Lamarck, 1818

A446BCB6-3A39-5862-B47F-D5130C2ED6B0

#### Capitellida

Fauchald, 1977

68F22552-06EB-5B72-BA46-6C39BE2A0955

#### Capitellidae

Grube, 1862

69F9F85D-66B3-525F-892D-B823BC999D81

#### Neopseudocapitella brasiliensis

Rullier & Amoureux, 1979

AABCA912-F630-5A4C-AF10-3FD39F716D84

https://www.marinespecies.org/aphia.php?p=taxdetails&id=129893

##### Materials

- **Type status:**Other material. **Occurrence:** catalogNumber: CZAP–161; recordedBy: G. da Silva, D. Costa; individualCount: 1; **Location:** locality: Seixas Beach; verbatimDepth: 1.5 m

##### Distribution

Brazilian coast (Paraíba, Sergipe and Bahia States), Iberian Peninsula, and Mediterranean Sea (Amaral et al. 2013, Costa et al. 2017, Read and Fauchald 2020u).

**Distribution in Paraíba**: Seixas Beach (Costa et al. 2017; and this study).

##### Notes

Found inside the rhodoliths.

##### Diagnosis

(Amaral 1980, Rullier and Amoureux 1979): Prostomium conical-shaped with two eyespots and nuchal organs, without antennae (Fig. 5d). Proboscis enlarged and voluminous. Parapodial cirri absent. Capillaries chaetae start from chaetiger one, hooks from chaetiger 12.

#### Orbiniida

Fauchald, 1977

0A0210AF-FADA-5A2A-940F-F433CAD21D00

#### Orbiniidae

Hartman, 1942

89B27872-6EC3-58C0-AE34-4A94B9E02084

#### Naineris setosa

(Verrill, 1900)

EDF199E1-2709-5A00-9C6B-A67C3336AD20

https://www.marinespecies.org/aphia.php?p=taxdetails&id=334062

##### Materials

- **Type status:**Other material. **Occurrence:** catalogNumber: CZAP–048, CZAP–043; recordedBy: G. da Silva, D. Costa; individualCount: 10, 1; **Location:** locality: Seixas Beach; verbatimDepth: 1.5 m, 4.0 m

##### Distribution

Eastern Pacific: Mexico to Costa Rica; Atlantic Ocean: Gulf of Mexico, Caribbean Sea, Bermuda and Brazilian coast (Paraíba, Rio de Janeiro, São Paulo and Paraná States) (Amaral et al. 2013, Costa et al. 2017, Read and Fauchald 2020v).

**Distribution in Paraíba**: Seixas Beach (Costa et al. 2017; and this study).

##### Notes

Found inside the rhodoliths.

##### Diagnosis

(Khedhri et al. 2014): Prostomium T-shaped, with few eyespots grouped in two ‘comma groups’ forming Y-shape. Eversible pharynx (proboscis) enlarged sac-like. Branchiae from chaetiger 6, basally broader and tapering to pointed end (Fig. 5e). Paired sensorial organs from chaetiger 8 in upper zone of branchiae. Dorsal crests in dorsum, mainly in abdominal region. Parapodia with neuropodial lobes shorter than notopodial ones; notopodia carry capillaries and furcate chaetae (last ones only in abdomen region); neuropodia with capillaries and uncini (last one in posterior segments).

#### Sabellida

Levinsen, 1883

ED78E96A-4B22-5917-A1B0-62B9F861564F

#### Sabellariidae

Johnston, 1865

0E1B296D-53F2-5610-BF13-3B10C081DA12

#### Phragmatopoma caudata

Krøyer in Mörch, 1863

7703F839-4F41-530E-B291-47B730BF7598

https://www.marinespecies.org/aphia.php?p=taxdetails&id=330550

##### Materials

- **Type status:**Other material. **Occurrence:** catalogNumber: (CZAP–039), (CZAP–280); recordedBy: G. da Silva, D. Costa; individualCount: (1), (6); **Location:** locality: Seixas and Maceió Beaches; verbatimDepth: (1.5 m), (4.0 m)

##### Distribution

Gulf of Mexico, West Indies, Brazilian coast (Maranhão, Piauí, Ceará, Rio Grande do Norte, Paraíba, Pernambuco, Alagoas, Sergipe, Bahia, Espírito Santo, Rio de Janeiro, São Paulo, Paraná and Santa Catarina States) and north of the South China Sea (Pratas Islands) (Amaral et al. 2013, Costa et al. 2017, Read and Fauchald 2020w).

**Distribution in Paraíba**: Infralittoral region from Cabedelo Municipality (after coral reefs zones known as “Areia Vermelha” and “Barretas”), Bessa Beach (DeAssis et al. 2012), Seixas Beach (Costa et al. 2017; and this study) and Maceió Beach (**New record**).

##### Notes

Found inside the rhodoliths.

##### Diagnosis

(Capa et al. 2012): Anterior end with an operculum longer than wide, with merged lobes in shallow mid-ventral indentation. Distal disc flat and perpendicular to longitudinal axis, with opercular papillae around it (Fig. 5f). Outer paleae arranged in curved line. Inner operculum paleae like two concentric rows, with paleae geniculate carrying convex blades. Pectinate tentacular filaments arranged in series of rows. Palps similar in length to operculum. Neuropodia of chaetiger 1 carry one conical cirri. Chaetiger 2 carry four triangular-shaped lobes. Thorax with branchiae. Three parathoracic segments, with lanceolate and capillaries chaetae.

#### Sabellidae

Latreille, 1825

3FC37337-E38A-5B09-A2B5-C1984D8E83BF

#### Branchiomma nigromaculatum

(Baird, 1865)

B5676943-08B0-501B-B558-FE560841B0EA

https://www.marinespecies.org/aphia.php?p=taxdetails&id=209930

##### Materials

- **Type status:**Other material. **Occurrence:** catalogNumber: CZAP–058; recordedBy: G. da Silva, D. Costa; individualCount: 2; **Location:** locality: Seixas; verbatimDepth: 1.5 m

##### Distribution

Gulf of Mexico, West Indies, Brazilian coast (Paraíba, Pernambuco, Alagoas, Sergipe, Bahia, Espírito Santo, Rio de Janeiro and São Paulo States), Angola (Luanda) and East Africa (Amaral et al. 2013, Costa et al. 2017, Read and Fauchald 2020x).

**Distribution in Paraíba**: Cabo Branco Beach (DeAssis et al. 2012), Seixas Beach (Costa et al. 2017; and this study).

##### Notes

Found inside the rhodoliths.

##### Diagnosis

(Tovar-Hernández and Knight-Jones 2006): Body with dark spots on the dorsal and ventral surfaces; interramal dark spots. Radiolar crown united at the base by short web or membrane (Fig. 6a). A total of 46 pairs of radioles, with stylodes and dark brown bands alternating with bands of white and orange; 5-6 ventralmost radioles on each side without stylodes, arising from enrolled parts of crown basis; rachis with segmented appearance. Thoracic unciniger (‘tori’) carry avicular uncini. Presence of collar chaetae like compact fascicles.

#### Hypsicomus capensis

Day, 1961

6864FADC-CFE7-5E18-9E2E-9B48BF58C183

https://www.marinespecies.org/aphia.php?p=taxdetails&id=328486

##### Materials

- **Type status:**Other material. **Occurrence:** catalogNumber: CZAP–133; recordedBy: G. da Silva, D. Costa; individualCount: 2; **Location:** locality: Seixas Beach; verbatimDepth: 1.5 m

##### Distribution

Brazilian coast (State of Paraíba), South Africa (Read and Fauchald 2020y; and this study).

**Distribution in Paraíba**: Seixas Beach (**New record**). This species represents a new record for the West Atlantic Ocean coast.

##### Notes

Found inside the rhodoliths.

##### Diagnosis

(Day 1967b): Anterior end with branchial lobes supported by stalk; each lobe carries 12 radioles. These ones with about 20 eyespots. Collar divided at two regions lobe-like (Fig. 6b). Collar chaetae are capillaries arranged in a line. Chaetigers 2-8 carry notochaetae capillaries and paleae with rounded blades ending in pointed tips; and neurochaetae like row of pick-axe chaetae with transparent tapered blades and a row of avicular uncini. Abdominal notochaetae are avicular uncini similar to the thoracic ones and the neurochaetae are capillaries.

#### Terebellida

Rouse & Fauchald, 1997

74794221-92DA-5E89-826B-9D268991DCD8

#### Cirratulidae

Ryckholt, 1851

48B106FA-A85E-58EA-A108-61D69D0F2B5C

#### Cirratulus africanus

Gravier, 1906

93AA9A22-79A0-55E7-8802-6F2A5F06A151

https://www.marinespecies.org/aphia.php?p=taxdetails&id=209867

##### Materials

- **Type status:**Other material. **Occurrence:** catalogNumber: CZAP–083; recordedBy: G. da Silva, D. Costa; individualCount: 2; **Location:** locality: Seixas Beach; verbatimDepth: 4.0 m

##### Distribution

Brazilian coast (Paraíba, Bahia, Rio de Janeiro and São Paulo States), Mozambique and Red Sea (Amaral et al. 2013, Costa et al. 2017, Read and Fauchald 2020z).

**Distribution in Paraíba**: Seixas Beach (Costa et al. 2017; and this study).

##### Notes

Found inside the rhodoliths.

##### Diagnosis

(Day 1967b): Prostomium pointed, without eyes. 3-4 tentacular filaments in anterior segments (Fig. 6c). Branchiae from chaetiger 3 to beginning of the posterior end, arising close above the notochaetae. Parapodia with capillary chaetae in notopodial and neuropodial lobes. There are also chaetae similar to intermediate between capillaries and acicular hooks and normal sigmoid hooks about the middle of the body.

#### Cirriformia capensis

(Schmarda, 1861)

BD8F25C0-0216-521D-A7B9-8153D2C169ED

https://www.marinespecies.org/aphia.php?p=taxdetails&id=332691

##### Materials

- **Type status:**Other material. **Occurrence:** catalogNumber: CZAP–229; recordedBy: G. da Silva, D. Costa; individualCount: 1; **Location:** locality: Miramar Beach; verbatimDepth: 4.0 m

##### Distribution

Gulf of Mexico, Brazilian coast (State of Paraíba) and South Africa (Costa et al. 2017, Read and Fauchald 2020).

**Distribution in Paraíba**: Seixas Beach (Costa et al. 2017), Miramar Beach (**New record**).

##### Notes

Found inside the rhodoliths.

##### Diagnosis

(Day 1967b): Anterior end of prostomium rounded, without eyes. Anterior chaetigers carry numerous tentacular cirri (Fig. 6d). Branchiae from chaetiger 1 to the posterior end. Robust single filaments and, in the middle of the body, they rise further above the notochaetae. Parapodia with capillary chaetae in notopodial and neuropodial lobes. Sigmoid hooks appear about chaetiger 12.

#### Timarete punctata

(Grube, 1859)

868EAA38-18B7-5AC1-A0D9-3E3EC1EEC900

https://www.marinespecies.org/aphia.php?p=taxdetails&id=761959

##### Materials

- **Type status:**Other material. **Occurrence:** catalogNumber: (CZAP–184), (CZAP–066, CZAP–091); recordedBy: G. da Silva, D. Costa; individualCount: (1), (7, 1); **Location:** locality: Miramar and Seixas Beaches; verbatimDepth: (4.0 m), (1.5 m, 4.0 m)

##### Distribution

Mexico, Gulf of Mexico, West Indies and Brazilian coast (Maranhão, Piauí, Ceará, Rio Grande do Norte, Paraíba, Pernambuco, Alagoas, Sergipe, Bahia, Espírito Santo, Rio de Janeiro and São Paulo States) (Amaral et al. 2013, Costa et al. 2017, Read and Fauchald 2020).

**Distribution in Paraíba**: Mataraca, Baía da Traição, Rio Tinto, João Pessoa (including Seixas Beach) and Conde Municipalities (DeAssis et al. 2012, Costa et al. 2017; and this study) and Miramar Beach (**New record**).

##### Notes

Found inside the rhodoliths.

##### Diagnosis

(Çinar 2007): Robust prostomium bluntly pointed in anterior end, without eyes. Peristomium with two segmentations. Tentacular filaments from chaetigers 3 and 4, forming two evident groups, each with five filaments (Fig. 6e). Branchiae from chaetiger 1 to posterior segments. Parapodia with capillary chaetae in notopodial and neuropodial lobes. Acicular spines (slightly sigmoid, with truncated tips) from notochaetae 8 and neurochaetae 6; pale brown.

#### Flabelligeridae

De Saint-Joseph, 1894

A51D2A2E-348D-5E77-ADC0-8394076977CA

#### Pherusa scutigera

(Ehlers, 1887)

8D26B61A-351E-5A32-B3DE-588F2981CB7C

https://www.marinespecies.org/aphia.php?p=taxdetails&id=334492

##### Materials

- **Type status:**Other material. **Occurrence:** catalogNumber: CZAP–097; recordedBy: G. da Silva, D. Costa; individualCount: 1; **Location:** locality: Seixas Beach; verbatimDepth: 1.5 m

##### Distribution

Caribbean Sea and Brazilian coast (Paraíba, Sergipe, Rio de Janeiro, São Paulo and Rio Grande do Sul States) (Amaral et al. 2013, Costa et al. 2017, Read and Fauchald 2020).

**Distribution in Paraíba**: Seixas Beach (Costa et al. 2017); and this study.

##### Notes

Found inside the rhodoliths.

##### Diagnosis

(Nonato and Luna 1970): Body covered by papillae. Chaetigers 1-3 covered by thin layer of sand on the dorsal surface. Anterior region prolonged by a translucent membranous tube. Chaetae three first iridescent, forming a cephalic cage (Fig. 6f). A waistline marks the transition between the anterior segments and the posterior ones. Chaetigers 1-5 with capillaries chaetae; following segments with ventral aciculae chaetae.

#### Terebellidae

Johnston, 1846

C7A862CC-646B-5F02-9666-3AED307EB1D3

#### Terebella plagiostoma

Schmarda, 1861

8AF3FEB0-6EB8-52A3-815B-0400618A708A

https://www.marinespecies.org/aphia.php?p=taxdetails&id=340127

##### Materials

- **Type status:**Other material. **Occurrence:** catalogNumber: CZAP–221, CZAP–273; recordedBy: G. da Silva, D. Costa; individualCount: 2, 1; **Location:** locality: Miramar Beach; verbatimDepth: 1.5 m, 4.0 m

##### Distribution

Brazilian coast (Paraíba and Rio de Janeiro States) (Amaral et al. 2013, Costa et al. 2017, Read and Fauchald 2020).

**Distribution in Paraíba**: Seixas Beach (Costa et al. 2017) and Miramar Beach (**New record**).

##### Notes

Found inside the rhodoliths.

##### Diagnosis

(Hutchings and Smith 1997, Rozbaczylo et al. 2006): Prostomium carry tentacular lobe horseshoe-shaped with numerous grooved tentacles; eyespots arranged in two or three rows on the posterior margin of the tentacular lobe. Three pairs of branchiae with spiral filaments, on segments 2 to 4. (Fig. 7a). Segment 6 with three pairs branchiae with spiral filaments. Notochaetae capillaries-like arranged in two rows. Uncini from chaetiger 3; uncinigers (‘tori’) from segment 2.

#### Terebella pterochaeta

Schmarda, 1861

49FFEAAD-F487-519A-B4C8-0FEA251A8E95

https://www.marinespecies.org/aphia.php?p=taxdetails&id=209923

##### Materials

- **Type status:**Other material. **Occurrence:** catalogNumber: CZAP–073; recordedBy: G. da Silva, D. Costa; individualCount: 2; **Location:** locality: Seixas Beach; verbatimDepth: 1.5 m

##### Distribution

Caribbean Sea, Colombia, Brazilian coast (Paraíba and São Paulo States), South Africa, Mozambique and Red Sea (Amaral et al. 2013, Costa et al. 2017, Read and Fauchald 2020).

**Distribution in Paraíba**: Seixas Beach (Costa et al. 2017; and this study).

##### Notes

Found inside the rhodoliths.

##### Diagnosis

(Day 1967b): Prostomium with delineated dorsal and ventral lips. Two pairs of branchiae at anterior end (Fig. 7b). About 16 ventral pads followed by a narrow streak of glandular tissue in a ventral groove along the abdomen. Uncini on ventral tori originate from ventral ridges on the abdomen, with 3-4 teeth. Notochaetae: anterior ones with winged shafts and denticulate tips which become proportionately larger on posterior segments until they form most of the blade.

#### Echiura

Sedgwick, 1898

9959B7BF-58D0-5CF6-A4FC-8DC58728CAB6

#### Echiuroidea

Bock, 1942

8E6E00B9-F707-5AE4-AEF5-1F96FD9A2CFB

#### Echiuridae

Quatrefages, 1847

8BF615BB-9216-515B-83F6-4484A03057D1

#### Echiurus echiurus

(Pallas, 1766)

CA5A3926-1F6A-54E6-9D69-37BF7848A9F2

https://www.marinespecies.org/aphia.php?p=taxdetails&id=110377

##### Materials

- **Type status:**Other material. **Occurrence:** catalogNumber: (CZAP–114), (CZAP–213); recordedBy: G. da Silva, D. Costa; individualCount: (1), (2); **Location:** locality: Seixas and Maceió Beaches; verbatimDepth: (4.0 m), (4.0 m)

##### Distribution

West and east from North and Central Atlantic Ocean, Brazilian coast (State of Paraíba), Arctic Ocean and New Zealand (WoRMS 2020a; this study).

**Distribution in Paraíba**: Seixas and Maceió Beaches (**New records**). This species represents a new record from the Brazilian coast.

##### Notes

Found inside the rhodoliths.

##### Diagnosis

(Biseswar 1997): Proboscis spoon-shaped with brown streaks. Head with two ventral golden chaetae (Fig. 7c). Body with outer layer of integument in posterior half of trunk separated from inner layers, but still attached to trunk. Conical papillae, projecting from surface of integument, arranged in concentric rings around trunk. To the naked eye, papillae appear as spherical, transparent spots. Rows of larger papillae alternate with three or four rows of uniform smaller papillae. Two rows of anal chaetae around posterior end.

#### Sipuncula

Stephen, 1964

A15F2CA5-BEE0-5DA4-AC03-0B3984417D2C

#### Phascolosomatidea

Hayward & Ryland, 1990

87C3EFA4-938E-57BF-9E14-8CBCBE50A188

#### Aspidosiphonida

995F914C-FA36-501A-AE06-4C35EA8B7C1D

#### Aspidosiphonidae

Baird, 1868

745DE8EF-3DD2-5967-8B2B-FA765EB1F73C

#### Aspidosiphon (Paraspidosiphon) steenstrupii

Diesing, 1859

DCD023AB-127F-5776-94F1-F9B63101A185

http://www.marinespecies.org/aphia.php?p=taxdetails&id=136038

##### Materials

- **Type status:**Other material. **Occurrence:** catalogNumber: CZAP–111, CZAP–112; recordedBy: G. da Silva, D. Costa; individualCount: 10, 1; **Location:** locality: Seixas Beach; verbatimDepth: 1.5 m, 4.0 m

##### Distribution

Gulf of Mexico and Tropical Central Atlantic Ocean (Saiz-Salinas 2020a).

**Distribution in Paraíba**: Seixas Beach (**New record**).

##### Notes

Found inside the rhodoliths.

##### Diagnosis

(Hylleberg 1994): Body semi-transparent in the middle region, darker anteriorly and posteriorly (Fig. 7d). Anal shield covered with dark chalky points. Margin caudal shield with irregular ridges. Anterior end known as “introvert” similar in size to body; with rows of double-pointed hooks anteriorly; spines posteriorly. Longitudinal musculature in separate bands.

#### Phascolosomatida

Hayward & Ryland, 1990

38E78550-3B85-596E-8AFC-97853712E88E

#### Phascolosomatidae

Stephen & Edmonds, 1972

4EC2F12B-DA8F-54D9-B71F-7960BB7686A9

#### Phascolosoma (Phascolosoma) nigrescens

(Keferstein, 1865)

E07C176A-2668-50F5-A960-F9317C787E02

http://www.marinespecies.org/aphia.php?p=taxdetails&id=220538

##### Materials

- **Type status:**Other material. **Occurrence:** catalogNumber: (CZAP–174, CZAP–182), (CZAP–150); recordedBy: G. da Silva, D. Costa; individualCount: (1, 1), (1); **Location:** locality: Miramar and Seixas Beaches; verbatimDepth: (1.5 m, 4.0 m), (4.0 m)

##### Distribution

Circumtropical (Saiz-Salinas 2020b).

**Distribution in Paraíba**: Miramar and Seixas Beaches (**New records**).

##### Notes

Found inside the rhodoliths.

##### Diagnosis

(Hylleberg 1994): Body marbled with brown flecks and bands. Anterior end known as “introvert” longer than the body, carrying numerous rows of hooks and the dorsal side crossed by brownish bands intermingled with lighter ones (Fig. 7e). Hooks with a distinct streak (triangle and internal clear steak not divided), prominent swelling of proximal crescent. Above 20 tentacles.

#### Golfingiida

Hayward & Ryland, 1990

8AF08BE4-6D67-5D91-9D6B-FB4B1D9ABA58

#### Sipunculidae

Rafinesque, 1814

D4B71543-D6B2-54D7-9328-CC3C054615C9

#### Sipunculus (Sipunculus) phalloides

Pallas, 1774

35D31CD4-A50B-5455-B63A-7F72CAAF21D5

http://www.marinespecies.org/aphia.php?p=taxdetails&id=136085

##### Materials

- **Type status:**Other material. **Occurrence:** catalogNumber: CZAP–126; recordedBy: G. da Silva, D. Costa; individualCount: 6; **Location:** locality: Seixas Beach; verbatimDepth: 4.0 m

##### Distribution

Tropical Central Atlantic Ocean (Saiz-Salinas 2020c).

**Distribution in Paraíba**: Seixas Beach (**New record**).

##### Notes

Found inside the rhodoliths.

##### Diagnosis

(Cutler and Cutler 1985): Sipunculid with longitudinal muscle bands (LMBs) and the nephridiopores open between LMBs 4 and 5, 5 and 6, 6 and 7 or 7 and 8. Nephridia less than 25% of trunk length and unattached. They open 5-10% of the trunk length anterior to the anus. The ventral retractors originate on LMB 1 or 2 and extend over 2-6 bands, while the dorsal ones start on LMB 12-16 and spread over 2-6 bands. The LMBs do not subdivide in the glans region. Spindle muscle weakly developed (Fig. 7f).

#### Mollusca

Linnaeus, 1758

556DA460-7D01-5595-B4EE-A8D1EC9C7D6A

#### Bivalvia

Linnaeus, 1758

0DB0B86C-BA68-56BE-B3D0-EC58EC1D9028

#### Autobranchia

Grobben, 1894

83457DF9-2C5D-5CA9-9989-A2A895F5F12D

#### Venerida

Gray, 1854

9B95FC4A-3B6B-5E90-9558-B6B46ED802FB

#### Mactridae

Lamarck, 1809

44A0DB26-E8D7-50EA-8BF2-4513146169C5

#### Mulinia cleryana

(d'Orbigny, 1846)

D02994FE-DB0A-5101-8894-61E2FAB44F20

http://www.marinespecies.org/aphia.php?p=taxdetails&id=505740

##### Materials

- **Type status:**Other material. **Occurrence:** catalogNumber: (CZAP–231), (CZAP–166), (CZAP–214); recordedBy: G. da Silva, D. Costa; individualCount: (1), (1), (3); **Location:** locality: Miramar, Seixas and Maceió Beaches; verbatimDepth: (4.0 m), (1.5 m), (1.5 m)

##### Distribution

Gulf of Mexico to all the Brazilian coast (Gernet et al. 2018, MolluscaBase 2020a, Signorelli 2019).

**Distribution in Paraíba**: Paraíba River Estuary (Lima et al. 2017), Miramar, Seixas and Maceió Beaches (**New records**).

##### Notes

Found inside the rhodoliths.

##### Diagnosis

(Signorelli 2019): Shells trigonal, inflated, umbos placed about half shell length, inflated and prosogyrate; external surface smooth; postero-dorsal area defined by a distinct keel-like carina; anterior and posterior ends low and well defined (Fig. 8a). Ventral margin sinuous; right hinge with two anterior and two posterior lateral teeth, being the ventral more elongated, two divergent and unfused cardinal teeth; left hinge with the usual V-shaped cardinal tooth, flanked by an accessory lamella, one anterior and one posterior lateral tooth complete the hinge; anterior adductor muscle scars semi-elliptical, posterior oval; pallial sinus low and V-shaped.

#### Ungulinidae

Gray, 1854

9529B0A1-A3F0-5BAD-B07F-F9568BC68986

#### Phlyctiderma semiasperum

(Philippi, 1836)

36140045-35C3-506C-82D7-52E1528CC0FB

http://www.marinespecies.org/aphia.php?p=taxdetails&id=420810

##### Materials

- **Type status:**Other material. **Occurrence:** catalogNumber: CZAP–294; recordedBy: G. da Silva, D. Costa; individualCount: 1; **Location:** locality: Maceió Beach; verbatimDepth: 4.0 m

##### Distribution

Gulf of Mexico and Brazilian coast (State of Paraíba) (MolluscaBase 2020b; this study).

**Distribution in Paraíba**: Maceió Beach (**New record**). This species represents a new record from the South Atlantic.

##### Notes

Found inside the rhodoliths.

##### Diagnosis

(Romera 2012): Subtriangular valves, later pointed, white in colour; covered by thin and translucent periostracum; sideways inflated (Fig. 8b). External, opisdotelic and parvincular ligaments. Tall and wide. Externally ornamented by small pustules. Internally white in colour. Similar muscle impressions. Whole pallial line without sinus. Heterodont hinge, with two cardinal teeth. Long and narrow nymph.

#### Mytilida

Férussac, 1822

60787C22-F141-56BB-8B4C-EF806EAF0633

#### Mytilidae

Rafinesque, 1815

92FB4325-D77D-5385-AFC2-212D028B8E14

#### Brachidontes exustus

(Linnaeus, 1758)

CCCA59E4-DBEF-52D7-945D-9E993ED9EE3B

http://www.marinespecies.org/aphia.php?p=taxdetails&id=397026

##### Materials

- **Type status:**Other material. **Occurrence:** catalogNumber: CZAP–148; recordedBy: G. da Silva, D. Costa; individualCount: 1; **Location:** locality: Seixas Beach; verbatimDepth: 1.5 m

##### Distribution

Gulf of Mexico, Celtic Sea and Brazilian coast (State of Paraíba) (Lima et al. 2017, MolluscaBase 2020c).

**Distribution in Paraíba**: Paraíba River Estuary (Lima et al. 2017) and Seixas Beach (**New record**).

##### Notes

Found inside the rhodoliths.

##### Diagnosis

(Smithsonian-Marine-Station-at-Fort-Pierce 2020): Shell dark-brown colour. Fan-shaped shell with fine divercating radial ribs. The ribbed surface of the shell is most evident at the outer edges. Umbones situated in the anterior end (Fig. 8c). The interior has purple-brown blotches with one to four small purplish dysodont hinge teeth.

#### Mytella strigata

(Hanley, 1843)

075BFD5B-9EEF-5293-8562-050FC4734CD9

http://www.marinespecies.org/aphia.php?p=taxdetails&id=1458663

##### Materials

- **Type status:**Other material. **Occurrence:** catalogNumber: CZAP–220; recordedBy: G. da Silva, D. Costa; individualCount: 1; **Location:** locality: Maceió Beach; verbatimDepth: 1.5 m

##### Distribution

United States of America and Brazilian coast (Paraíba and Rio de Janeiro States) (Lima et al. 2017, MolluscaBase 2020d).

**Distribution in Paraíba**: Paraíba River Estuary (Lima et al. 2017) and Maceió Beach (**New record**).

##### Notes

Found inside the rhodoliths.

##### Diagnosis

(Mediodia et al. 2017): With a smooth and shiny symmetrical shell and has predominantly dark brown to black colour with wavy dark pattern. Sculpture of fine concentric semi-circular rings (Fig. 8d). It has two similar-shaped valves joined by a hinge without teeth at the anterior portion. It has two muscle scars, the large posterior adductor muscle scar and the greatly reduced anterior adductor muscle. The byssal and pedal retractor muscle scar is located below the adductor muscle forming a thick straight line moving towards the middle portion of the shell. The pallial line was seen as a curved line towards the adductor scar.

#### Ostreida

Férussac, 1822

26333B0A-E50E-5E13-8C10-6D9E7761BFC6

#### Ostreidae

Rafinesque, 1815

ACE35D24-85D8-5962-896C-7C26157143FC

#### Crassostrea brasiliana

(Lamarck, 1819)

1AF66F88-6D14-59C4-8B6E-1DC840FA7BA7

http://www.marinespecies.org/aphia.php?p=taxdetails&id=506705

##### Materials

- **Type status:**Other material. **Occurrence:** catalogNumber: (CZAP–151, CZAP–123), (CZAP–293, CZAP–285); recordedBy: G. da Silva, D. Costa; individualCount: (4, 3), (2, 3); **Location:** locality: Seixas and Maceió Beaches; verbatimDepth: (1.5 m, 4.0 m), (1.5 m, 4.0 m)

##### Distribution

Brazilian coast (Rio Grande do Norte, Paraíba, Alagoas, Espírito Santo, Rio de Janeiro, São Paulo, Paraná and Santa Catarina States) (Amaral and Simone 2014, Gernet et al. 2018, Lima et al. 2017, MolluscaBase 2020e).

**Distribution in Paraíba**: Paraíba River Estuary (Lima et al. 2017) and Seixas and Maceió Beaches (**New records**).

##### Notes

Found inside the rhodoliths.

##### Diagnosis

(Amaral and Simone 2014): Shell shape cupped or oval; right valve slightly operculum-shaped; left valve, fixed in substrate, larger than right valve (Fig. 8e). Muscle impression is purple and adductor muscle, oval central in posterior region. Adductor muscle postero-dorsal located, occupying 1/5 of total size of animal; hood present and fully filled by palps and gonads; colour of mantle edge brown. Accessory heart of three branches of similar length, starting from common centre. Palps with margin superior free.

#### Gastropoda

Cuvier, 1795

CB8C8CAE-CDB9-5249-B929-99715CAEC304

#### Caenogastropoda

Cox, 1960

B365A098-F6EE-5056-9741-5341BC2DFCC5

#### Neogastropoda

Wenz, 1938

253E782E-E52E-53BA-AB1B-9D13DA0AC647

#### Columbellidae

Swainson, 1840

210E7938-AEC5-5367-96D7-58026356ACD1

#### Parvanachis obesa

(Adams, 1845)

A5F8DC29-4262-5474-A49C-56AA39F5B2EE

https://www.marinespecies.org/aphia.php?p=taxdetails&id=160440

##### Materials

- **Type status:**Other material. **Occurrence:** catalogNumber: CZAP–139; recordedBy: G. da Silva, D. Costa; individualCount: 1; **Location:** locality: Seixas Beach; verbatimDepth: 1.5 m

##### Distribution

East Pacific Ocean: Mexico to Colombia and West Atlantic Ocean (MolluscaBase 2020f).

**Distribution in Paraíba**: Paraíba River Estuary (Lima et al. 2017) and Seixas Beach (**New record**).

##### Notes

Found on the rhodoliths surface.

##### Diagnosis

(Muniz 2015): Gastropod with oval-shaped shell, slightly spiral convex sculptures with axial ventricular ribs, ending towards the base. Spiral sculpture found between axial ribs over basal area of shell (Fig. 8f). Oblique, denticulated opening on the inner surface of the outer lip, straight columella, anal notch present.

#### Polyplacophora

Gray, 1821

BCBEF94D-89D4-555F-82C7-38934A4D9EEA

#### Neoloricata

Bergenhayn, 1955

40E6652C-7DC5-572D-ACA1-1B87E97BDE45

#### Chitonida

Thiele, 1909

AFDD3CF9-20DB-5801-B0BE-05932D0A81EA

#### Acanthochitonidae

Pilsbry, 1893

4CA2636C-5D75-5BF0-A811-486523448B3C

#### Acanthochitona terezae

Guerra Júnior, 1983

58614811-D4B9-592C-89CA-1C173F56F567

https://www.marinespecies.org/aphia.php?p=taxdetails&id=386520

##### Materials

- **Type status:**Other material. **Occurrence:** catalogNumber: CZAP–142; recordedBy: G. da Silva, D. Costa; individualCount: 1; **Location:** locality: Seixas Beach; verbatimDepth: 1.5 m

##### Distribution

Brazilian coast: Paraíba, Pernambuco (Fernando de Noronha Archipelago), Bahia and Espírito Santo States (Trindad Islands, MD55 station) (Jardim et al. 2017, MolluscaBase 2020g; this study).

**Distribution in Paraíba**: Seixas Beach (**New record**). This species represents a new record from the State of Paraíba coast.

##### Notes

Found on the rhodoliths surface.

##### Diagnosis

(Jardim et al. 2017): Mollusc with many plates on surface. Tegument with many white spots mainly on apical region. Girdle white with transverse orange bands. Intermediate valves trapezoidal to oblong in outline, subcarinate, weakly beaked (Fig. 9a). Pustules on latero-pleural area round to oval, randomly arranged; each pustule convex, bearing 4–7 pores on superior to median surface. Tail valve with prominent, submedian mucro; postmucronal area concave. Dorsal side of girdle covered with minute elongated spicules; spicule height about 8–9 times as long as wide, sculptured by longitudinal parallel fissures. Sutural tufts with elongated spicules and sculptured by longitudinal fissures.

#### Arthropoda

Von Siebold, 1848

6644A0D1-98CF-5D88-BEA2-53D9A9C9BC8B

#### Crustacea

Brünnich, 1772

DF89D56D-B7D8-5858-BEC4-8C749D107708

#### Malacostraca

Latreille, 1802

E8BEC128-C323-599C-B0BF-493DF2682FB2

#### Eumalacostraca

Grobben, 1892

38DDCF4F-CA69-587A-B7B6-833A0E694C8A

#### Amphipoda

Latreille, 1816

3D6BBEDE-6E62-54AE-83DE-2A74DF248ACA

#### Maeridae

Krapp-Schickel, 2008

0AB019D1-790B-567E-9228-1D18DC33EDCA

#### Elasmopus brasiliensis

(Dana, 1853)

5B056E7D-49A1-5BD1-96C5-4DE3226A10F4

http://www.marinespecies.org/aphia.php?p=taxdetails&id=102801

##### Materials

- **Type status:**Other material. **Occurrence:** catalogNumber: (CZAP–167, CZAP–109), (CZAP–272); recordedBy: G. da Silva, D. Costa; individualCount: (20, 2), (1); **Location:** locality: Seixas and Maceió Beaches; verbatimDepth: (1.5 m, 4.0 m), (4.0 m)

##### Distribution

Atlantic Ocean: Caribbean Sea, Brazilian coast (Paraíba, Pernambuco, Bahia, Espírito Santo, Rio de Janeiro and São Paulo States); Mediterranean Sea and Red Sea (Serejo and Siqueira 2018, Horton et al. 2020a).

**Distribution in Paraíba**: Seixas and Maceió Beaches (**New records**).

##### Notes

Found inside the rhodoliths.

##### Diagnosis

(Oliveira 1951): Dark dorsum. Head with two ellipsoid eyes, twice longer than large, located close to lobe between both antennae. Pereopod coxae 1–4 of similar length and almost twice the size of the 5–7 ones (5^th^ with an anterior lobe wider than posterior). First pairs of antennae longer than second ones (Fig. 9b). First pair of gnathopods longer than second ones. Males telson similarly longer than wide, with a median cleft to about 2/3 of its length; in females, cleft close to base and as long as broad.

#### Melitidae

Bousfield, 1973

15DE0FF9-0EDB-559A-A7F9-E378A01C8442

#### Dulichiella appendiculata

(Say, 1818)

FCD21481-266E-544A-973E-81992BABD619

https://www.marinespecies.org/aphia.php?p=taxdetails&id=421504

##### Materials

- **Type status:**Other material. **Occurrence:** catalogNumber: CZAP–122; recordedBy: G. da Silva, D. Costa; individualCount: 4; **Location:** locality: Seixas Beach; verbatimDepth: 1.5 m

##### Distribution

United States of America, Mexico, Gulf of Mexico, Caribbean Sea, Cuba, Costa Rica, Venezuela, Brazilian coast (State of Paraíba), South Africa and Mozambique (Serejo and Siqueira 2018, Horton et al. 2020b; this study).

**Distribution in Paraíba**: Seixas Beach (**New record**). This species represents a new record from the Brazilian coast.

##### Notes

Found inside the rhodoliths.

##### Diagnosis

(Lowry and Springthorpe 2007): Head with two eyes; lateral cephalic lobe enlarged, truncated, antero-ventral corner with slender chaeta. Antenna 1 peduncular article 1 shorter than article 2, with 3 prominent chaetae along posterior margin. Antenna 2 peduncular article 2 cone gland reaching at least to end of peduncular article 3; article 4 slightly longer than article 5 (Fig. 9c). Mandibular palp article 1 about as long as broad, inner margin article 1 not produced distally; article 2 slightly longer than article 3.

#### Decapoda

Latreille, 1802

AB288784-0FF9-5955-B2D3-8B8498176132

#### Cyclodorippidae

Ortmann, 1892

74667E85-B305-5586-AD04-844AC9BF94E1

#### Cyclodorippe longifrons

Campos Junior & De Melo, 1999

1B516696-6AA7-51CC-8398-14B02AA3F21C

https://www.marinespecies.org/aphia.php?p=taxdetails&id=439978

##### Materials

- **Type status:**Other material. **Occurrence:** catalogNumber: CZAP–144; recordedBy: G. da Silva, D. Costa; individualCount: 1; **Location:** locality: Seixas Beach; verbatimDepth: 1.5 m

##### Distribution

Brazilian coast (Paraíba and São Paulo States) (Melo et al. 2003, WoRMS 2020b; this study).

**Distribution in Paraíba**: Seixas Beach (**New record**).

##### Notes

Found on the rhodoliths surface.

##### Diagnosis

(Campos-Junior and Melo 1999): Peduncular eyes with very reduced mobility, well-developed cornea. Subcircular carapace, adorned with fine granules. Orbital margin longer than half the maximum width of the carapace and trimmed with fine bristles. Rounded front edge with bristles, with the entire front region heavily excavated. Advanced front in relation to the orbital-external angles of the carapace. Narrow and excavated orbit, undeveloped antennular sumps (Fig. 9d).

#### Mithracidae

MacLeay, 1838

3A3F2A49-4526-5B26-8807-ADF877BA5977

#### Mithraculus forceps

Milne-Edwards, 1875

0D9CB135-0B41-56C0-BA5D-05975A42FFB8

https://www.marinespecies.org/aphia.php?p=taxdetails&id=421988

##### Materials

- **Type status:**Other material. **Occurrence:** catalogNumber: (CZAP–241), (CZAP–162); recordedBy: G. da Silva, D. Costa; individualCount: (1), (1); **Location:** locality: Miramar and Seixas Beaches; verbatimDepth: (4.0 m), (1.5 m)

##### Distribution

Gulf of Mexico to Brazilian coast (Fernando de Noronha Archipelago and Rocas Atol, Maranhão, Piauí, Ceará, Rio Grande do Norte, Paraíba, Pernambuco, Alagoas, Sergipe, Bahia, Espírito Santo, Rio de Janeiro, São Paulo, Paraná and Santa Catarina States) (Alves et al. 2012, WoRMS 2020c).

**Distribution in Paraíba**: Miramar and Seixas Beaches (**New records**).

##### Notes

Found on the rhodoliths surface.

##### Diagnosis

(Wagner 1990): The carapace is broader than long, rather flat. Branchial sulci on its surface are not or very weakly broken by transverse grooves. Rostrum is little advanced, incised by a narrow notch. The basal antennal segment has two spines, of which the second, situated on the antero-external angle, is five times as large as the first. In small individuals, two acute tips can be observed distally on the second spine. The antennae are 0.2 times as long as the carapace. The orbit is armed with one spine below (not counting the basal antennal spines), one at the outer angle and three above (Fig. 9e).

#### Paguridae

Latreille, 1802

C5CBE49E-8182-5EAD-ABBD-15636AD3AC9D

#### Pagurus criniticornis

(Dana, 1852)

6E6E73AE-EA4E-5118-95DB-450BDB8BB99C

https://www.marinespecies.org/aphia.php?p=taxdetails&id=366674

##### Materials

- **Type status:**Other material. **Occurrence:** catalogNumber: CZAP–208; recordedBy: G. da Silva, D. Costa; individualCount: 3; **Location:** locality: Maceió Beach; verbatimDepth: 4.0 m

##### Distribution

Gulf of Mexico, Antilles, northern South America, Brazil (Saint Peter and Saint Paul Archipelago, Paraíba, Pernambuco, Alagoas, Sergipe, Bahia, Espírito Santo, Rio de Janeiro, São Paulo, Paraná, Santa Catarina and Rio Grande do Sul States), Uruguay and Argentina (Nucci and DeMelo 2007, Lemaitre and McLaughlin 2020; this study).

**Distribution in Paraíba**: Maceió Beach (**New record**).

##### Notes

Found on the rhodoliths surface.

##### Diagnosis

(Nucci and DeMelo 2007): Shield slightly longer than broad. Rostrum obtuse, slightly over-reaching lateral projections. Ocular peduncles slender and shorter than shield width, with corneae slightly dilated. Ocular acicles with anterior margins rounded, with one strong submarginal spine; occasionally accessory marginal spinule on mesial margin. Antennular peduncles over-reaching corneae; antennal peduncles usually not reaching distal margins of corneae; flagella long, usually over-reaching right cheliped (Fig. 10a).

#### Xanthidae

MacLeay, 1838

9136DE37-4F95-58B2-A1CE-6FC1A51229A9

#### Garthiope spinipes

(Milne-Edwards, 1880)

45324B71-67A0-5883-A54C-7C547405B58A

https://www.marinespecies.org/aphia.php?p=taxdetails&id=422121

##### Materials

- **Type status:**Other material. **Occurrence:** catalogNumber: CZAP–110; recordedBy: G. da Silva, D. Costa; individualCount: 2; **Location:** locality: Seixas Beach; verbatimDepth: 1.5 m

##### Distribution

Florida, Gulf of Mexico, Antilles, Venezuela and Brazilian coast (Amapá, Pará, Maranhão, Piauí, Ceará, Rio Grande do Norte, Paraíba, Pernambuco, Alagoas, Sergipe, Bahia, Espírito Santo and São Paulo States) (Alves et al. 2012, Melo and Veloso 2005, WoRMS 2020d).

**Distribution in Paraíba**: Mataraca, Baía da Traição, Rio Tinto, Lucena, Cabedelo, João Pessoa and Pitimbu Municipalities (Melo and Veloso 2005) and Seixas Beach (**New record**).

##### Notes

Found on the rhodoliths surface.

##### Diagnosis

(described here): Carapace about a third wider than it is long, convex. Dorsal surface covered with green granules, stronger in front and on the edges, smaller behind; several well-marked granular lines, arranged horizontally at the front of certain areas of the dorsal surface and emphasised by a row of long silks; regions poorly indicated, but nevertheless, delimited and expanded in the previous half (Fig. 10b).

#### Isopoda

Latreille, 1817

7E0C02A7-7B6D-5CDE-9F2C-6E8F0CC18925

#### Cirolanidae

Dana, 1852

BCF605B1-75B3-581B-B18D-C1DDDE3B7329

#### Cirolana parva

Hansen, 1890

0B5000DB-7CA7-5480-96A6-87C802E423AC

https://www.marinespecies.org/aphia.php?p=taxdetails&id=220697

##### Materials

- **Type status:**Other material. **Occurrence:** catalogNumber: CZAP–210, CZAP–238; recordedBy: G. da Silva, D. Costa; individualCount: 1, 2; **Location:** locality: Miramar Beach; verbatimDepth: 1.5 m, 4.0 m

##### Distribution

Tropical Atlantic Ocean: Gulf of Mexico, West Indies, Brazilian coast (Amapá, Pará, Maranhão, Piauí, Ceará, Rio Grande do Norte, Paraíba, Pernambuco, Alagoas, Sergipe, Bahia and Espírito Santo States), Northwest Africa and Red Sea (Boyko et al. 2020, Paiva and Souza-Filho 2014).

**Distribution in Paraíba**: Miramar Beach (**New record**).

##### Notes

Found inside the rhodoliths.

##### Diagnosis

(Sidabalok and Bruce 2017): Body ventrally folded rostral process that just overlaps the anterior point of the frontal lamina, the frontal lamina always pentagonal, dorsal surfaces are smooth with brown or black chromatophores, lateral margins of pleonites 3 and 4 are posteriorly produced, the pleotelson is mostly linguiform, the uropodal rami have bifid apices and the lateral margin of the uropodal exopod has a continuous row of slender plumose setae interspersed with short, acute robust setae (Fig. 10c).

#### Stomatopoda

Latreille, 1817

9188D36E-D199-5369-ABD7-E4C949BA05C5

#### Gonodactylidae

Giesbrecht, 1910

CAF67646-9AE4-56A4-99C5-C923645D808F

#### Neogonodactylus torus

(Manning, 1969)

322CD74D-8334-59BB-A0E2-3D21CBAFFBED

https://www.marinespecies.org/aphia.php?p=taxdetails&id=408954

##### Materials

- **Type status:**Other material. **Occurrence:** catalogNumber: (CZAP–224), (CZAP–116), (CZAP–303); recordedBy: G. da Silva, D. Costa; individualCount: (2), (5), (1); **Location:** locality: Miramar, Seixas and Maceió Beaches; verbatimDepth: (4.0 m), (1.5 m), (4.0 m)

##### Distribution

Southeast from United States of America to Brazilian coast (Maranhão, Piauí, Ceará, Rio Grande do Norte, Paraíba, Pernambuco, Alagoas, Sergipe and Bahia States) (Silva 2011, WoRMS 2020e).

**Distribution in Paraíba**: Miramar, Seixas and Maceió Beaches (**New records**).

##### Notes

Found on the rhodoliths surface.

##### Diagnosis

(Albuquerque 2010): Two eyes with subglobular cornea. Carapace with slightly marked gastric sulcus. Raptorial leg with dilated dactyl at the base, slightly distally crenulated and protruded with distally serrated inner margin. Body rented dorsally. Thoracic and flat abdominal somites. Telson of *Oerstedii*-type, with intermediate marginal teeth distinct and intermediate denticles located anteriorly at the end of the intermediate tooth (Fig. 10d).

#### Echinodermata

Bruguière, 1791

57CF03D3-8BD0-54A1-B181-3A5FA1AA71BE

#### Echinoidea

Leske, 1778

3B965182-6C1B-5B7D-8DF0-C53893ADC846

#### Euechinoidea

Bronn, 1860

34A275EA-B725-572B-A5AA-165E9C3793F4

#### Camarodonta

Jackson, 1912

0ABC9341-51F1-52D7-9114-845B981C3C16

#### Echinometridae

Gray, 1855

71D9F316-4EC5-503B-B929-58352889FAA0

#### Echinometra lucunter

(Linnaeus, 1758)

69A6817C-B91B-51FD-B242-77EF5FC6A074

https://www.marinespecies.org/aphia.php?p=taxdetails&id=213380

##### Materials

- **Type status:**Other material. **Occurrence:** catalogNumber: CZAP–239; recordedBy: G. da Silva, D. Costa; individualCount: 1; **Location:** locality: Miramar Beach; verbatimDepth: 4.0 m

##### Distribution

Tropical Eastern Pacific Ocean: Mexico to Colombia; Tropical Western Atlantic Ocean: Gulf of Mexico to Venezuela and northeast to southeast Brazilian coasts (Ceará, Paraíba, Pernambuco, Alagoas, Sergipe, Bahia, Espírito Santo, Rio de Janeiro, São Paulo, Paraná and Santa Catarina States) (Prata et al. 2017, Kroh and Mooi 2020).

**Distribution in Paraíba**: Cabo Branco Beach (Gondim et al. 2008), Seixas Beach (Prata et al. 2017) and Miramar Beach (**New record**).

##### Notes

Found inside the rhodoliths.

##### Diagnosis

(Miller et al. 1995, Prata et al. 2017): Elongate oval test with two rows of large tubercules along the ambulacra and interambulacra, pairs of pores arranged in arcs of six and a large peristome. Spines long and slender, thickened at the base and sharply pointed at the tips. On aboral side, primary and secondary spines dark olive green, with greenish-violet to purple tips. In general, the colour is blackish (Fig. 11a).

#### Holothuroidea

Blainville, 1834

A9C0E3E7-231D-5331-97DE-BCD958B6EC6B

#### Apodida

Brandt, 1835

4BE4DBCD-1857-5719-B282-0939F2A4DE19

#### Chiridotidae

Östergren, 1898

9EE61ABE-044C-5F58-B8D5-3F71117CDD04

#### Chiridota rotifera

(Pourtalès, 1851)

A74A28E8-8A18-5605-956F-4F003F364D87

http://www.marinespecies.org/aphia.php?p=taxdetails&id=422538

##### Materials

- **Type status:**Other material. **Occurrence:** catalogNumber: CZAP–205; recordedBy: G. da Silva, D. Costa; individualCount: 1; **Location:** locality: Miramar Beach; verbatimDepth: 4.0 m

##### Distribution

Tropical Eastern Pacific Ocean: Mexico, Panamá coast; Tropical Western Atlantic Ocean: Gulf of Mexico to Venezuela and Brazilian coast (Ceará, Paraíba to Alagoas, Bahia and Rio de Janeiro States) (Prata et al. 2017, WoRMS 2020f).

**Distribution in Paraíba**: Cabo Branco Beach (Gondim et al. 2008), Seixas Beach (Prata et al. 2017) and Miramar Beach (**New record**).

##### Notes

Found inside the rhodoliths.

##### Diagnosis

(Prata et al. 2017): Body cylindrical, elongated. Tegument thin, with some papillae or warts formed by agglomeration of ossicles. Mouth and anus terminal. Colour light pink to translucent. Body wall with wheels with six holes. Small, straight to curved (C-shaped) rods in radial zones. Tentacles with rods similar to those of body (Fig. 11b).

#### Ophiuroidea

Gray, 1840

D8769933-5AD9-50A5-9AFA-67FF1CDCEFB1

#### Myophiuroida

Matsumoto, 1915

86CEB0D5-00B2-5AA4-82F5-2F0C8962EC02

#### Amphilepidida

O'Hara, Hugall, Thuy, Stöhr & Martynov, 2017

58CBF87C-AD67-5B65-B1B9-40E5E5DDB0F2

#### Amphiuridae

Ljungman, 1867

5F6558A4-734F-51FC-AA4B-6D70328CB787

#### Amphipholis januarii

Ljungman, 1866

2EC8D31E-D71D-5E68-A3FA-C935BA62B943

https://www.marinespecies.org/aphia.php?p=taxdetails&id=149906

##### Materials

- **Type status:**Other material. **Occurrence:** catalogNumber: (CZAP–177), (CZAP–090, CZAP–108); recordedBy: G. da Silva, D. Costa; individualCount: (1), (21, 7); **Location:** locality: Miramar and Seixas Beaches; verbatimDepth: (4.0 m), (1.5 m, 4.0 m)

##### Distribution

Southeast from United States of America (South Carolina, Florida and Texas), Gulf of Mexico, Antilles, Caribbean Sea and Brazilian coast (Pará, Ceará, Paraíba, Alagoas, Bahia, Rio de Janeiro and São Paulo States) (Prata et al. 2017, Stöhr et al. 2020a).

**Distribution in Paraíba**: Cabo Branco Beach (Gondim et al. 2008), Seixas Beach (Prata et al. 2017) and Miramar Beach (**New record**).

##### Notes

Found on the rhodoliths surface.

##### Diagnosis

(Prata et al. 2017): Disc circular to pentagonal, with re-entrances in inter-radial areas. Disc covered by small and imbricated scales. Radial shields narrow, longer than wide, usually separated by one or two scales, the internal more elongated. Ventral side of the disc covered by smaller scales, imbricated. Bursal slit long, near the first arm plate. Oral shield diamond-shaped, adoral shield triangular. Two oral papillae in each side of jaw, the distal triangular and robust, a pair of elongated and robust infradental papillae. Five elongated arms, about seven to ten times the diameter of the disc (Fig. 11c).

#### Amphipholis squamata

(Delle Chiaje, 1828)

292F456D-FB61-5430-85FD-96609A1ABD46

https://www.marinespecies.org/aphia.php?p=taxdetails&id=125064

##### Materials

- **Type status:**Other material. **Occurrence:** catalogNumber: (CZAP–242, CZAP–209), (CZAP–190); recordedBy: G. da Silva, D. Costa; individualCount: (1, 1), (1); **Location:** locality: Miramar and Seixas Beaches; verbatimDepth: (1.5 m, 4.0 m), (1.5 m)

##### Distribution

Cosmopolitan; in Brazilian coast the species was reported from Pará, Maranhão, Ceará, Paraíba, Alagoas, Bahia, Rio de Janeiro and São Paulo States (Prata et al. 2017, Stöhr et al. 2020b).

**Distribution in Paraíba**: Cabo Branco Beach (Gondim et al. 2008), Seixas Beach (Prata et al. 2017) and Miramar Beach (**New record**).

##### Notes

Found on the rhodoliths surface.

**Remarks**: Due to its wide global distribution, this species needs a systematic review.

##### Diagnosis

(Prata et al. 2017): Disc rounded, covered by medium size scales, with circular to semicircular imbricated scales. Radial shields slightly longer than wide, separated by a thin scale up to the distal region of the shields. Ventral surface of the disc covered by scales similar to dorsal scales. Bursal slits narrow, near the first plate of the arms. Diamond-shaped oral shield, adoral shields longer than wide, touching the proximal edge. Two oral papillae in each side of jaw, the more distal larger and trapezoidal, the other rounded and smaller. A pair of elongated infradental papillae. Five arms, about five times the disc diameter (Fig. 11d).

#### Microphiopholis gracillima

(Stimpson, 1854)

3B458C51-2BB9-5069-AC8D-E4078E28EB62

https://www.marinespecies.org/aphia.php?p=taxdetails&id=405791

##### Materials

- **Type status:**Other material. **Occurrence:** catalogNumber: (CZAP–175), (CZAP–104); recordedBy: G. da Silva, D. Costa; individualCount: (1), (2); **Location:** locality: Miramar and Seixas bBeaches; verbatimDepth: (4.0 m), (4.0 m)

##### Distribution

United States of America (South Carolina and Florida) Gulf of Mexico, Antilles, Caribbean Sea and Brazilian coast (Paraíba, Bahia and Rio de Janeiro States) (Prata et al. 2017, Stöhr et al. 2020c).

**Distribution in Paraíba**: Seixas Beach (Prata et al. 2017) and Miramar Beach (**New record**).

##### Notes

Found on the rhodoliths surface.

##### Diagnosis

(Prata et al. 2017): Disc rounded with indentations in the radial region. Disc covered by numerous small and imbricated scales. Radial shields narrow and elongated, joined at half of length and then separated by three scales on the proximal edge. Ventral surface of disc covered by small and imbricated scales. Bursal slit large, near the first to fourth ventral arm plate. Oral shield diamond-shaped. Adoral shield elongated and slightly wide distally. Jaws with three oral papillae, the more distal rectangular, larger than proximal papilla. Arms long, about six to eight times the diameter of the disc (Fig. 11e).

#### Ophiactidae

Matsumoto, 1915

2635384B-81D8-56F9-94CC-E00CCFBBAD8A

#### Ophiactis savignyi

(Müller & Troschel, 1842)

40A74E4F-378D-5253-9385-56E224F563D4

https://www.marinespecies.org/aphia.php?p=taxdetails&id=125122

##### Materials

- **Type status:**Other material. **Occurrence:** catalogNumber: (CZAP–230), (CZAP–189); recordedBy: G. da Silva, D. Costa; individualCount: (1), (1); **Location:** locality: Miramar and Seixas beaches; verbatimDepth: (4.0 m), (1.5 m)

##### Distribution

Indo-West and Eastern Pacific Ocean, Atlantic Ocean: South Carolina to Brazilian coast (Amapá, Pará, Maranhão, Ceará, Paraíba, Pernambuco, Alagoas, Bahia, Rio de Janeiro and São Paulo States) (Prata et al. 2017, Stöhr et al. 2020d).

**Distribution in Paraíba**: Cabo Branco Beach (Gondim et al. 2008), Seixas Beach (Prata et al. 2017) and Miramar Beach (**New record**).

##### Notes

Found on the rhodoliths surface.

##### Diagnosis

(Prata et al. 2017): Disc rounded to pentagonal, covered by medium size scales, imbricated, more numerous in the centre and in the inter-radial surface. Small rough-tipped spines scattered over the disc, more numerous at the edges. Radial shield large and triangular, occupying more than half the disc. They are united distally and separated by two scales proximally, the most internal more elongated. Ventral surface of the disc covered by small and imbricated scales. Bursal slits large. Oral shield sub-diamond-shaped. Adoral shield longer than wide, wider distally, separated proximally. Two oral papillae flattened and robust, similar in size. An apical papilla large and triangular. Six arms, about five times the diameter of the disc, tapering distally (Fig. 11f).

## Identification Keys

### Phyla of identified marine invertebrates

**Table:** 

1	Body flatworm-like; no coelom. Dorsal region cream with brown dots, more densely disposed at the median line	**Platyhelminthes** - genus *Enchiridium*
–	Coelomate body	2
2	Cylindrical metameric body, carrying parapodia and chaetae	**Annelida** (Polychaeta)
–	Without parapodia or chaetae	3
3	Body cylindrical, with anterior distinct end (‘introvert’)	** Sipuncula **
–	Non-worm-like body	4
4	Body with one or more shells	** Mollusca **
–	Non-shell body	5
5	Body with articulated exoskeleton	**Arthropoda** (Crustacea)
–	Body with calcareous endoskeleton	** Echinodermata **

Genera keys for each phylum (below) includes only taxonomic groups found in this study and they are not complete for the whole region. Additional literature should be consulted (e.g. Amaral et al. 2013, Costa et al. 2017, DeAssis et al. 2012, Lima et al. 2017, Melo and Veloso 2005, Nonato and Luna 1970, Nucci and DeMelo 2007, Prata et al. 2017, Serejo and Siqueira 2018).

### Genera key of identified annelids polychaetes

**Table:** 

1	Prostomium with a conspicuous protuberance (‘caruncle’), extending to third chaetiger	* Eurythoe *
–	Prostomium without caruncle	2
2	Five antennae; two peristomial cirri present; subacicular hooks present	* Eunice *
–	Peristomial cirri present or absent; subacicular hooks present or absent	3
3	One or three antennae; peristomial cirri and branchiae absent	* Lysidice *
–	Prostomium with five antennae; peristomial cirri and branchiae may be present	4
4	Peristomial cirri absent; subacicular hooks present	* Marphysa *
–	Peristomial cirri present; subacicular hooks absent	5
5	Two smooth peristomial cirri; branchiae with single filaments	* Palola *
–	Peristomial cirri present or absent; with or without branchiae	6
6	Proboscis with maxillary parts scissors-like with blades (jaws prionognath-type)	7
–	Jaws configuration otherwise or without jaws	8
7	Prostomium with three antennae	* Lysarete *
–	Prostomium without antennae	* Arabella *
8	Jaws eulabidognath-type (asymmetrical, posterior parts dentate to forceps-like, short carriers)	* Kinbergonuphis *
–	Proboscis with or without maxillary apparatus	9
9	Six to eight pairs of anterior modified cirri (tentacular cirri); no paragnaths on proboscis	10
–	Up to four pairs of tentacular cirri; with or without paragnaths	11
10	Two antennae; eight pairs of tentacular cirri	* Hesione *
–	Three antennae; six pairs of modified cirri	* Oxydromus *
11	Two antennae; four pairs of tentacular cirri; paragnaths on proboscis surface	12
–	With or without antennae; without paragnaths	14
12	Proboscis with paragnaths in areas II to IV and VI; prostomium deeply cleft in the anterior region; two antennae as long as prostomial width	* Ceratonereis *
–	Paragnaths with another configuration	13
13	Proboscis with paragnaths in areas I to IV and VI to VIII	* Nereis *
–	Proboscis with paragnaths in all areas	* Pseudonereis *
14	Four antennae; dorsal enlarged foliaceous-shaped cirri	* Phyllodoce *
–	Zero to three antennae	15
15	Three antennae, dorsum covered by 12 pairs of elytra	* Lepidonotus *
–	Body without elytra	16
16	Three antennae; pharynx with a tooth; a prominent proventricle	* Syllis *
–	Prostomium without antennae; sedentary polychaetes	17
17	Hooded hooks; capillaries from chaetiger 1	* Neopseudocapitella *
–	Hooks no cloaked	18
18	Prostomium T-shaped; posterior parapodia dorsally directed	* Naineris *
–	Parapodia another configuration	19
19	Merged opercular lobes in mid-ventral indentation	* Phragmatopoma *
–	Body without operculum	20
20	Prostomium with branchial crown	21
–	No branchial crown	22
21	Spots on body; 46 pairs of radioles, with stylodes	* Branchiomma *
–	12 pairs of radioles; collar divided at two regions lobe-like	* Hypsicomus *
22	Paired notched peristomial tentacular filaments (palps); branchiae present	23
–	With or without palps	25
23	3-4 palps; branchiae start from chaetiger 3	* Cirratulus *
–	Branchiae start from chaetiger 1	24
24	Multiple anterior tentacular cirri	* Cirriformia *
–	Peristomium with two segmentations; two groups of five palps	* Timarete *
25	Chaetae three first iridescent forming a cephalic cage	* Pherusa *
–	Chaetae do not form a cage	26
26	Many palps from prostomial origin	* Terebella *
–	No palps; proboscis spoon-shaped with brown streaks	* Echiurus *

### Genera key of identified sipunculids

**Table:** 

1	Anal shield with dark chalky points; margin caudal shield with irregular ridges	* Aspidosiphon *
–	With or without longitudinal muscle bands (LMBs)	2
2	Body marbled with brown flecks and bands; introvert longer than the body; numerous rows of hooks	* Phascolosoma *
–	Body with LMBs and the nephridiopores open between LMBs 4 to 8	* Sipunculus *

### Genera key of identified molluscs

**Table:** 

1	With two valves (shells); bivalves	2
–	One or multiple shells	6
2	Trigonal valves, left hinge with the usual V-shaped cardinal tooth	* Mulinia *
–	Valves and shell articulation with another morphology	3
3	Subtriangular light valves, laterally inflated; heterodont hinge with two cardinal teeth	* Phlyctiderma *
–	Dark shells	4
4	Shell with fine divercating radial ribs; interior umbones with 1-4 dysodont hinge teeth	* Brachidontes *
–	Shell with another configuration	5
5	Smooth shell, sculpture of fine concentric semi-circular rings, with two muscle scars	* Mytella *
–	Right shell operculum-shaped, smaller than left one; adductor muscle occupying 1/5 of total size	* Crassostrea *
6	Gastropod with a shell oval-shaped, slightly spiral convex sculptures with axial ventricular ribs	* Parvanachis *
–	Many shells (polyplacophoran), tegument with multiple white spots mainly on apical region	* Acanthochitona *

### Genera key of identified arthropods crustaceans

**Table:** 

1	Body laterally narrow; thoracic appendages uniramous; amphipods	2
–	Presence of carapace; pedunculated or sessile eyes	3
2	Antenna 1 longer than 2; peduncular article 1 shorter than 2 ; flagellum with 24 articles	* Elasmopus *
–	Antenna 1 peduncular article 1 shorter than article 2, with 3 chaetae along posterior margin	* Dulichiella *
3	Five pairs of pereiopods (decapods); head fused with the thorax (cephalothorax)	4
–	Carapace reduced to anterior end	7
4	Widened flattened carapace; reduced abdomen underneath the thorax; brachyurans	5
–	Carapace longer than broad; rostrum with lateral projections; thin ocular peduncle; hermit crab	* Pagurus *
5	Subcircular carapace with fine granules, orbital margin longer than half of the carapace	* Cyclodorippe *
–	Carapace pentagonal or subpentagonal	6
6	Rostrum is little advanced, incised by a narrow notch; basal antennal segment has two spines	* Mithraculus *
–	Carapace about a third wider than it is long, convex; dorsal surface covered with green granules	* Garthiope *
7	Isopod with body ventrally folded rostral process, overlapping the frontal pentagonal lamina	* Cirolana *
–	Carapace with marked gastric sulcus; basal raptorial leg with dilated dactyl; stomatopod	* Neogonodactylus *

### Genera key of identified echinoderms

**Table:** 

1	Sea urchin with elongate oval test with two rows of large tubercules; spines long and slender	* Echinometra *
–	Sea cucumber (holothuroid) or brittle stars (ophiuroids) echinoderms	2
2	Holothuroid; tegument thin, with papillae or warts formed by agglomeration of ossicles	* Chiridota *
–	Body with a central disc, presenting five or six long and flexible arms; ophiuroids	3
3	Disc with five arms	4
–	Disc with six arms, about five times the diameter of the disc	* Ophiactis *
4	Radial shields separated by one to two scales	* Amphipholis *
–	Radial shields partially joined, separated by three scales; five arms 6-8 times the disc diameter	* Microphiopholis *

## Discussion

This study is the first to systematically describe the invertebrate species associated with rhodolith beds for northeast Brazil, on the Paraíba coast, with addition of new records for this State, including new occurrences to the Western Atlantic Ocean. Indeed, 46 species were identified in Seixas Beach (mostly composed by polychaetes), 23 in Miramar and 11 in Maceió. The first species described was *Sabellariacorallinea* Dos Santos, Riul, Brasil & Christoffersen, 2011 (Read and Fauchald 2020, DosSantos et al. 2011), which is considered endemic for the Paraíba State, being found exclusively associated with these habitats (DosSantos et al. 2011). The results for the Seixas Beach were in agreement with previous studies, which have identified 49 species of polychaetes for this Beach, with 10 new occurrences for the South Atlantic Ocean and 23 from the Paraíba coast (Costa et al. 2017). Furthermore, a new eunicid species named *Leodicecalcaricola* Bergamo, Carrerette, Zanol & Nogueira, 2018 was described for João Pessoa, Conde and Pitimbu Municipalities (Bergamo et al. 2018). Regarding echinoderms, 12 species were reported for Seixas Beach (João Pessoa) (Prata et al. 2017), including the six species of the present study. Therefore, an important diversity has been found associated with the rhodolith beds from the coast of the State of Paraíba, with Polychaeta species being most representative, particularly in the Miramar and Seixas Beaches.

Despite their importance, rhodolith habitats are still poorly studied, particularly in relation to the direct anthropogenic impacts to which they are subjected (e.g. super-exploitation, oil exploration, pollution, tourism, trawl fishing (Riul et al. 2008)), as well as to more indirect ones, such as those related to the climate crisis (e.g. global warming and ocean acidification (Horta et al. 2016, Riosmena-Rodríguez 2017)). These events modify the physical-chemical parameters of the water, which may compromise the rhodolith banks and, hence, the associated biota (Horta et al. 2015), affecting survival and levels of calcification and photosynthesis, causing the bleaching phenomenon (Martin et al. 2013, Martin and Gattuso 2009). Therefore, the conservation of these algae (and habitats) is critical, because this action may guarantee the habitat conservation of a large diversity of marine fauna (Costa et al. 2019, OSPAR-Commission 2010), using, for example, tools for promoting environmental awareness and ocean literacy (Costa et al. 2021).

Overall, this study may be regarded as baseline information on the rhodolith associated communities from this tropical region and highlights the importance of knowing and understanding their diversity levels, with the ultimate aim of promoting conservation of this important biogenic habitat. Rhodoliths beds, being considered sensitive habitats to anthropogenic effects and sheltering a rich diversity, need further studies of their associated fauna. In addition, knowing the existing fauna of a still little known habitat, essentially in the studied area, we may try to contribute to the fourteenth objective of the ‘Sustainable Development Goals’ (Sustainable-Development-Goals 2020).

### Conclusions

The beaches from the coast of the State of Paraíba had a total 57 species of invertebrates from different main taxa, associated with rhodolith beds. The species of Polychaeta were the most representative in Miramar and Seixas Beaches, while molluscs were found mainly at Maceió Beach. This knowledge about the local fauna diversity may be regarded as baseline information for a variety of purposes, to know and understand local diversity levels associated with this little-known habitat in the regions, as well as to promote environmental education actions, with the objective of making local residents and beach-goers aware of the conservation of local coastal environments.

## Supplementary Material

141D3C10-9B41-55E2-9345-17FFD557C3ED 10.3897/BDJ.9.e62736.suppl1 Supplementary material 1 Authorisation for the collection of invertebrates Data type authorisation collection Brief description Official certification provided by the Brazilian Ministry of the Environment File: oo_486635.pdf https://binary.pensoft.net/file/486635 Dimítri de Araújo Costa, Marina Dolbeth, Jessica Prata, Francisco de Assis da Silva, Geuba Maria Bernardo da Silva, Paulo Ragner Silva de Freitas, Martin Lindsey Christoffersen, Silvio Felipe Barbosa de Lima, Karina Massei, Reinaldo Farias Paiva de Lucena

## Figures and Tables

**Figure 2a.**
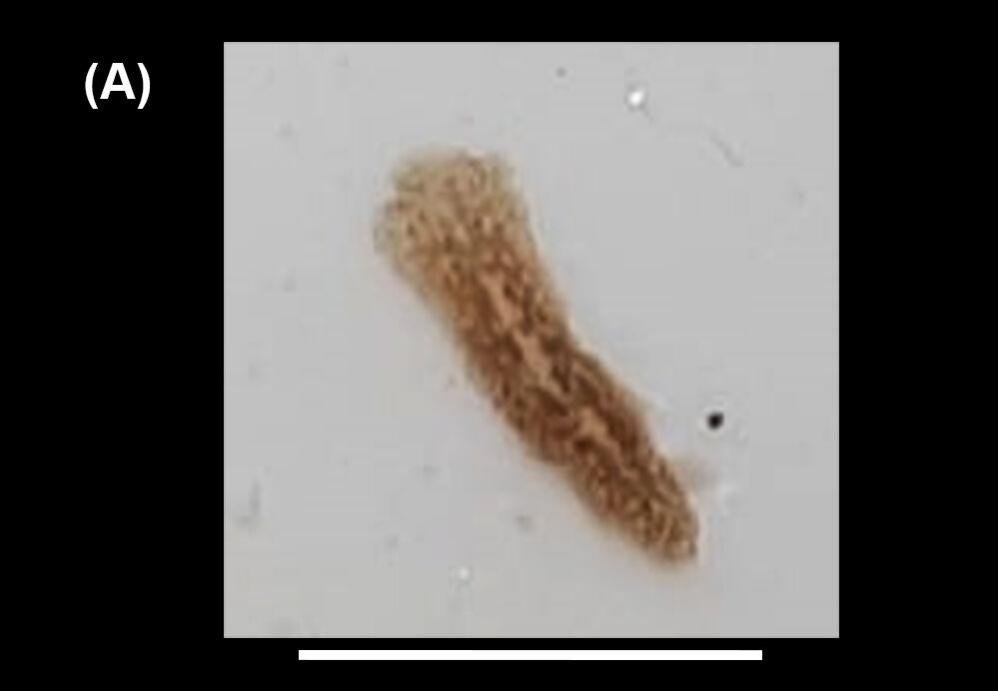
*Enchiridiumevelinae* Marcus, 1949

**Figure 2b.**
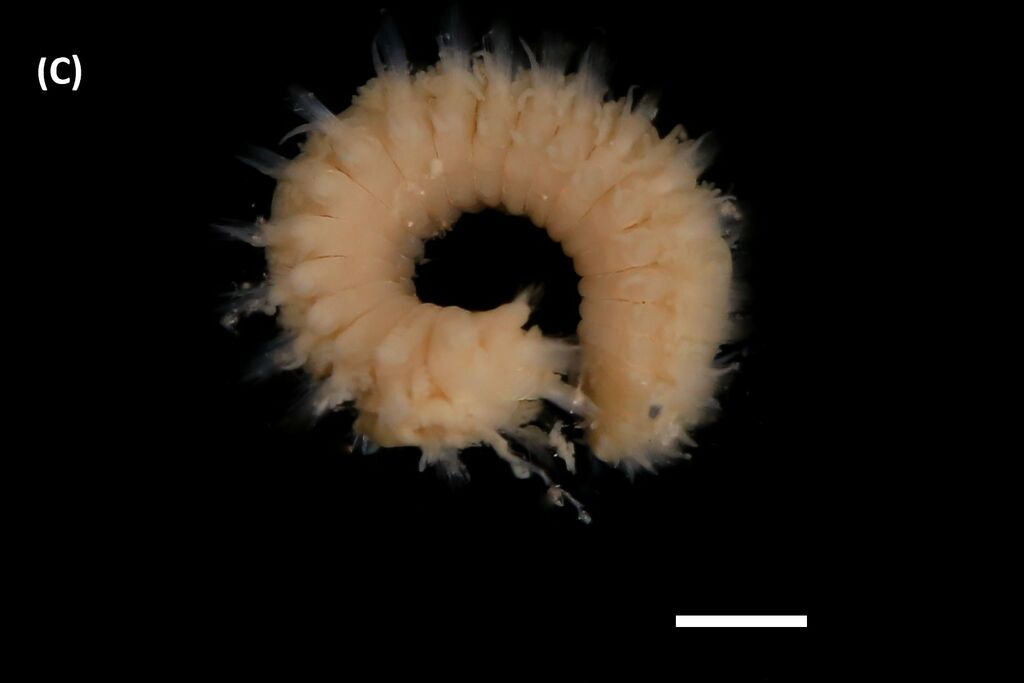
*Eurythoecomplanata* (Pallas, 1766)

**Figure 2c.**
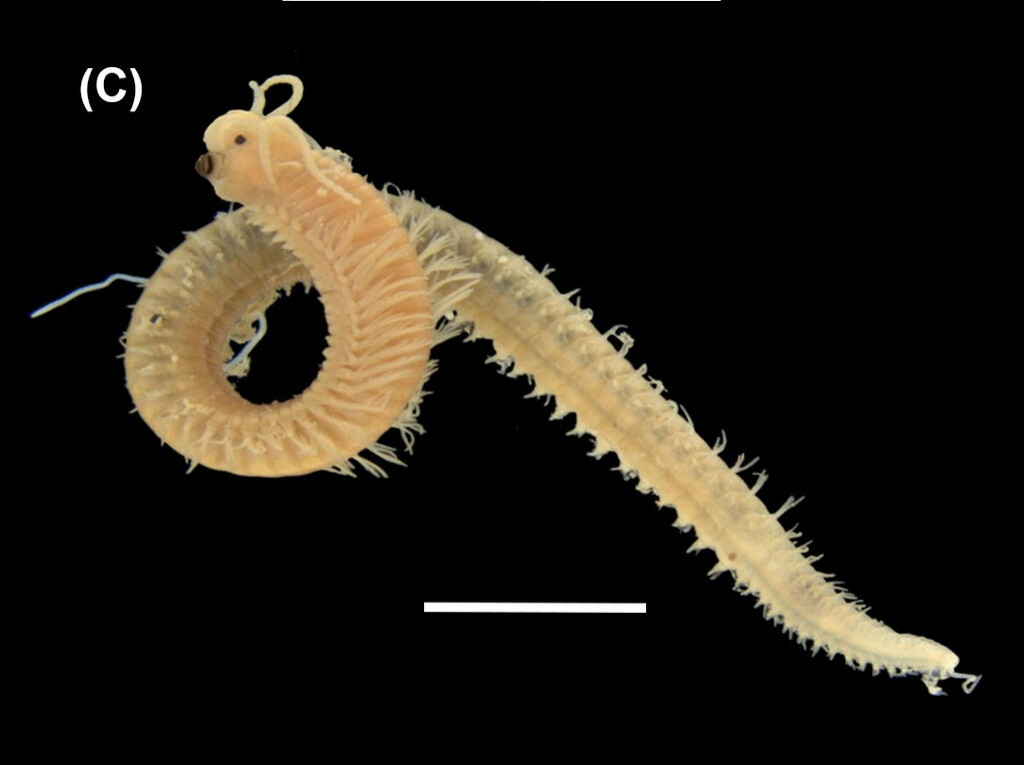
*Eunicebiannulata* Moore, 1904

**Figure 2d.**
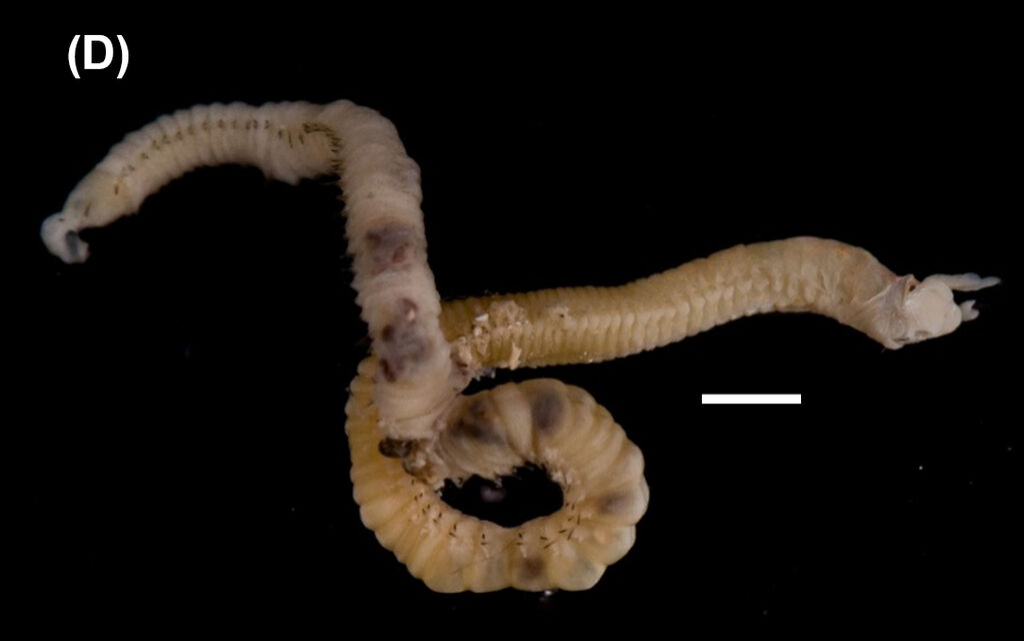
*Eunicewasinensis* Fauchald, 1992

**Figure 2e.**
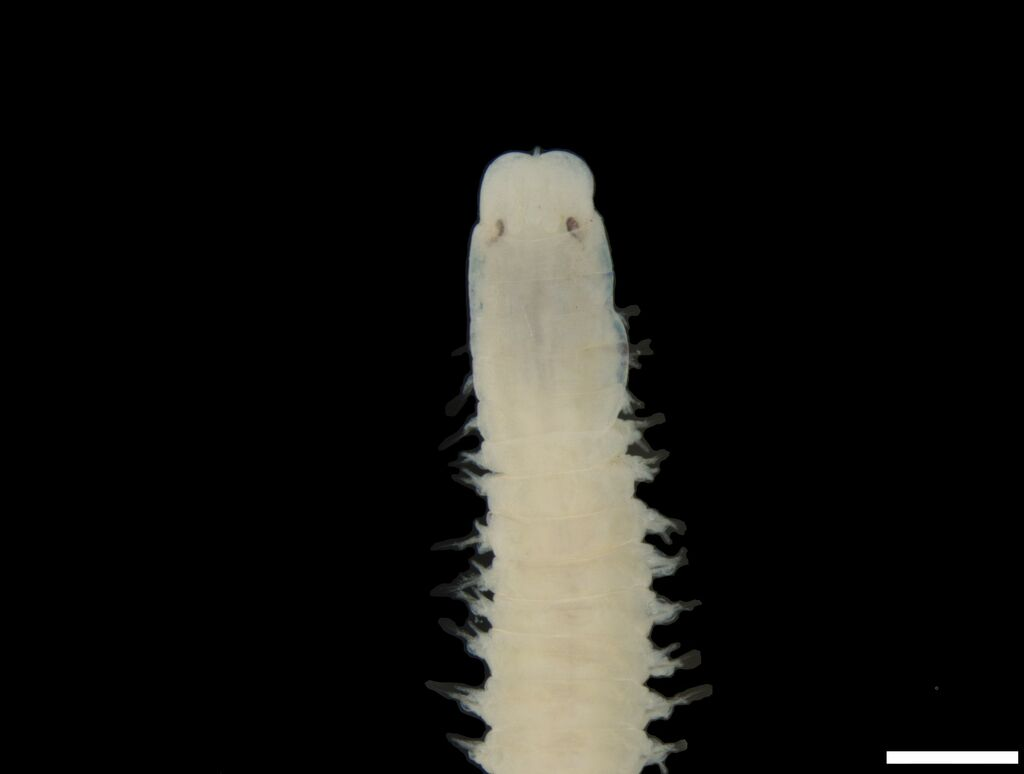
*Lysidiceninetta* Audouin & Milne Edwards, 1833

**Figure 2f.**
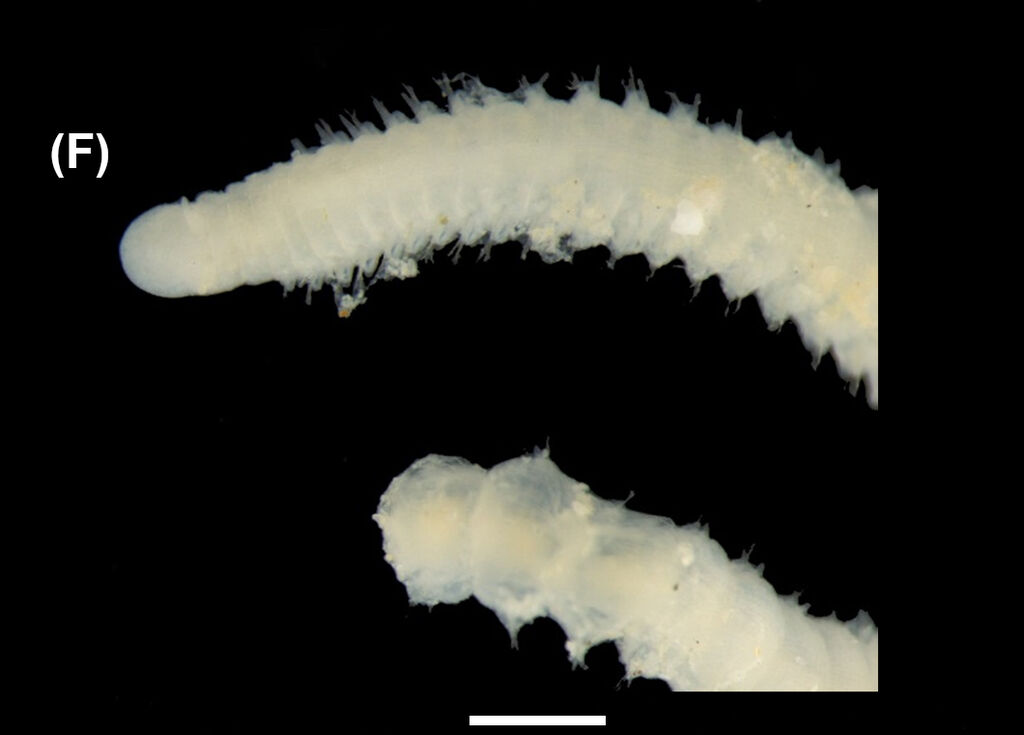
*Lysidiceunicornis* (Grube, 1840)

**Figure 3a.**
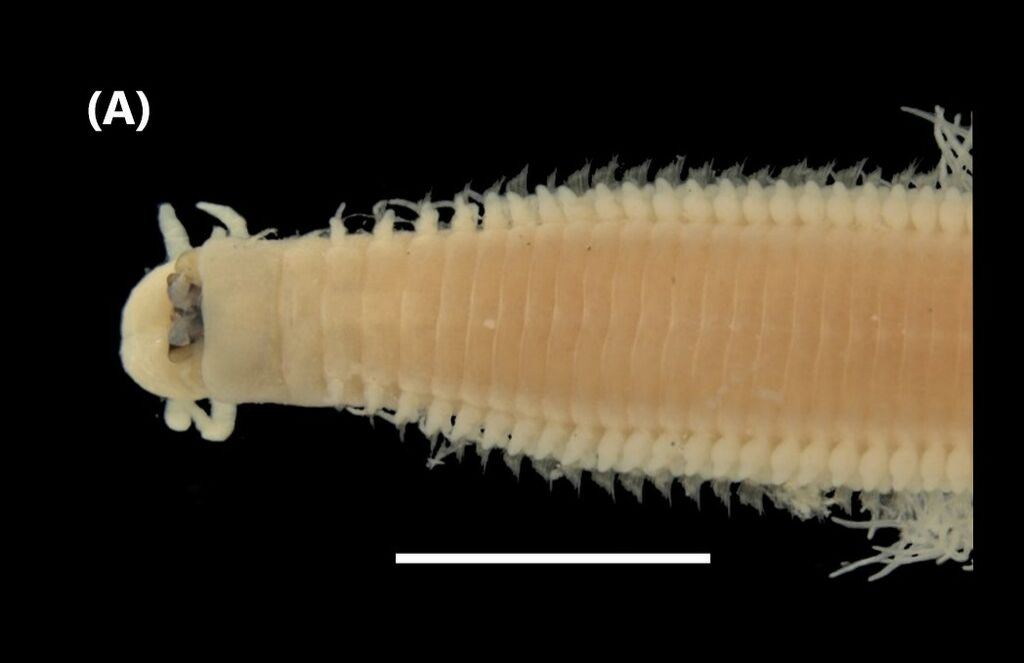
*Marphysaangelensis* Fauchald, 1970

**Figure 3b.**
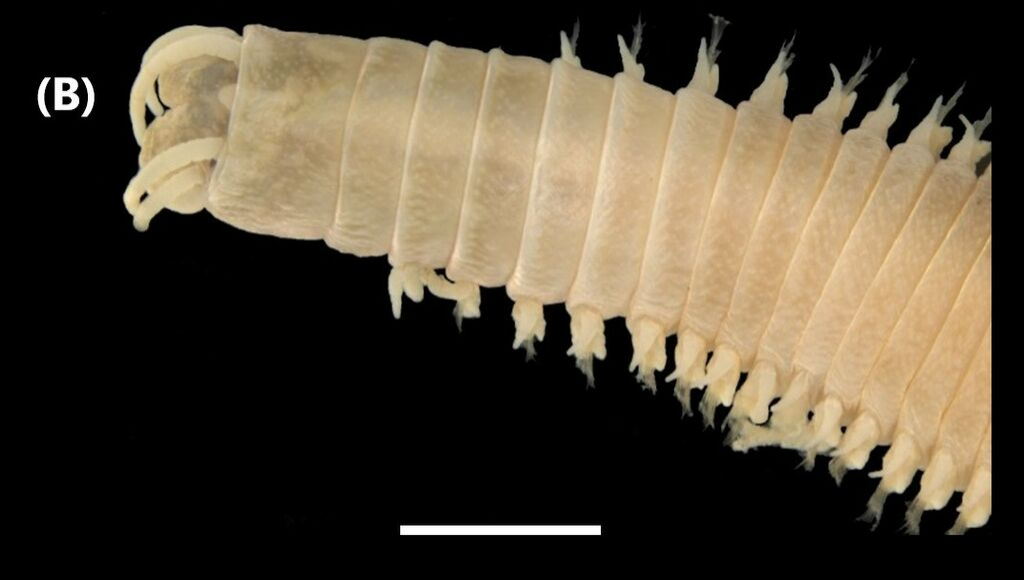
*Marphysaregalis* Verrill, 1900

**Figure 3c.**
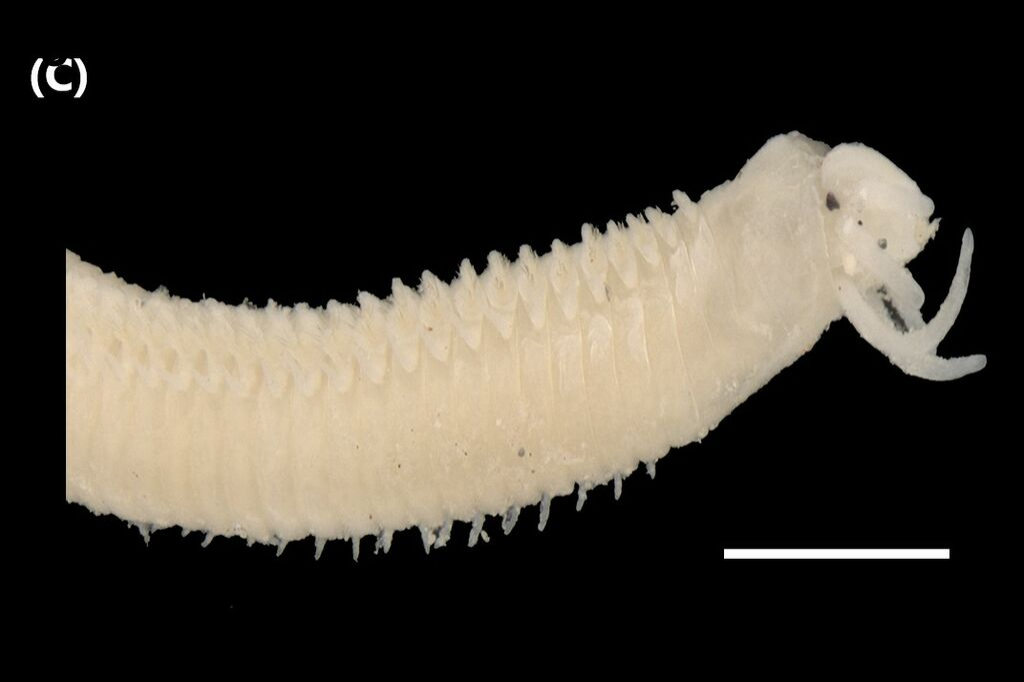
*Marphysastylobranchiata* Moore, 1909

**Figure 3d.**
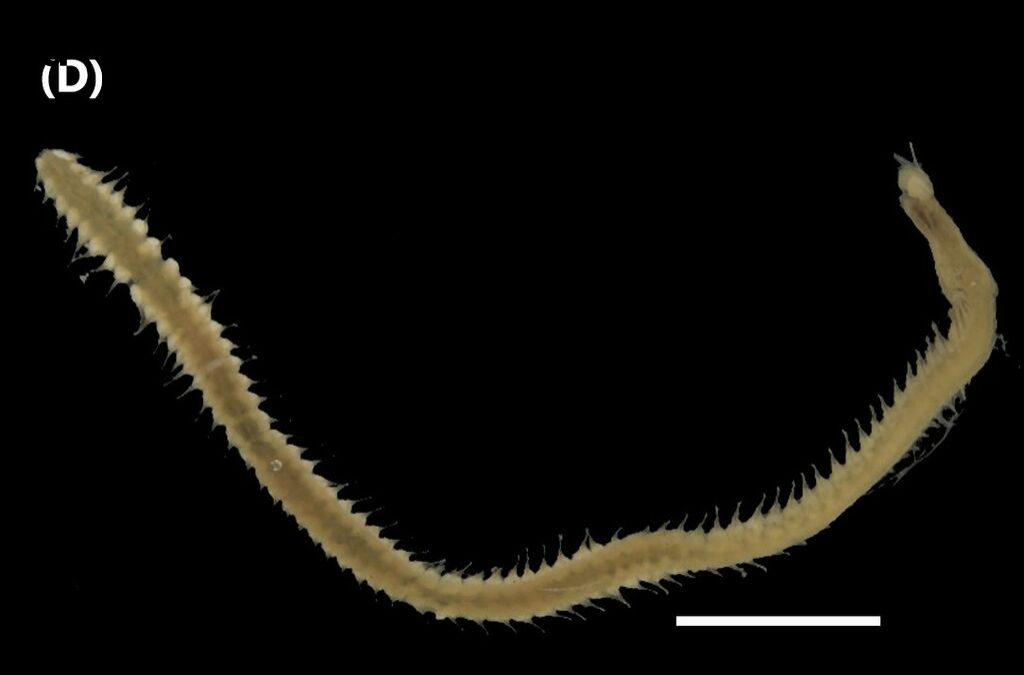
*Palolabrasiliensis* Zanol, Paiva & Attolini, 2000

**Figure 3e.**
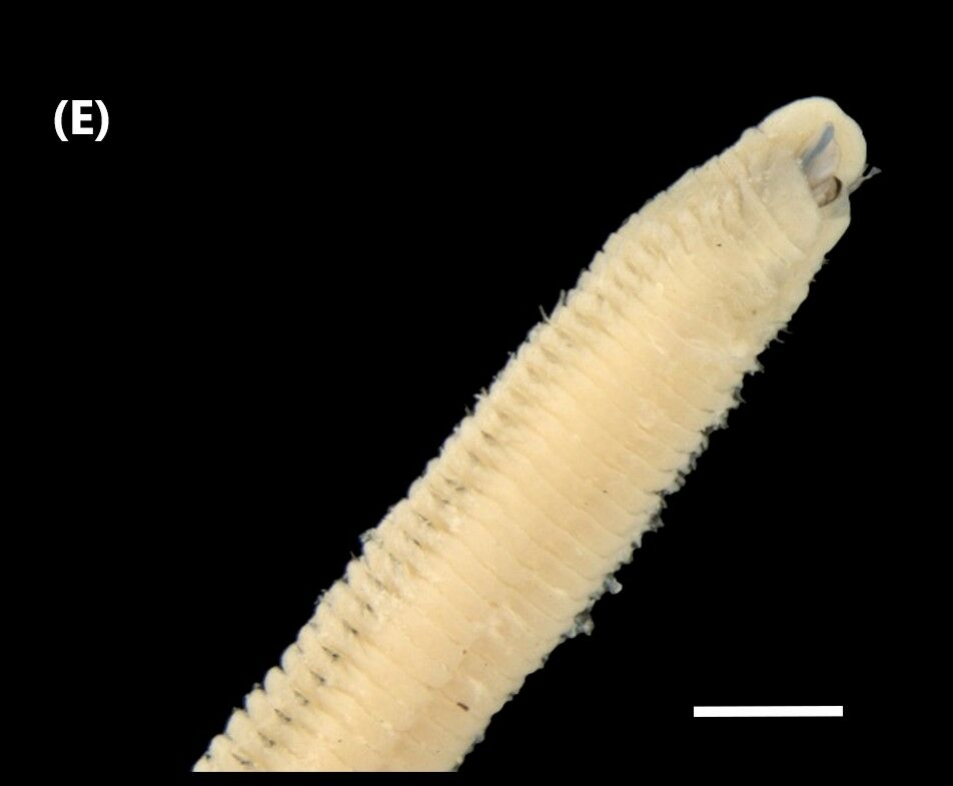
*Lysaretebrasiliensis* Kinberg, 1865

**Figure 3f.**
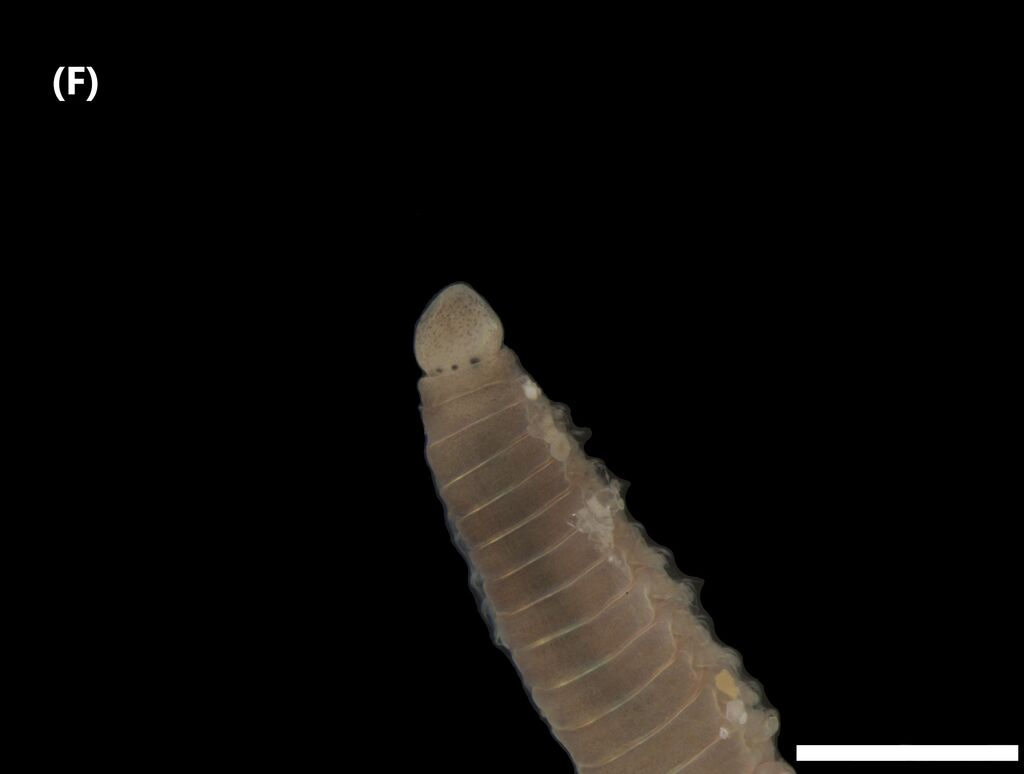
*Arabellairicolor* (Montagu, 1804)

**Figure 4a.**
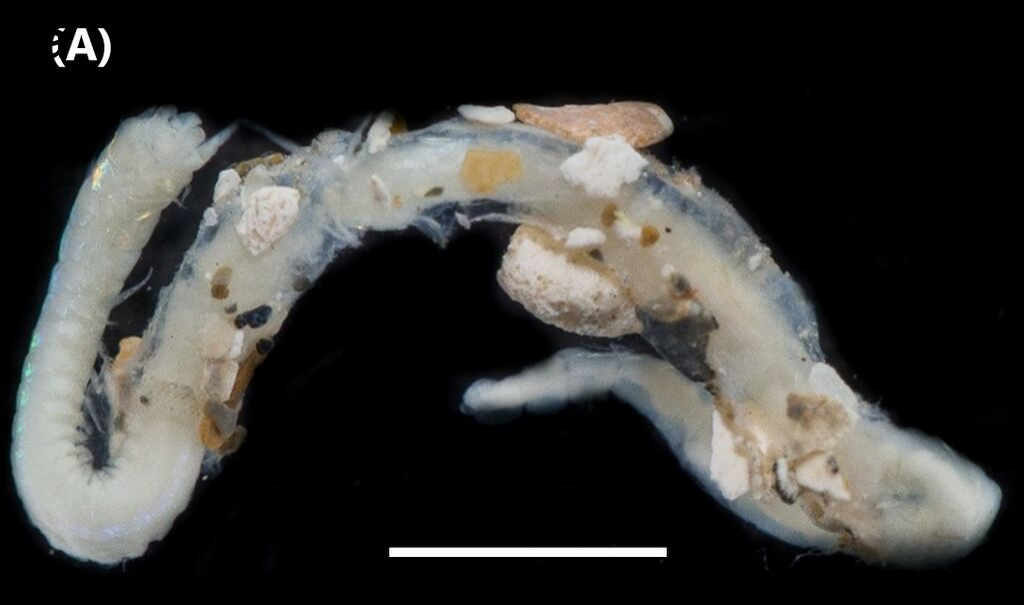
*Kinbergonuphisnonatoi* Lana, 1991

**Figure 4b.**
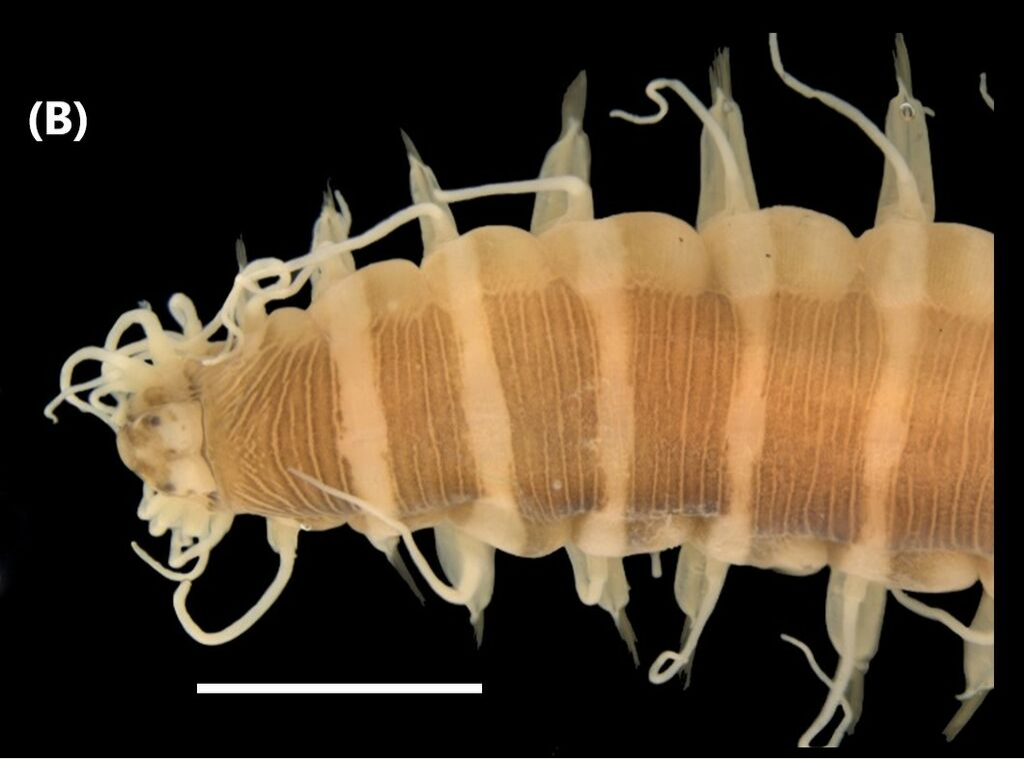
*Hesionesplendida* Lamarck, 1818

**Figure 4c.**
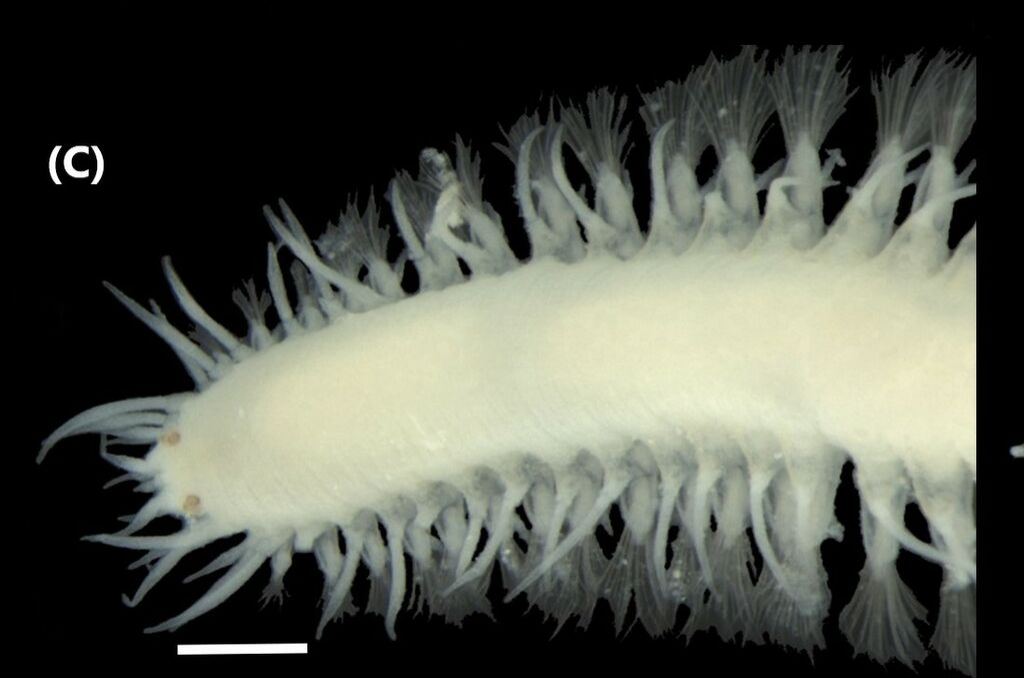
*Oxydromuspugettensis* (Johnson, 1901)

**Figure 4d.**
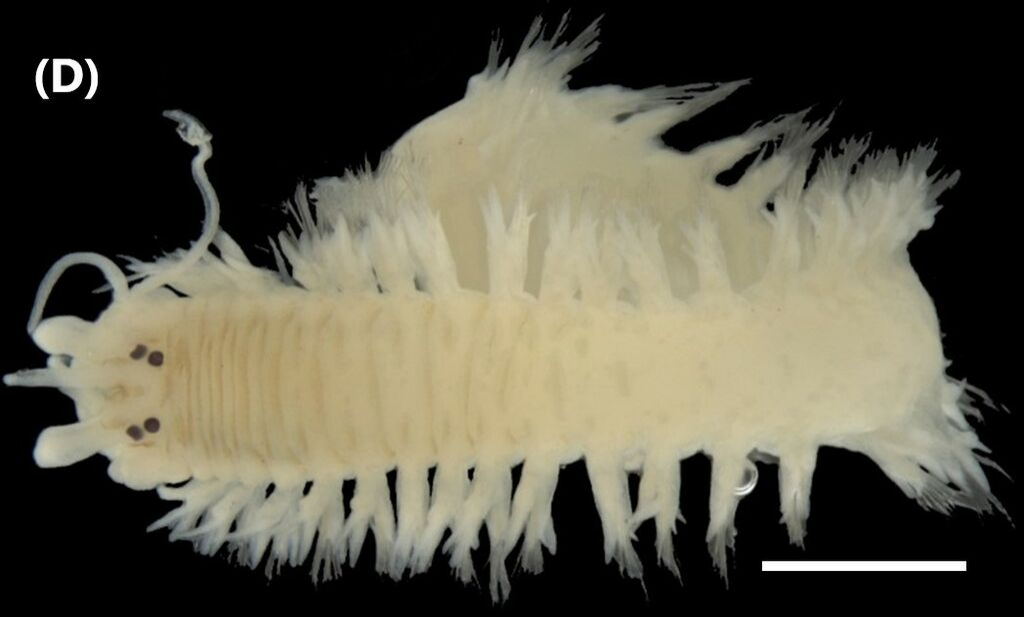
*Ceratonereissingularis* Treadwell, 1929

**Figure 4e.**
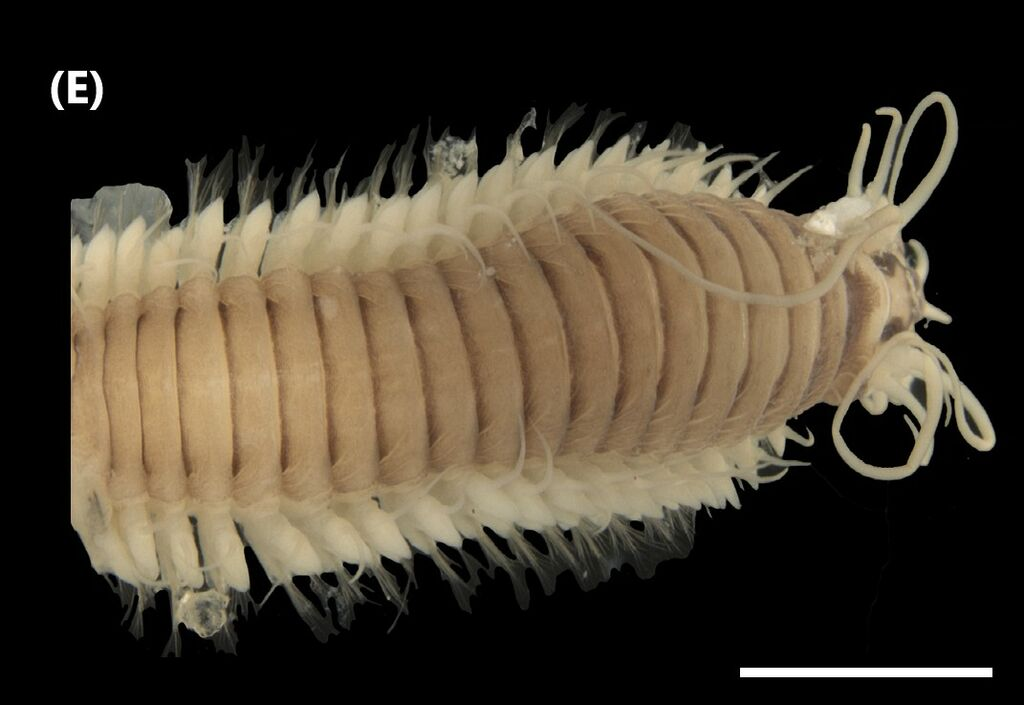
*Nereisriisei* Grube, 1857

**Figure 4f.**
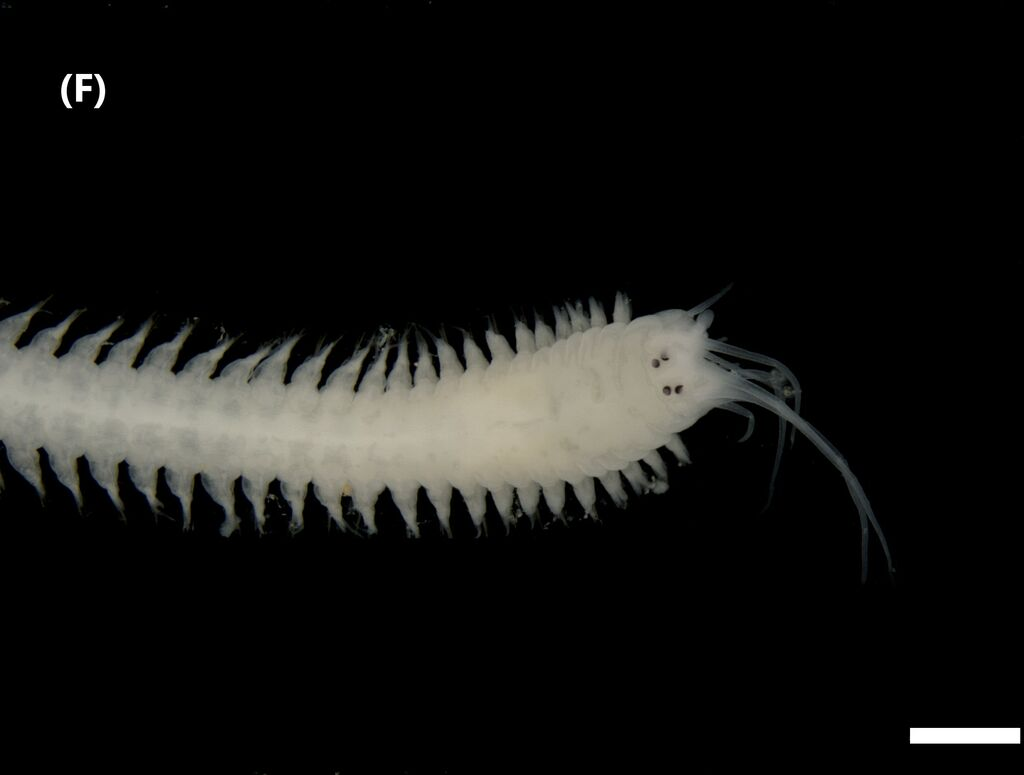
*Pseudonereisgallapagensis* Kinberg, 1865

**Figure 5a.**
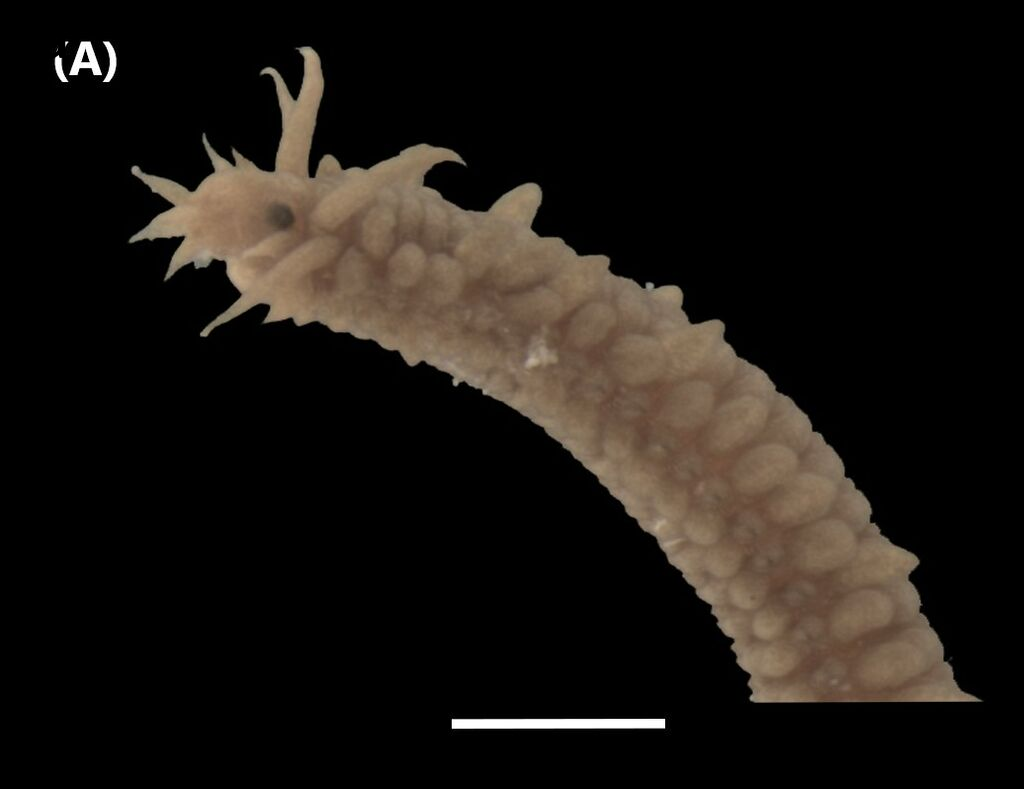
*Phyllodoceschmardaei* Day, 1963

**Figure 5b.**
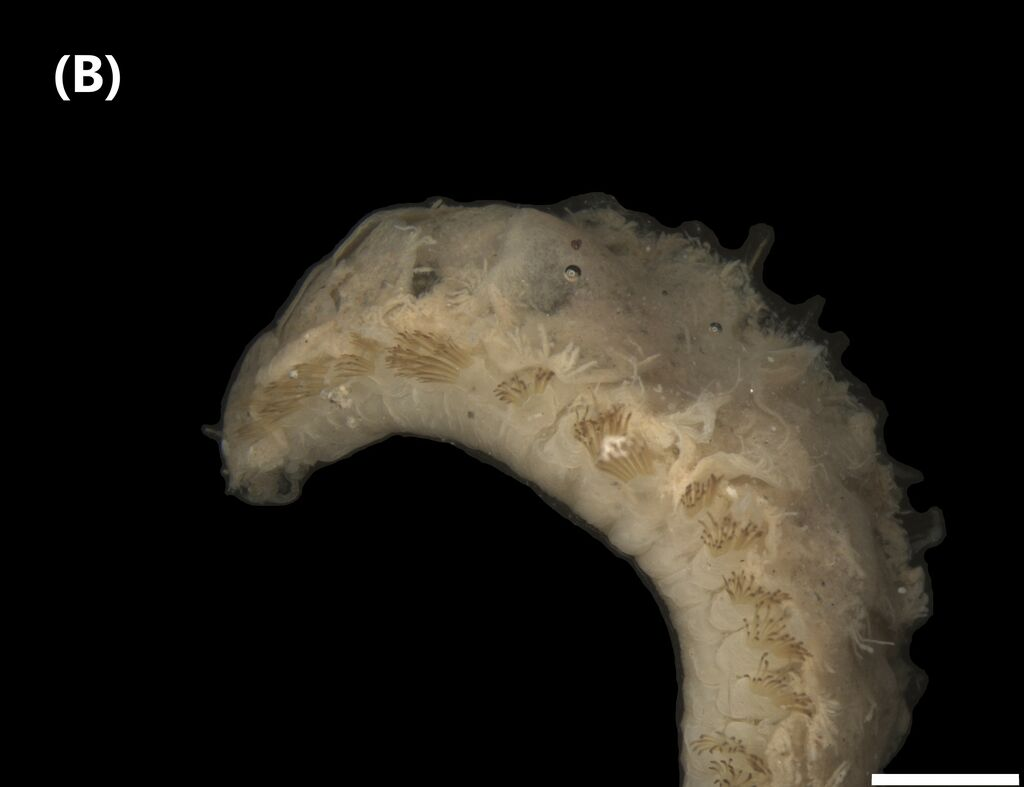
*Lepidonotussquamatus* (Linnaeus, 1758)

**Figure 5c.**
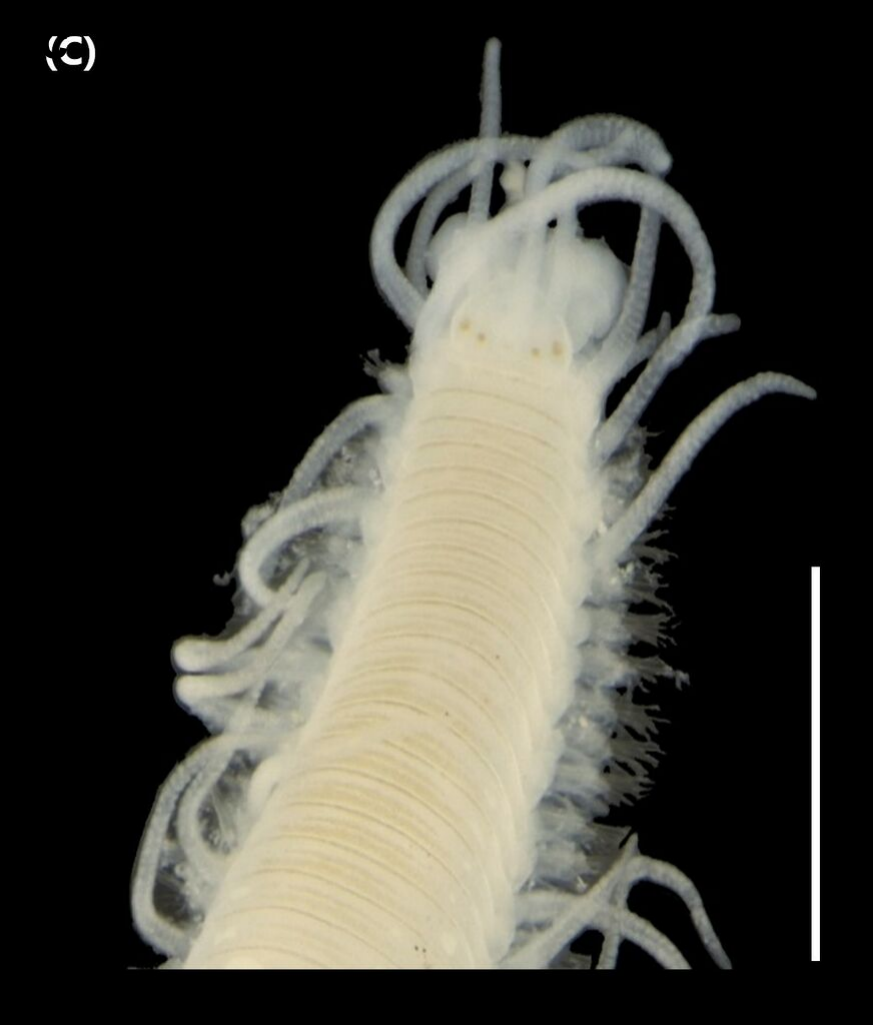
*Syllisguidae* Nogueira & Yunda-Guarin, 2008

**Figure 5d.**
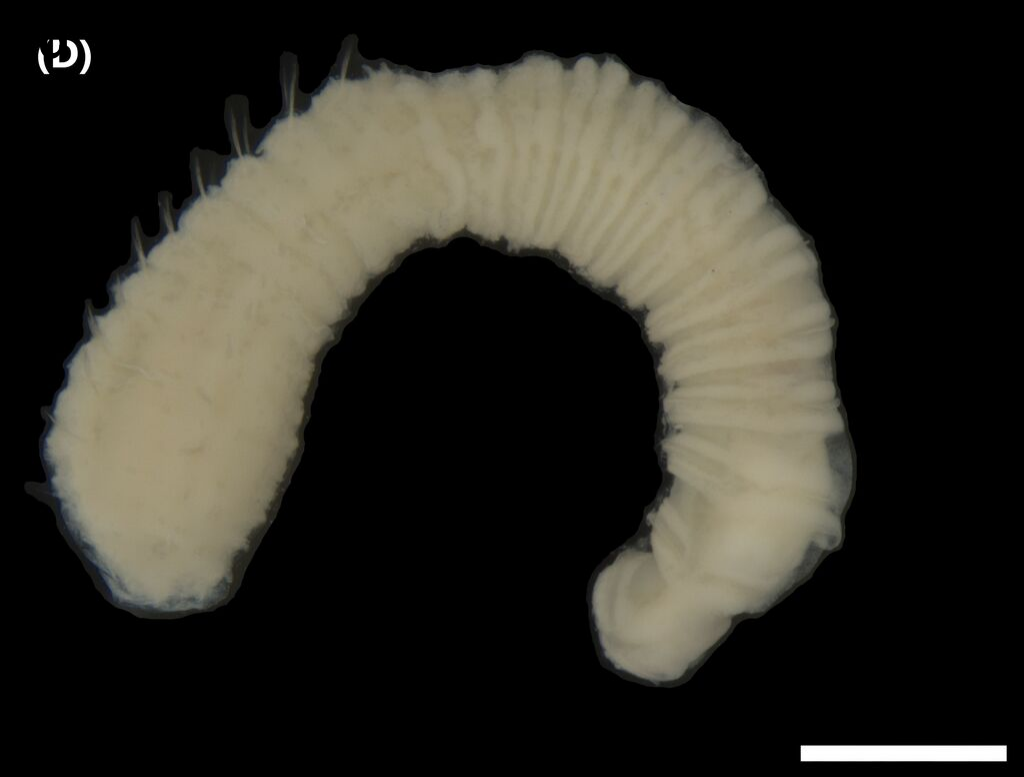
*Neopseudocapitellabrasiliensis* Rullier & Amoureux, 1979

**Figure 5e.**
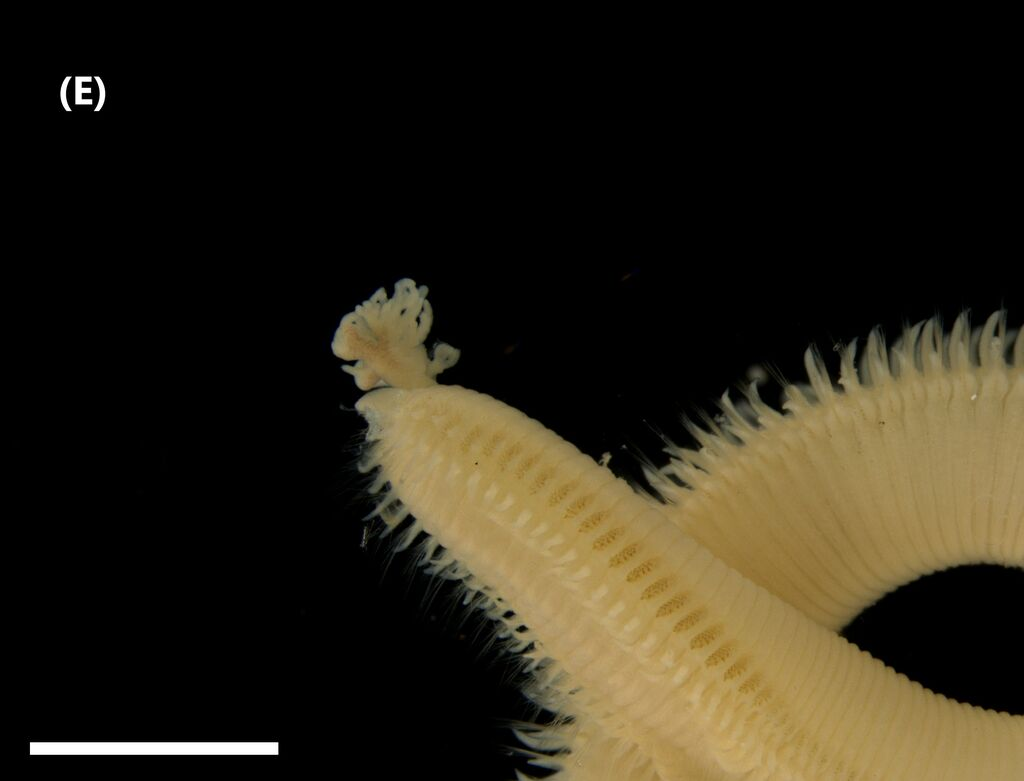
*Nainerissetosa* (Verrill, 1900)

**Figure 5f.**
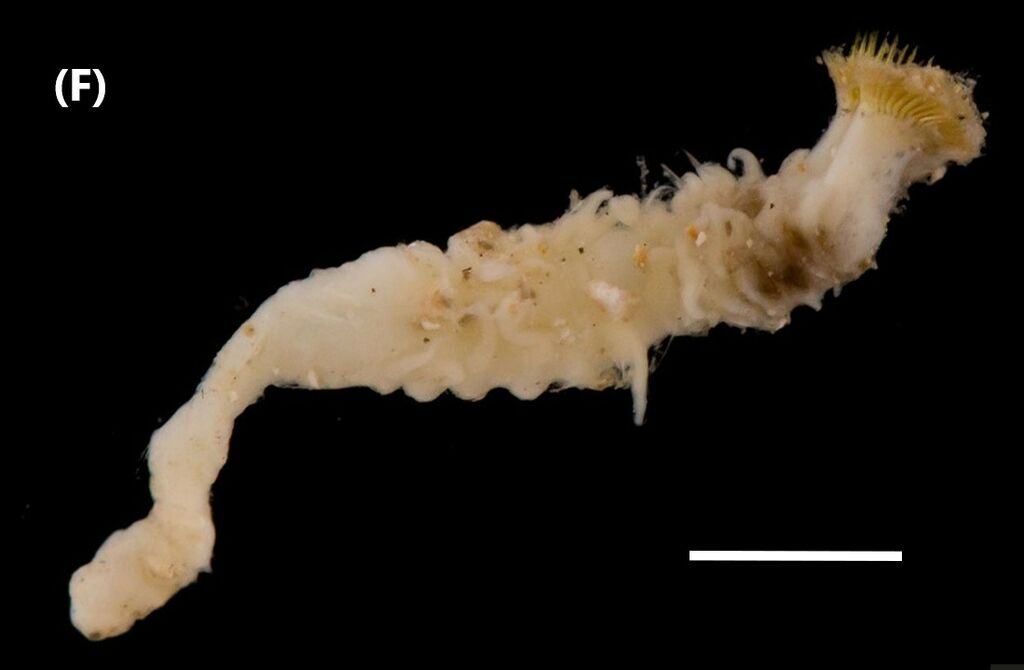
*Phragmatopomacaudata* Krøyer in Mörch, 1863

**Figure 6a.**
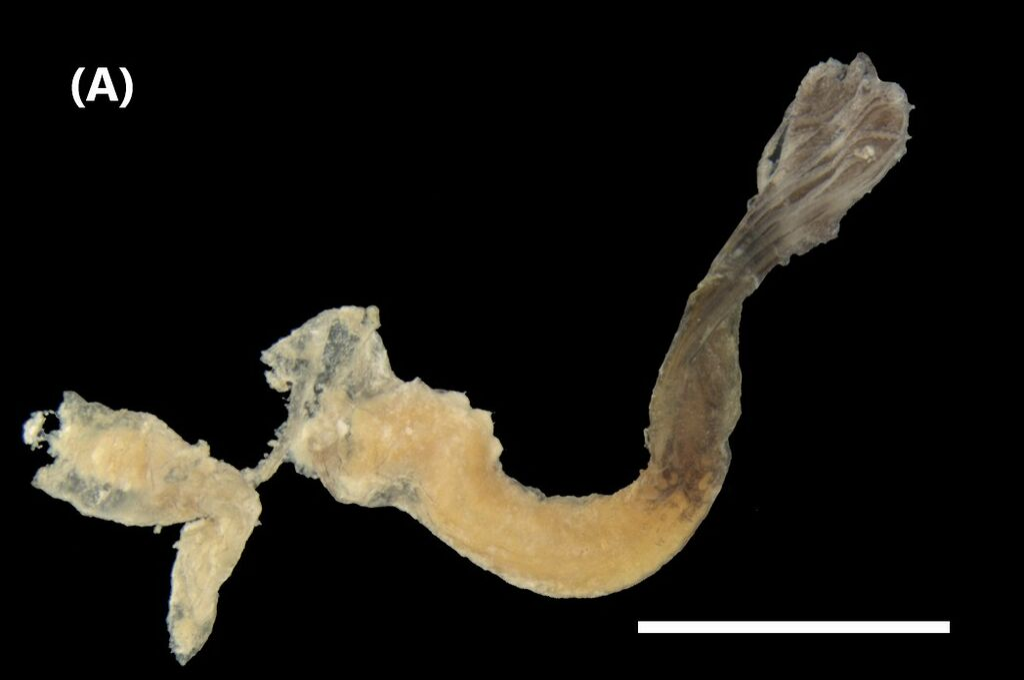
*Branchiommanigromaculatum* (Baird, 1865)

**Figure 6b.**
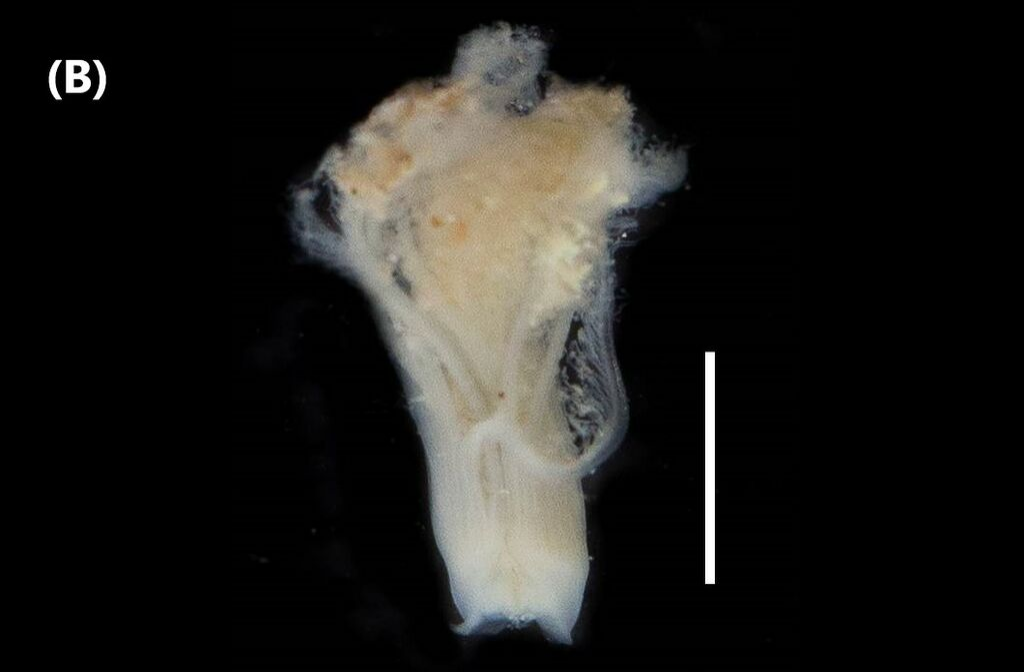
*Hypsicomuscapensis* Day, 1961

**Figure 6c.**
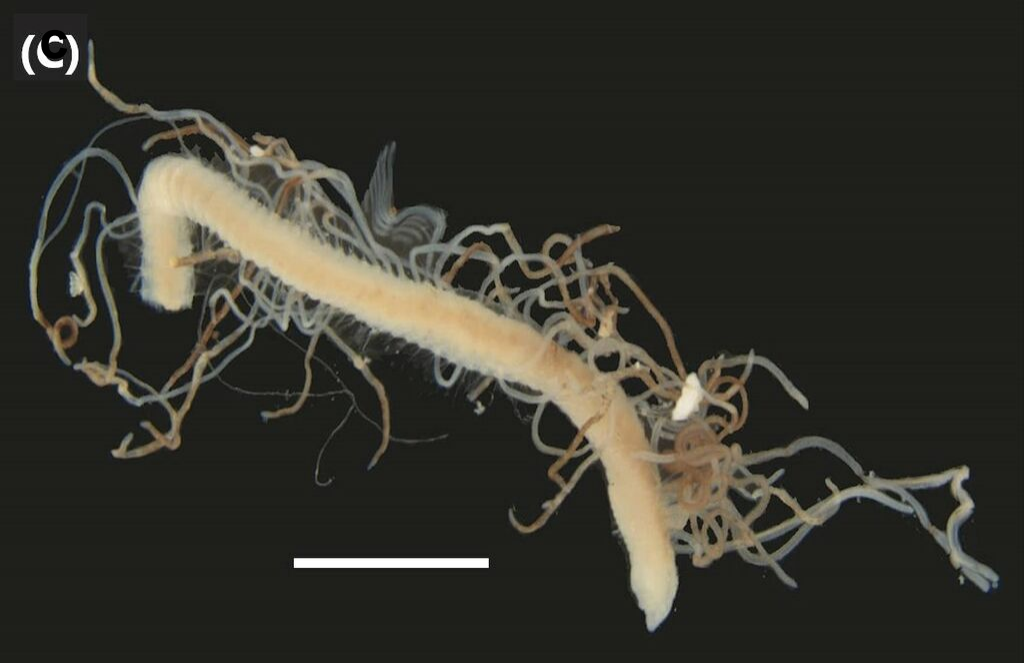
*Cirratulusafricanus* Gravier, 1906

**Figure 6d.**
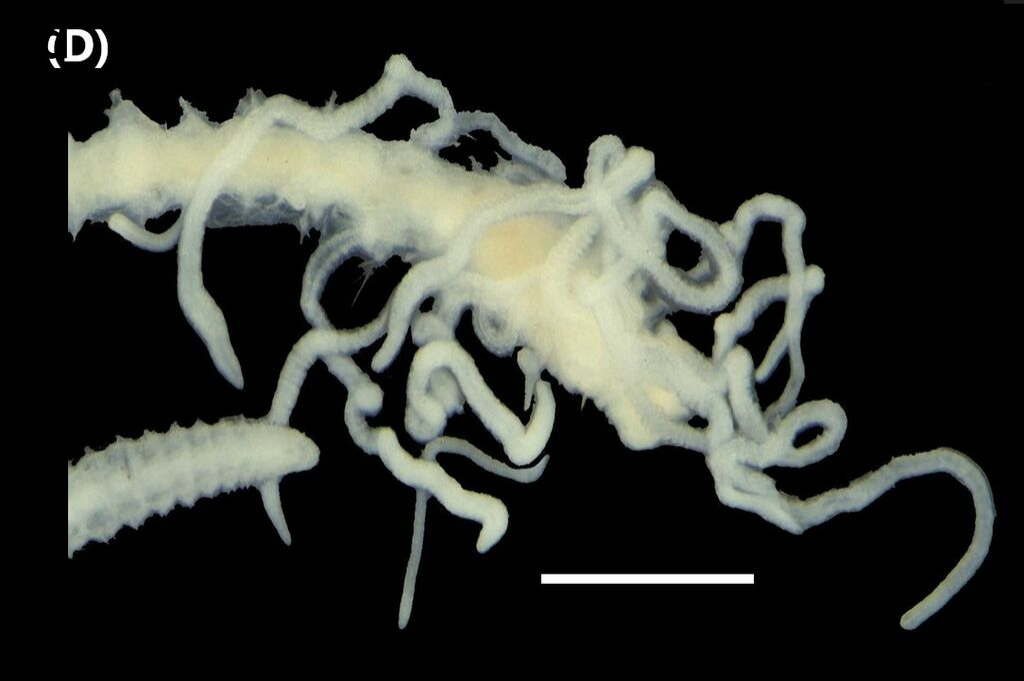
*Cirriformiacapensis* (Schmarda, 1861)

**Figure 6e.**
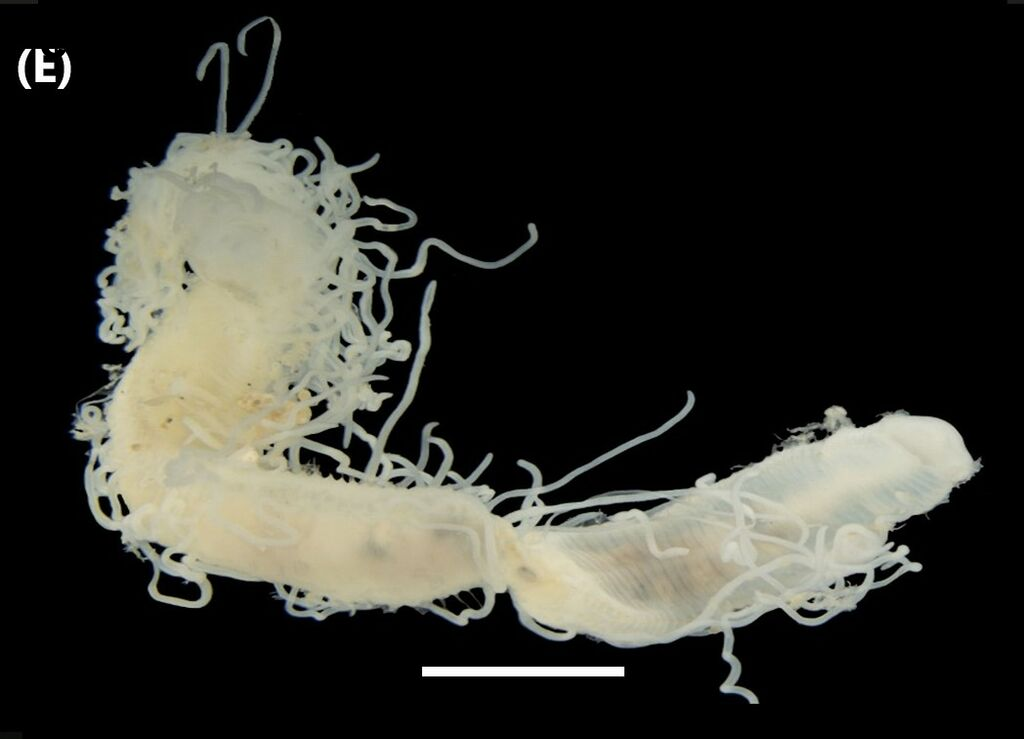
*Timaretepunctata* (Grube, 1859)

**Figure 6f.**
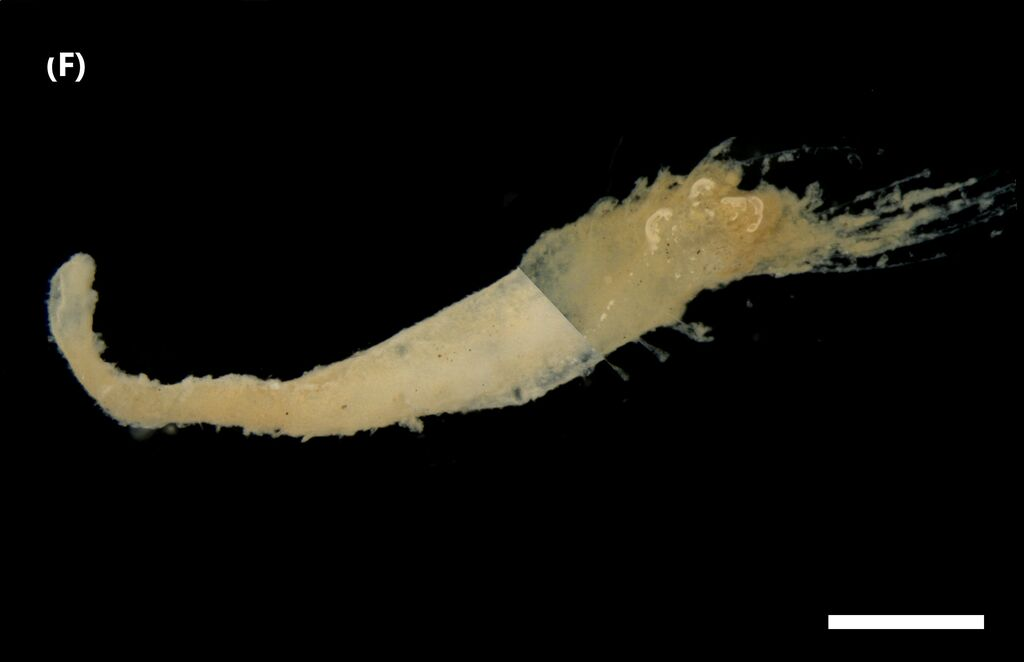
*Pherusascutigera* (Ehlers, 1887)

**Figure 7a.**
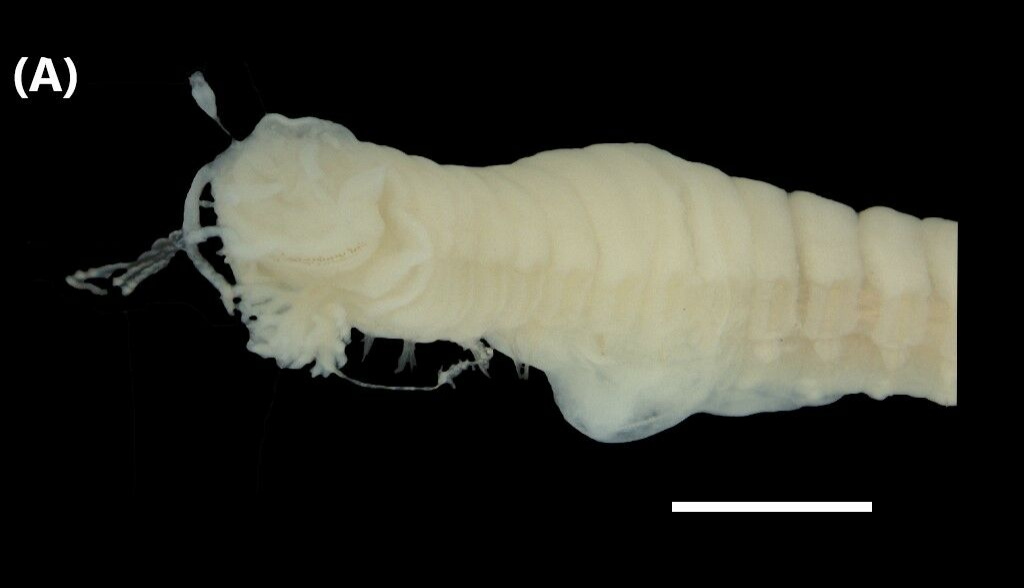
*Terebellaplagiostoma* Schmarda, 1861

**Figure 7b.**
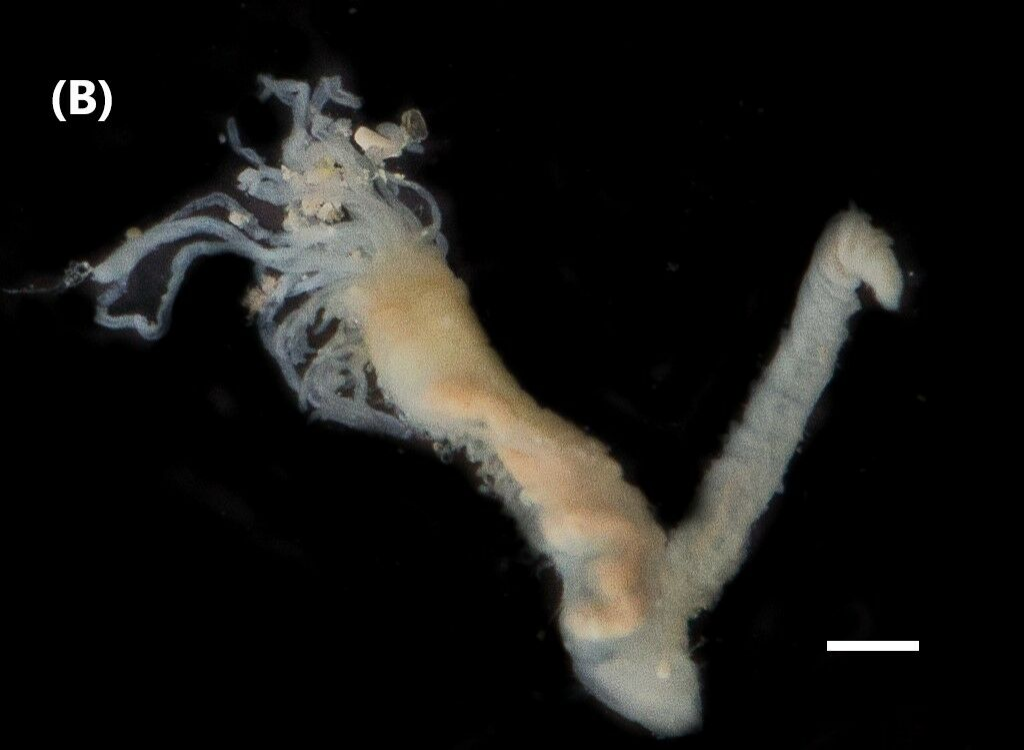
*Terebellapterochaeta* Schmarda, 1861

**Figure 7c.**
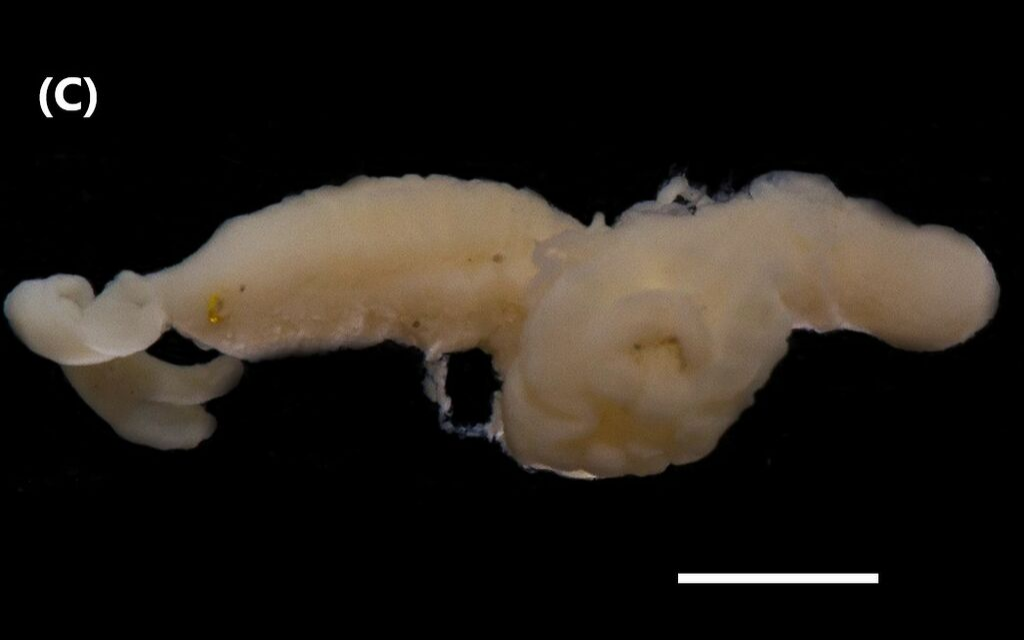
*Echiurusechiurus* (Pallas, 1766)

**Figure 7d.**
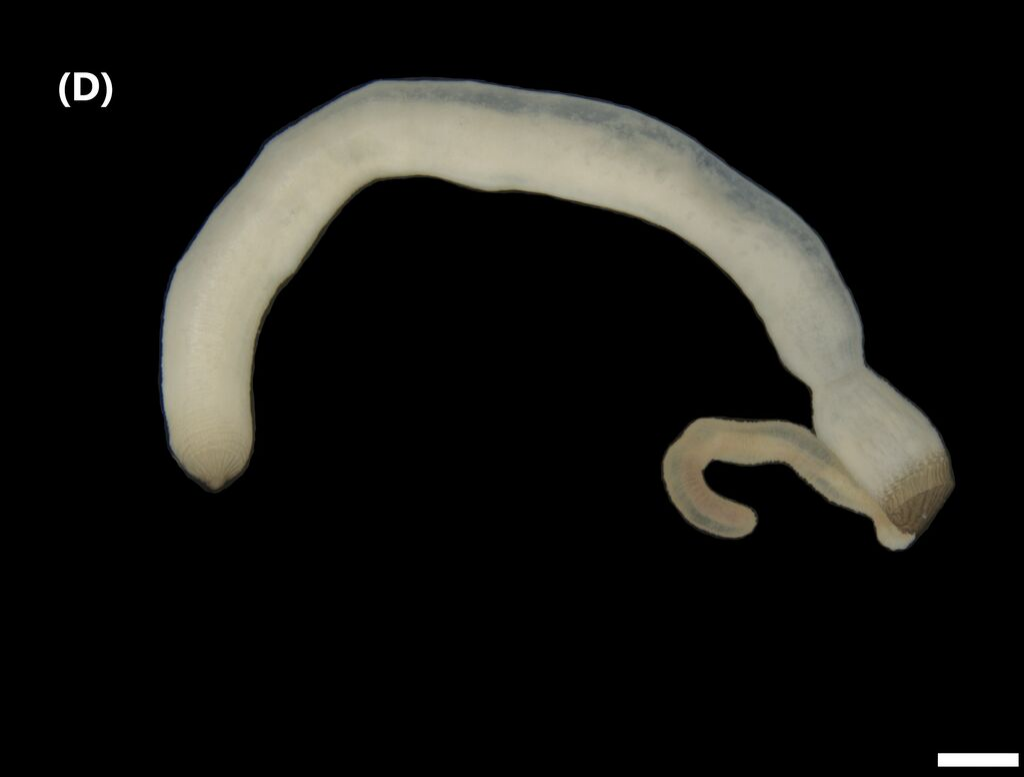
Aspidosiphon (Paraspidosiphon) steenstrupii Diesing, 1859

**Figure 7e.**
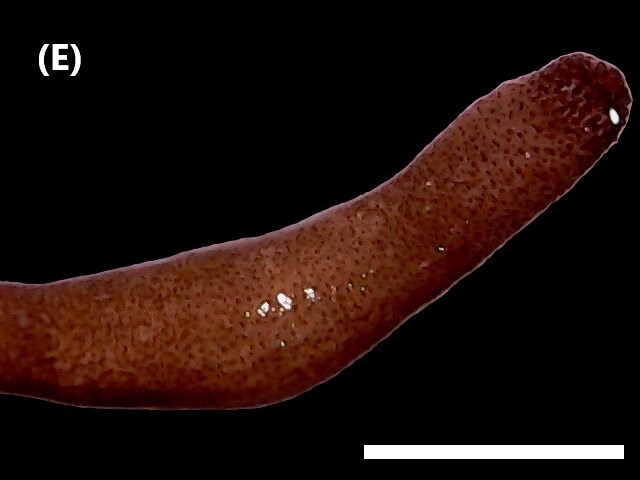
Phascolosoma (Phascolosoma) nigrescens (Keferstein, 1865)

**Figure 7f.**
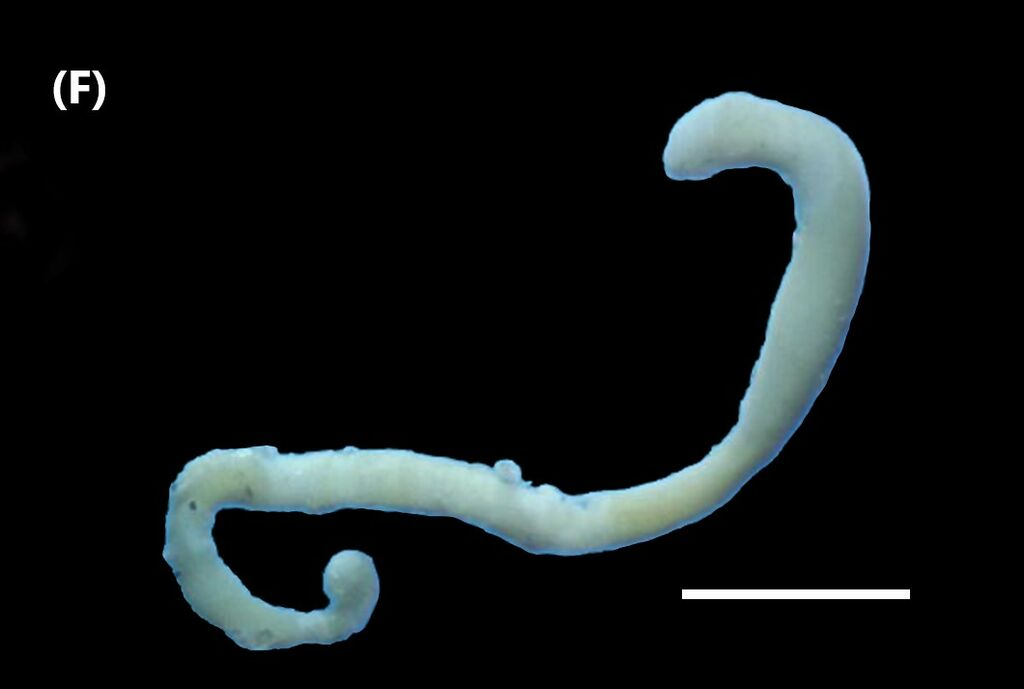
Sipunculus (Sipunculus) phalloides Pallas, 1774

**Figure 8a.**
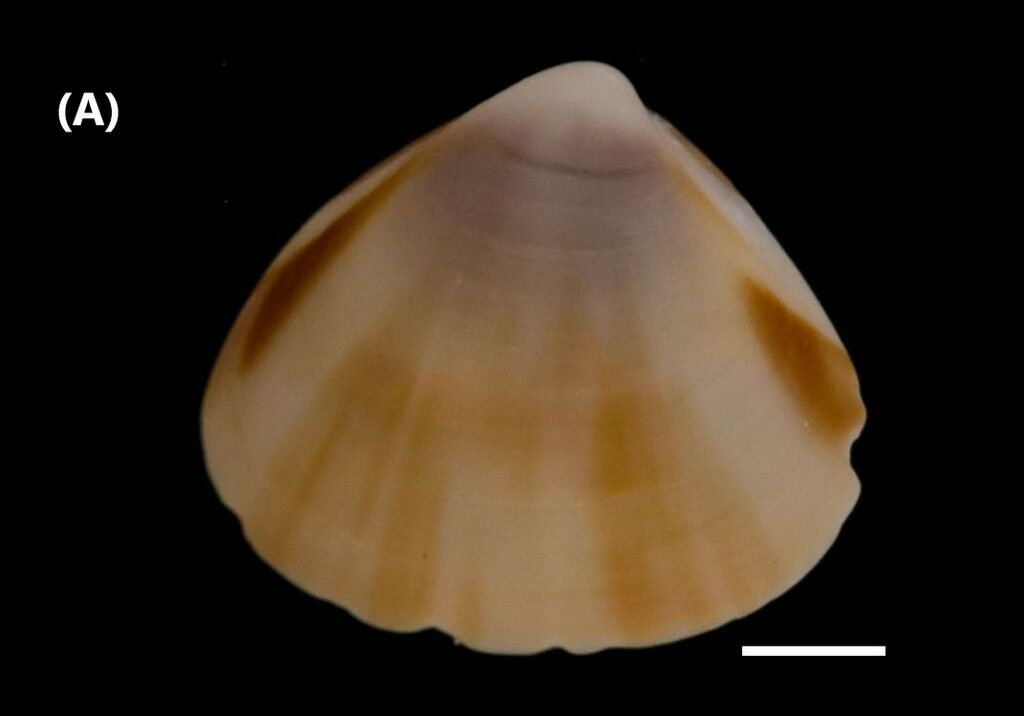
*Muliniacleryana* (d'Orbigny, 1846)

**Figure 8b.**
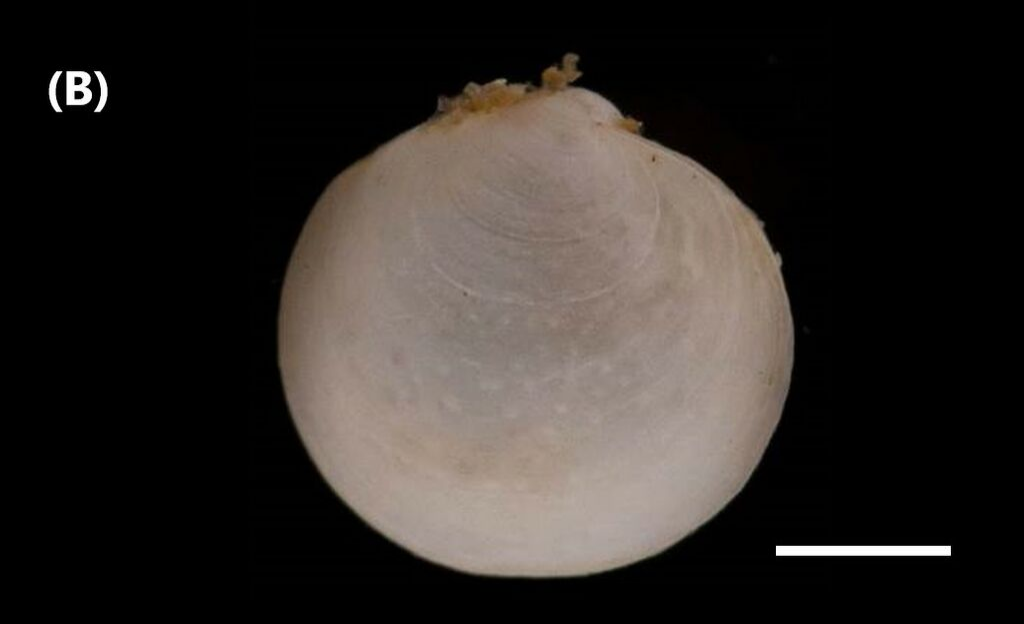
*Phlyctidermasemiasperum* (Philippi, 1836)

**Figure 8c.**
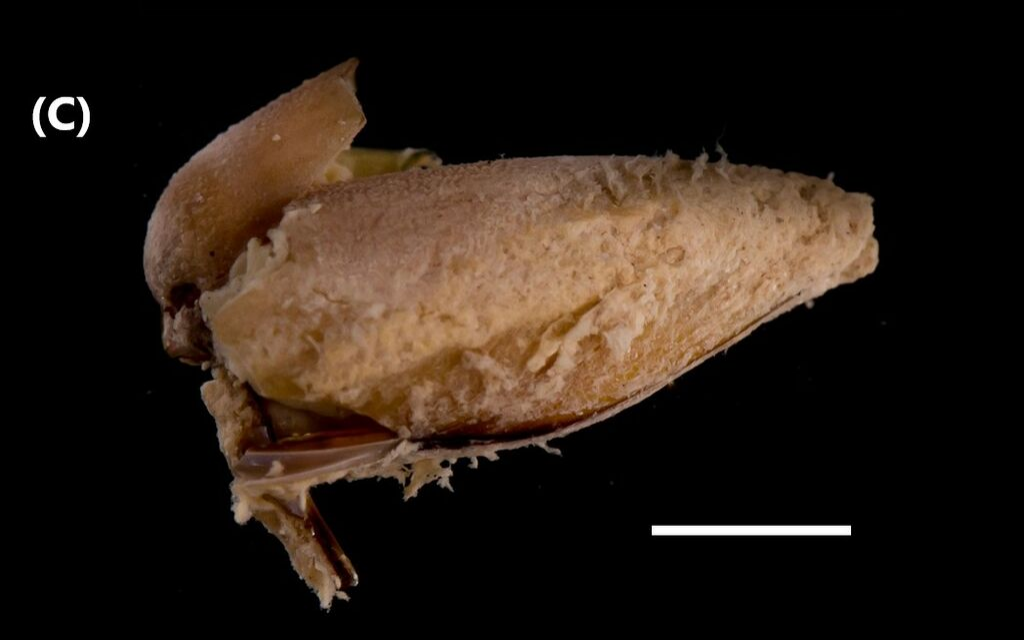
*Brachidontesexustus* (Linnaeus, 1758)

**Figure 8d.**
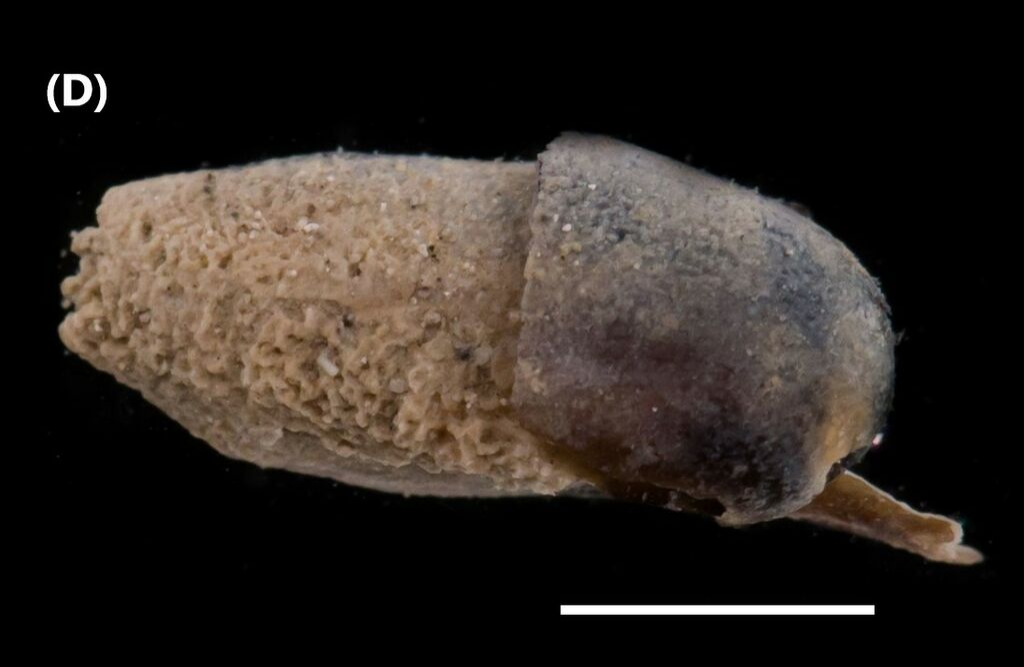
*Mytellastrigata* (Hanley, 1843)

**Figure 8e.**
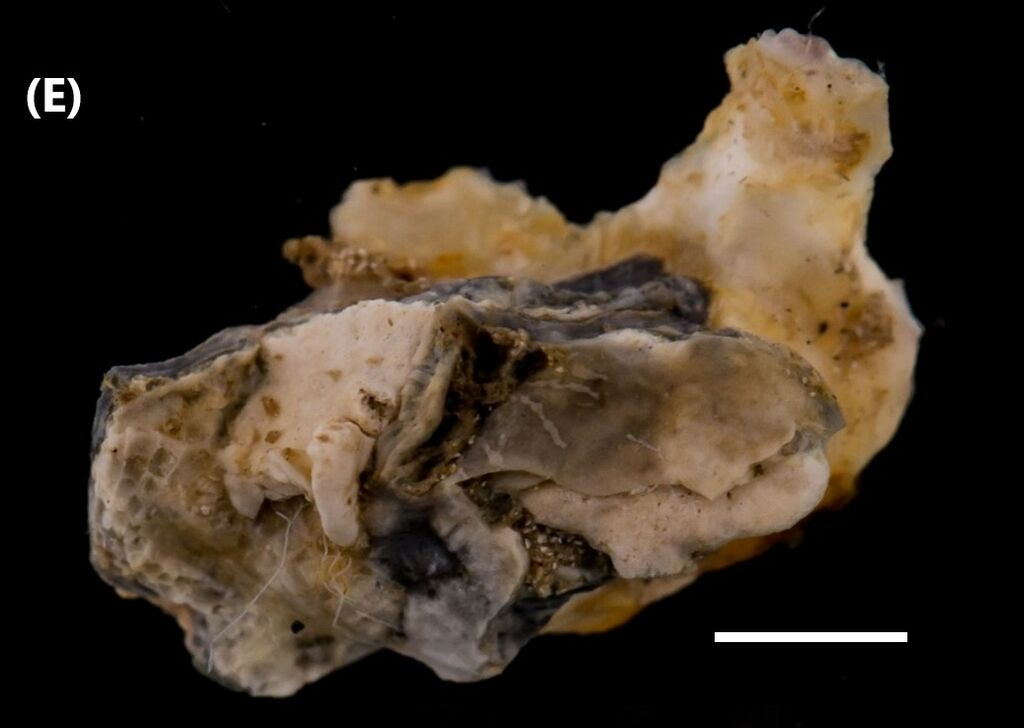
*Crassostreabrasiliana* (Lamarck, 1819)

**Figure 8f.**
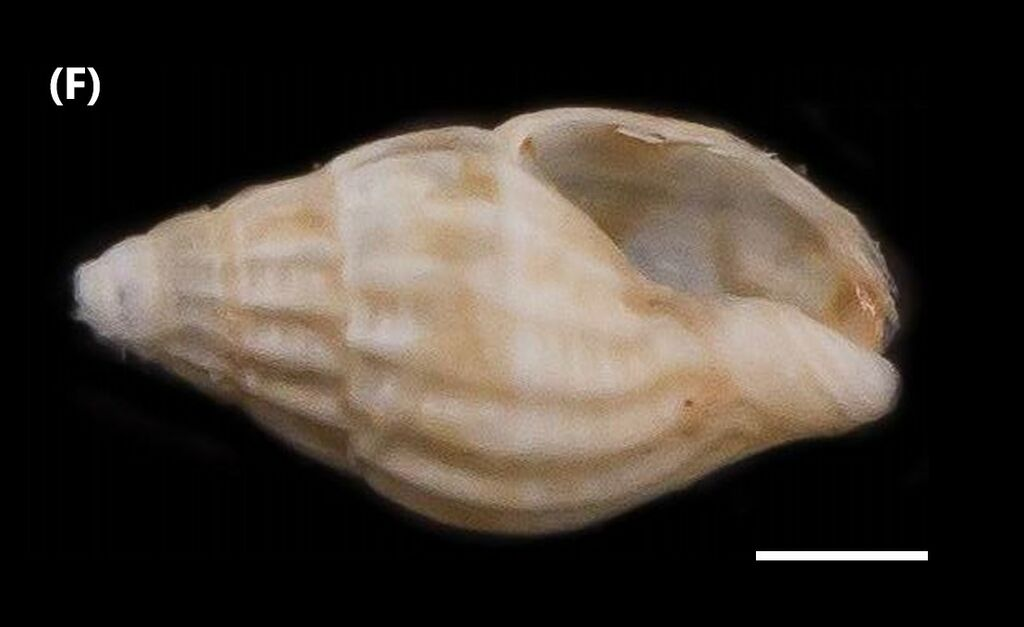
*Parvanachisobesa* (Adams, 1845)

**Figure 9a.**
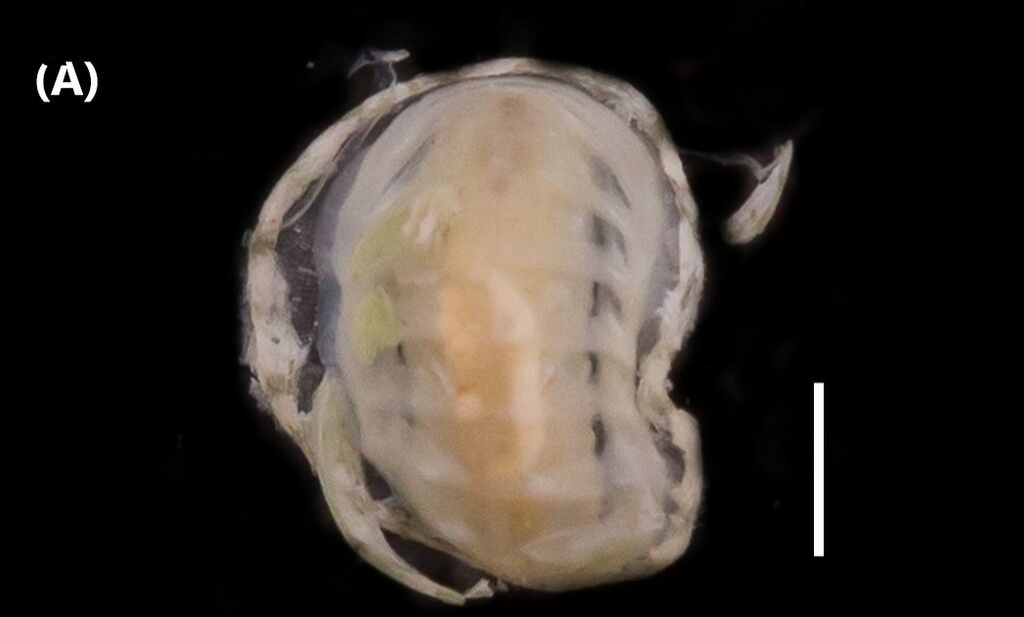
*Acanthochitonaterezae* Guerra Júnior, 1983

**Figure 9b.**
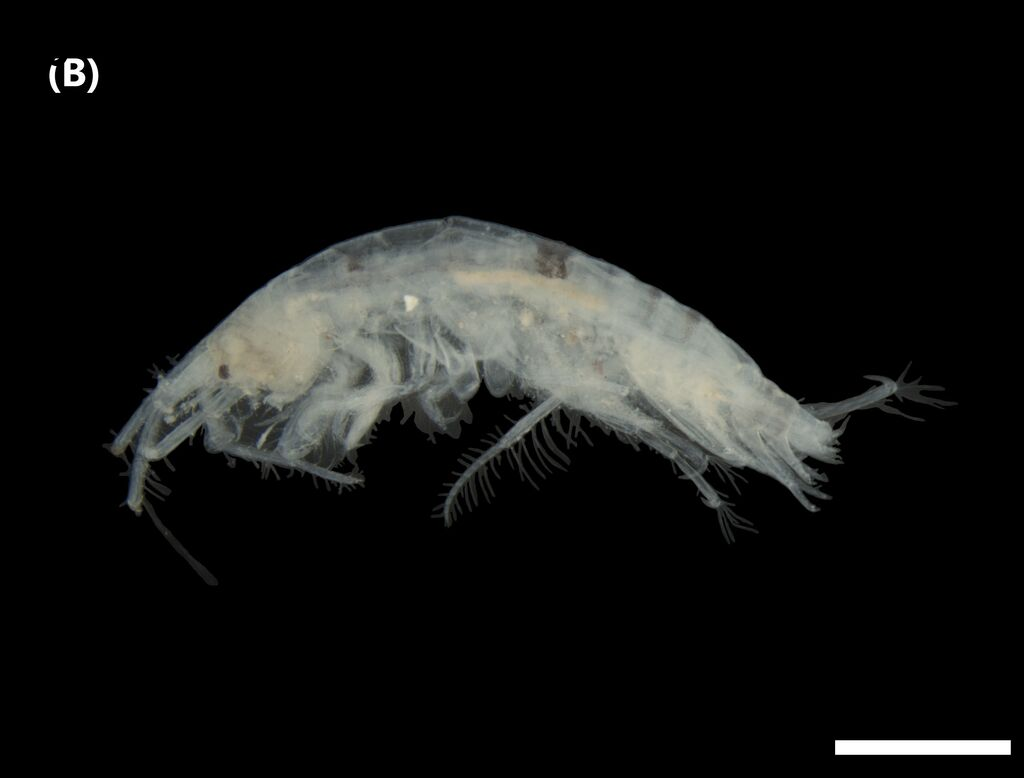
*Elasmopusbrasiliensis* (Dana, 1853)

**Figure 9c.**
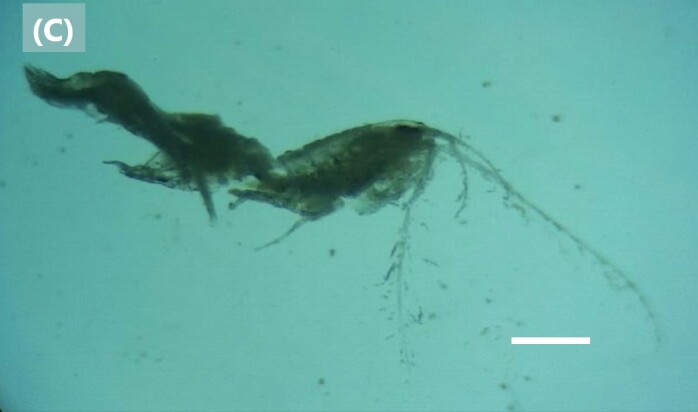
*Dulichiellaappendiculata* (Say, 1818)

**Figure 9d.**
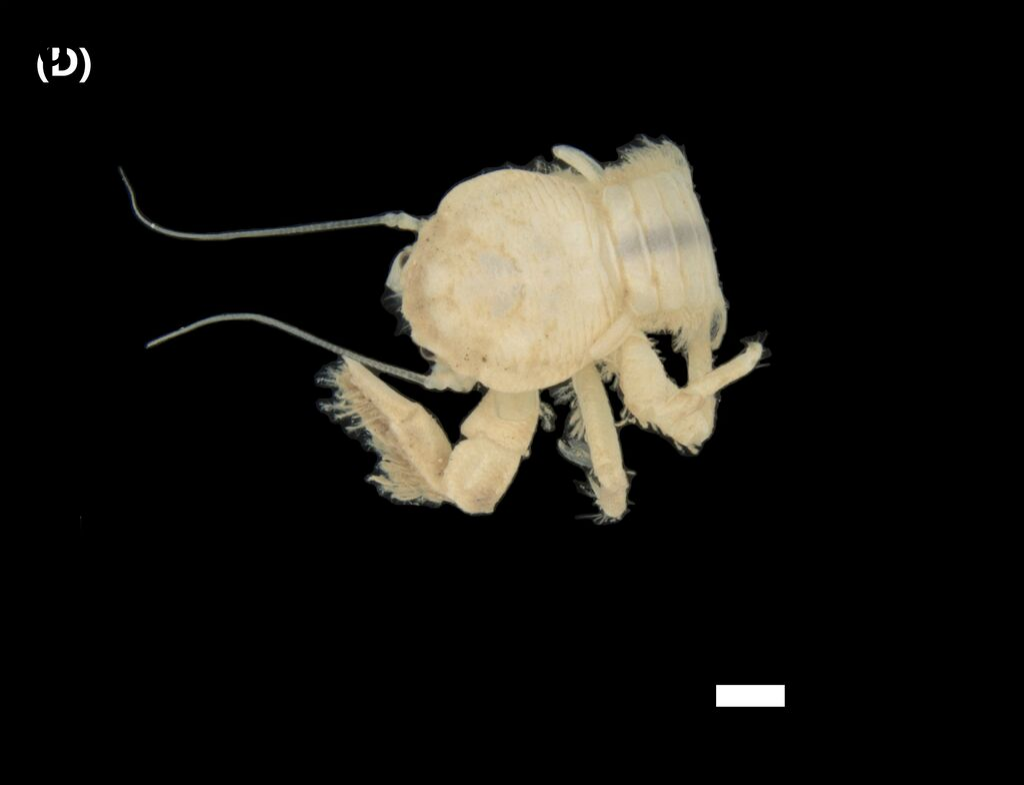
*Cyclodorippelongifrons* Campos Junior & Schmidt de Melo, 1999

**Figure 9e.**
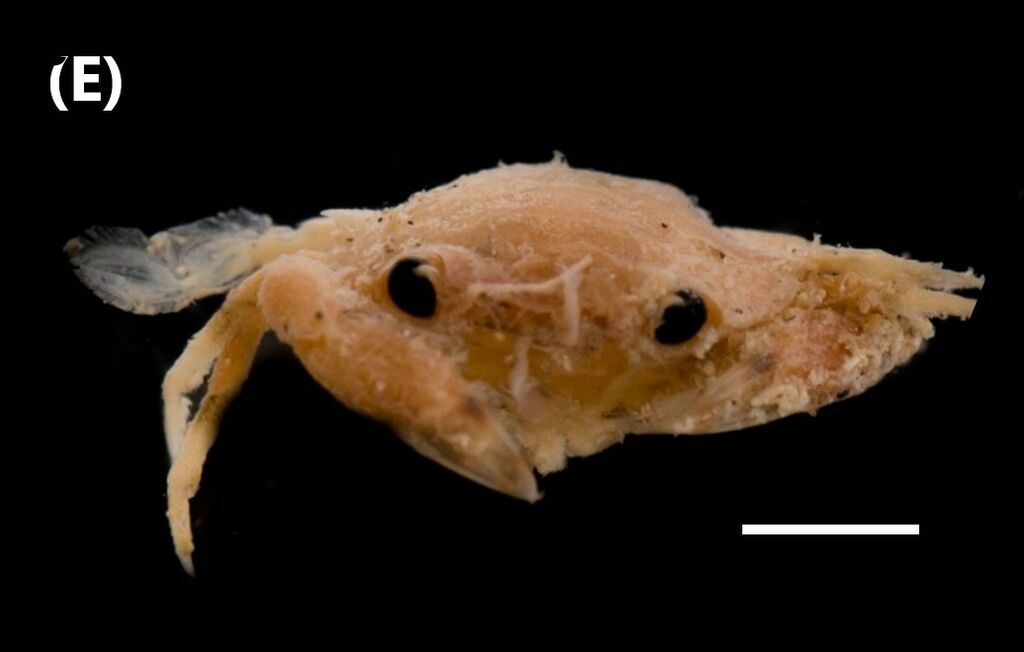
*Mithraculusforceps* Milne-Edwards, 1875

**Figure 10a.**
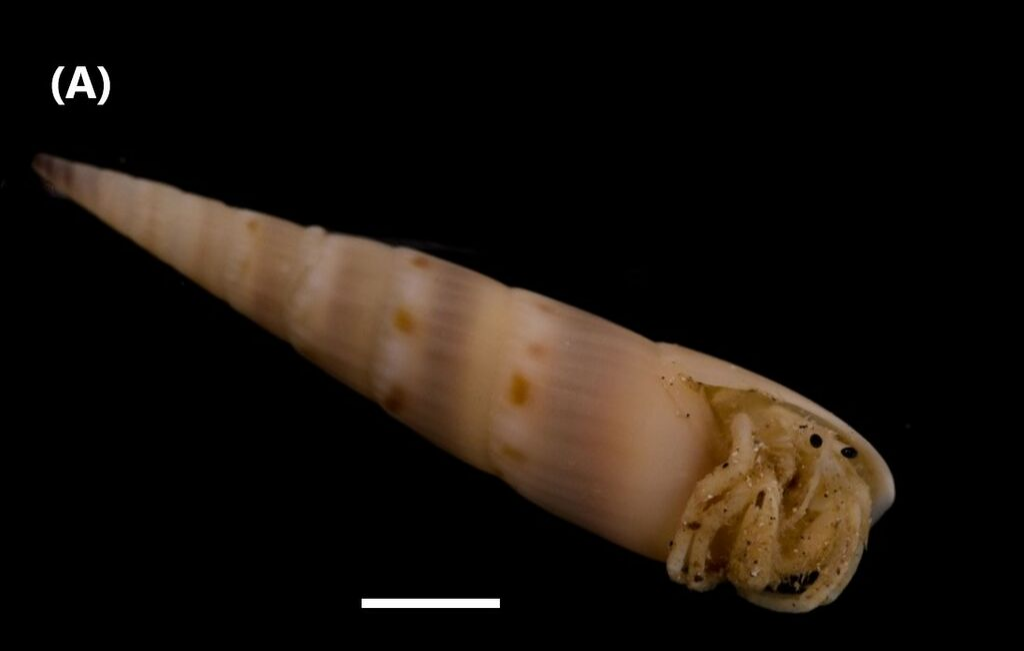
*Paguruscriniticornis* (Dana, 1852)

**Figure 10b.**
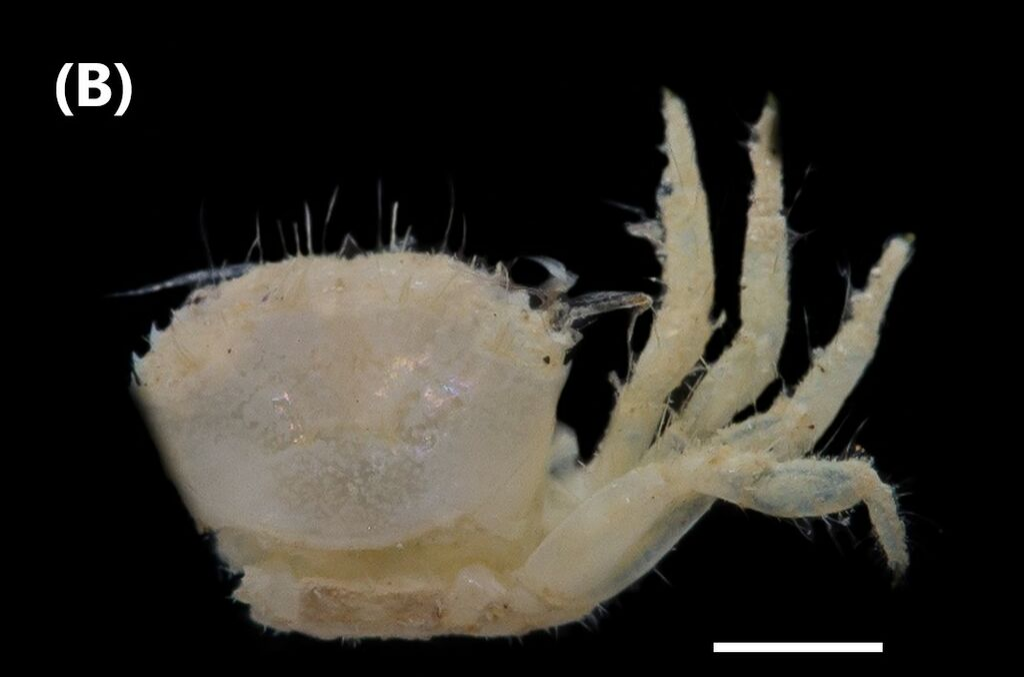
*Garthiopespinipes* (Milne-Edwards, 1880)

**Figure 10c.**
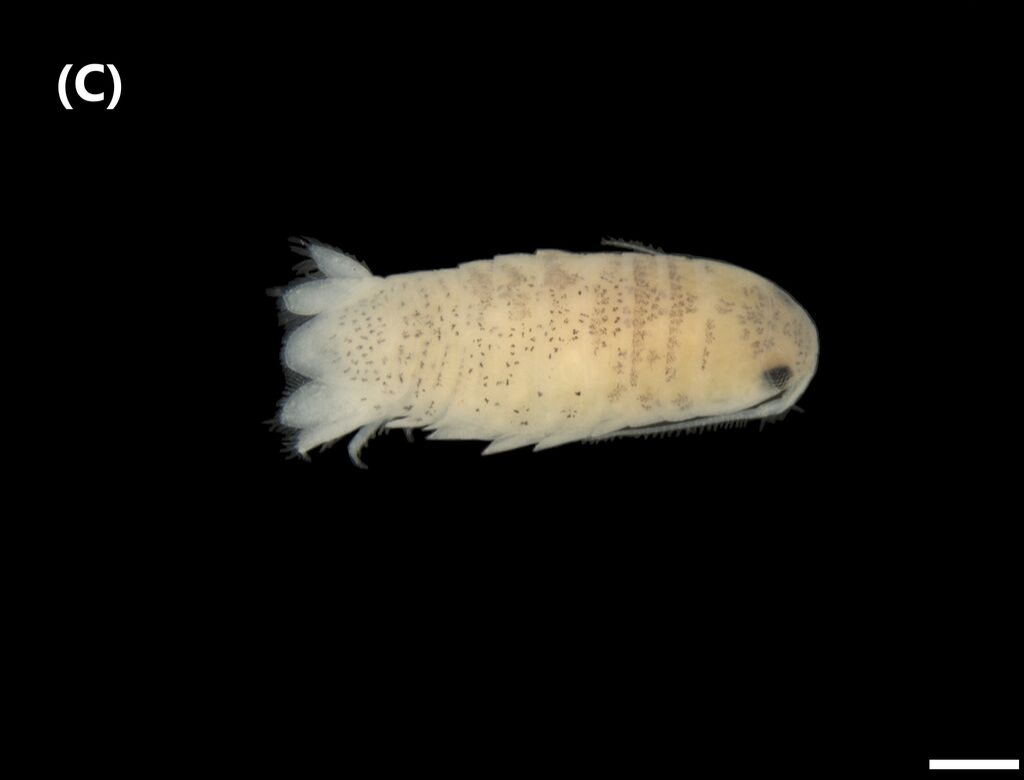
*Cirolanaparva* Hansen, 1890

**Figure 10d.**
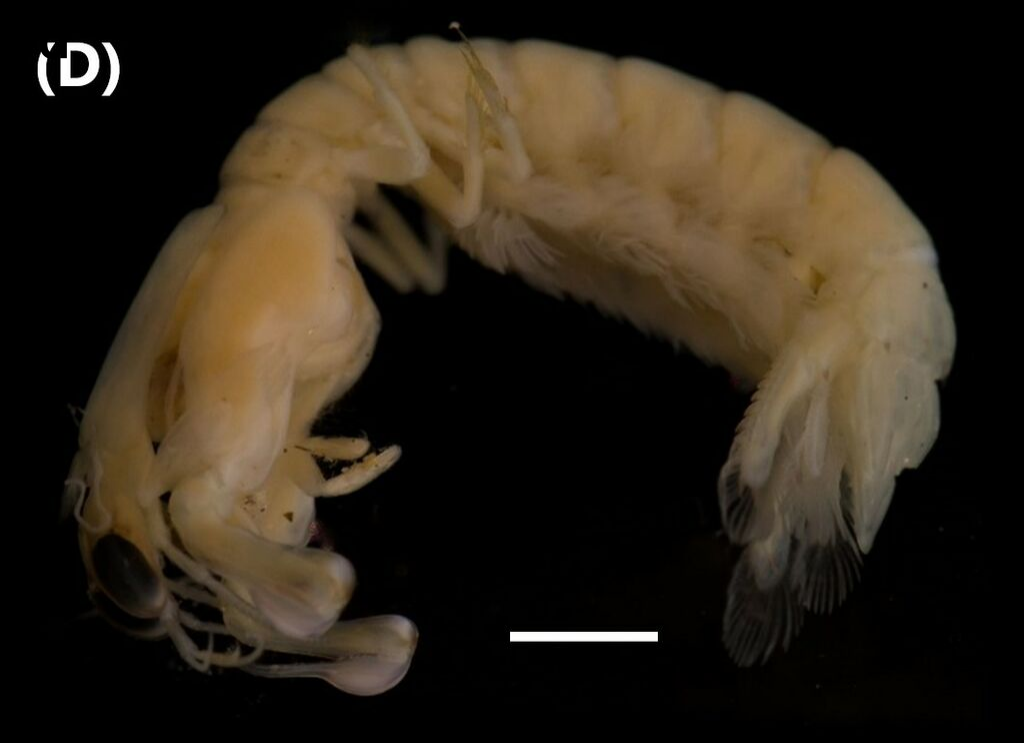
*Neogonodactylustorus* (Manning, 1969)

**Figure 11a.**
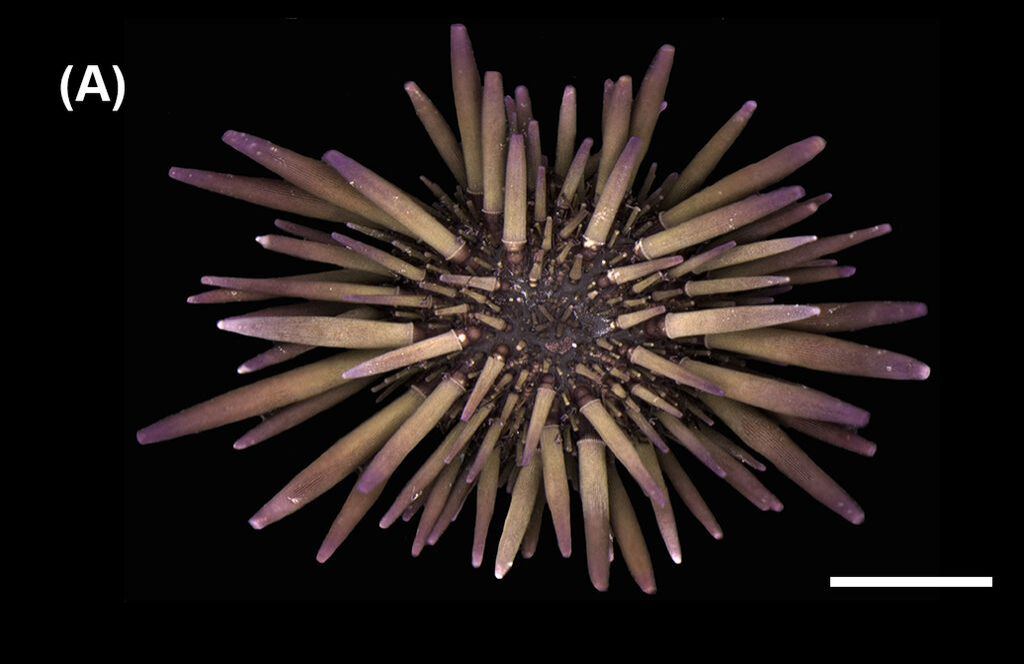
*Echinometralucunter* (Linnaeus, 1758)

**Figure 11b.**
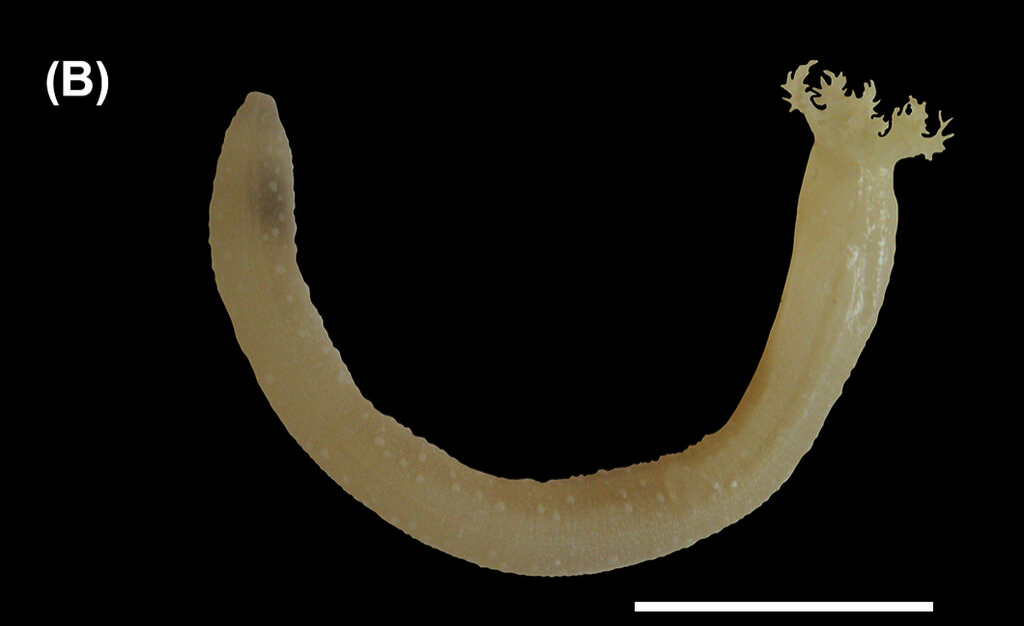
*Chiridotarotifera* (Pourtalès, 1851)

**Figure 11c.**
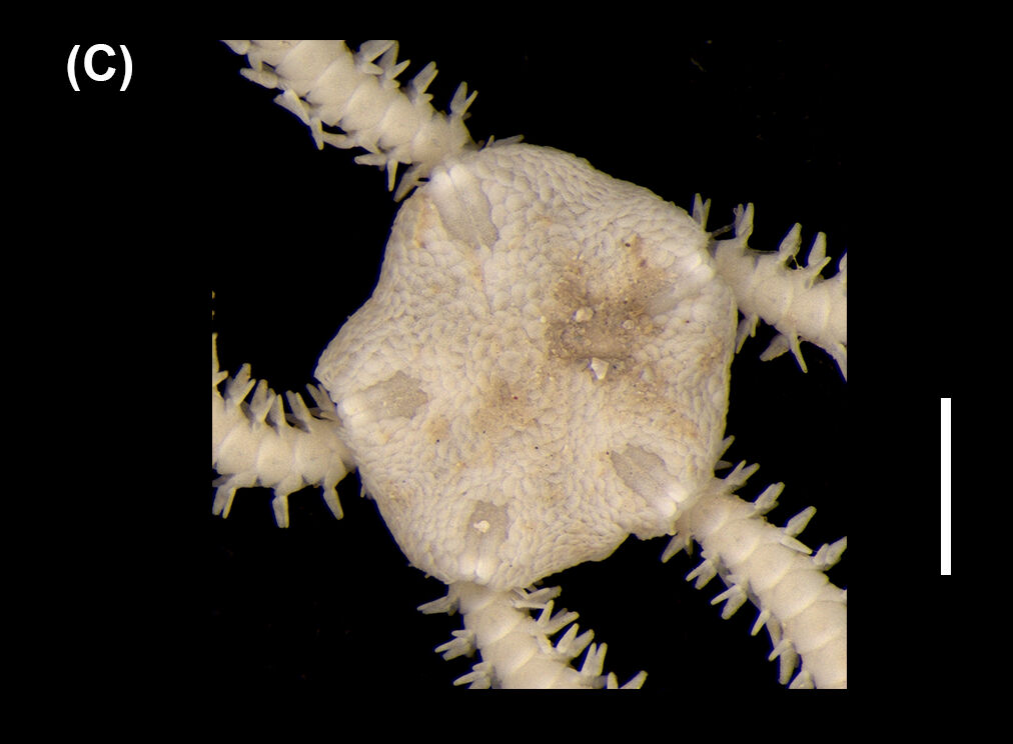
*Amphipholisjanuarii* Ljungman, 1866

**Figure 11d.**
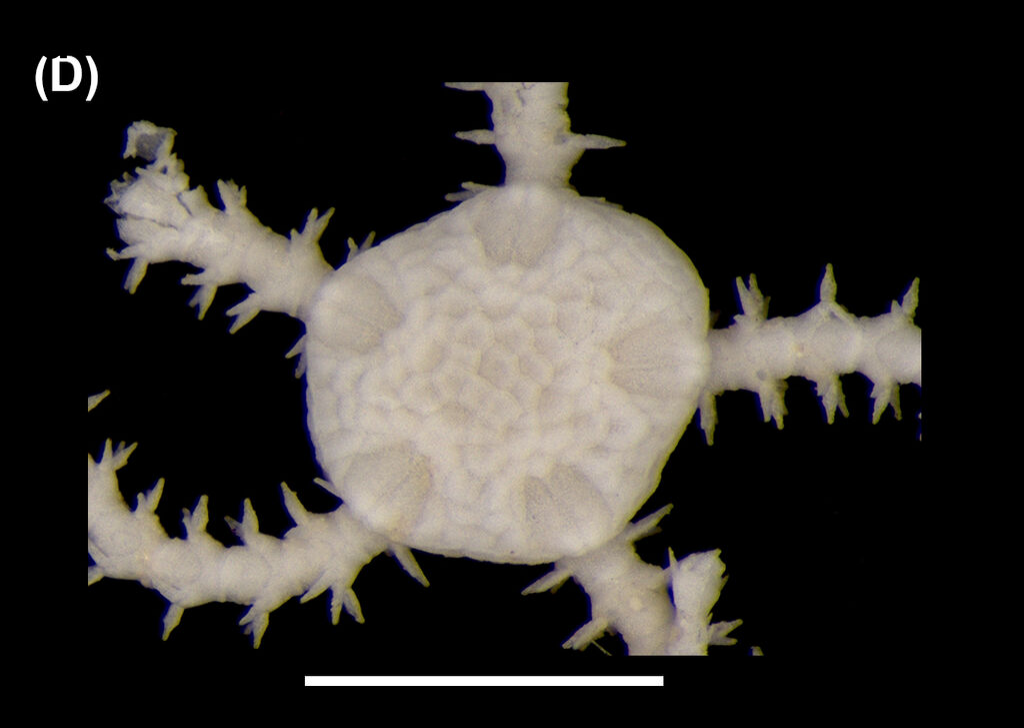
*Amphipholissquamata* (Delle Chiaje, 1828)

**Figure 11e.**
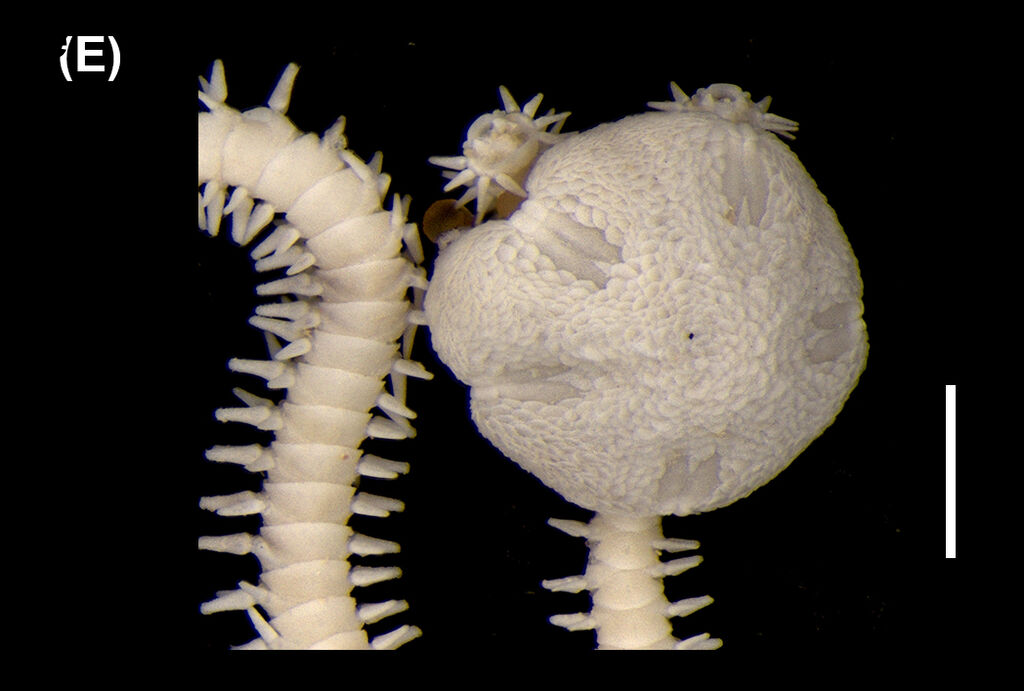
*Microphiopholisgracillima* (Stimpson, 1854)

**Figure 11f.**
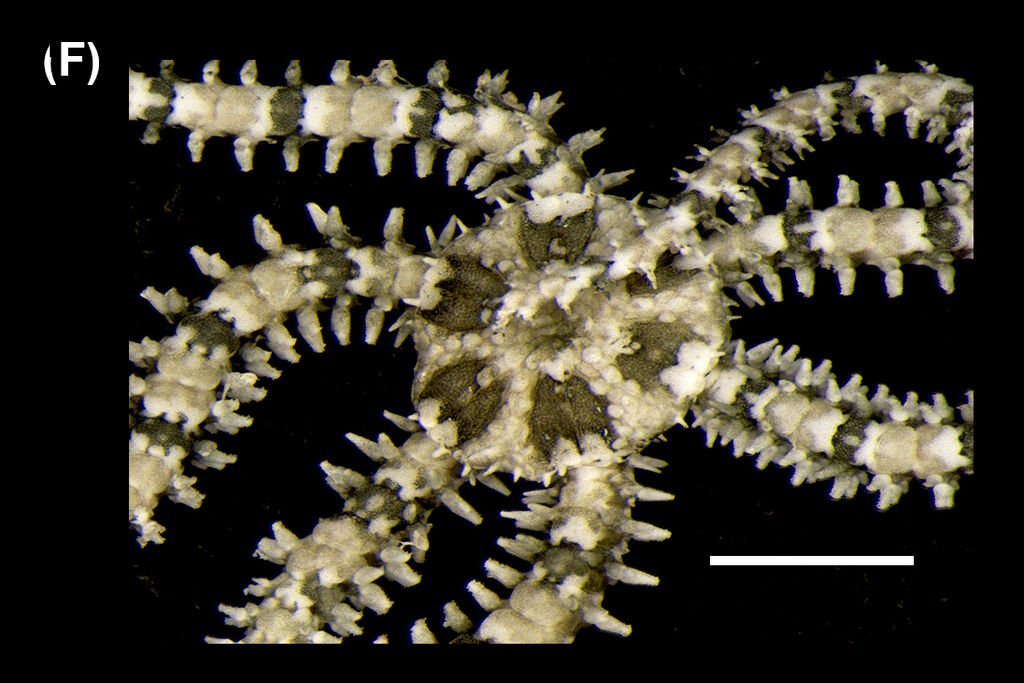
*Ophiactissavignyi* (Müller & Troschel, 1842)
