# Supplementary material for: Marine invertebrates associated with rhodoliths/maërl beds from northeast Brazil (State of Paraíba)
Source: Biodivers Data J. 2021 Jul 21;9:e62736. doi: 10.3897/BDJ.9.e62736 (PMC8390885; doi:10.3897/BDJ.9.e62736)
Supplement: Supplementary material 1 — Authorisation for the collection of invertebrates [file bdj-09-e62736-s001.pdf]

MARINE INVERTEBRATES ASSOCIATED WITH RHODOLITHS/MAËRL BEDS FROM THE NORTHEAST BRAZIL (PARAÍBA STATE)

Dimítri de Araújo Costa, Marina Dolbeth, Jessica Prata, Francisco de Assis da Silva, Geuba Maria Bernardo da Silva, Paulo Ragner Silva de Freitas, Martin Lindsey Christoffersen, Silvio Felipe Barbosa de Lima, Karina Massei, Reinaldo Farias Paiva de Lucena

Supplementary Figure 1. Authorization for the collection of invertebrates, provided by the Brazilian Ministry of the Environment.

ICMBio MMA

Ministério do Meio Ambiente - MMA
Instituto Chico Mendes de Conservação da Biodiversidade - ICMBio
Sistema de Autorização e Informação em Biodiversidade - SISBIO

Dados autorização/relatório

Autorização/Licença: 63971 **Nº relatório periódico:** 25753

Dados do pesquisador titular

Nome	Nacionalidade	CPF	E-mail(s)	Telefone(s)
DIMITRI DE ARAUJO COSTA	Brasileira	058.203.004-83	costa.researcher@yahoo.com.br	

Dados da solicitação

Número	Tipo de solicitação	Título da solicitação
63971	Autorização para atividades com finalidade científica	RELAÇÃO ENTRE A EDUCAÇÃO AMBIENTAL FORMAL E O ESTUDO TAXONÔMICO-SISTEMÁTICO DOS INVERTEBRADOS MARINHOS
